# RAD51AP1-MYC-Sam68 positive transcriptional feedback loop maintains lung cancer stemness

**DOI:** 10.1016/j.isci.2026.117397

**Published:** 2026-08-31

**Authors:** Renwang Liu, Mingbiao Li, Zixuan Hu, Shen Yang, Guangsheng Zhu, Peijun Cao, Haiyang Fan, Jianfang Wang, Zitong Zhang, Jun Chen

**Affiliations:** 1 Department of Lung Cancer Surgery, Center of Thoracic Surgery, Tianjin Medical University General Hospital, Tianjin 300052, China; 2 Tianjin Key Laboratory of Lung Cancer Metastasis and Tumor Microenvironment, Tianjin Lung Cancer Institute, Tianjin Medical University General Hospital, Tianjin 300052, China; 3 Department of Esophagus Surgery, Key Laboratory of Prevention and Therapy, National Clinical Research Center of Cancer, Tianjin Medical University Cancer Institute and Hospital, Tianjin 300060, China; 4 Department of Thoracic Surgery, Sichuan Clinical Research Center for Cancer, Sichuan Cancer Hospital & Institute, Sichuan Cancer Center, University of Electronic Science and Technology of China, Chengdu 610041, China; 5 Department of Thoracic Surgery, Seventh Hospital, Sun Yat-sen University, Shenzhen 518107, China

**Keywords:** lung cancer, cancer stem cells, cancer stemness, RAD51AP1, positive feedback loop, transcriptional regulation

## Abstract

Maintenance of cancer stemness drives lung cancer recurrence, metastasis, and refractoriness. Limited drug efficacy against existing stemness mechanisms suggests the existence of undiscovered complex regulatory networks. Previously, we found that *RAD51AP1* is positively associated with lung cancer stemness; here, we showed that it promotes stemness maintenance *in vitro* and *in vivo*. Mechanistically, using cleavage under targets and tagmentation (CUT&Tag), dual-luciferase reporter assay (DLRA), DNA pull-down-WB, and chromatin immunoprecipitation (ChIP)-qPCR, we confirmed that RAD51AP1 transcriptionally upregulates *MYC* by binding to its promoter region, thereby enhancing stemness. Using a DNA pull-down assay combined with liquid chromatography-mass spectrometry (LC-MS), DLRA, and ChIP-qPCR, we verified that Sam68 binds the *RAD51AP1* promoter to enhance its transcription. More importantly, MYC, conversely, targets the *Sam68* promoter and upregulates its transcription. Thus, we identified a lung cancer stemness regulatory model: the RAD51AP1-MYC-Sam68-RAD51AP1 positive transcriptional feedback loop. Targeting this cascade has the potential to reverse cancer stemness and advance clinical therapies.

## Highlights

• RAD51AP1 directly activates *MYC* transcription and promotes lung cancer stemness
• MYC directly activates Sam68 expression by binding its promoter
• Sam68 directly activates *RAD51AP1* transcription through promoter binding
• The RAD51AP1-MYC-Sam68 transcriptional loop sustains lung cancer stemness

## Graphical abstract

**Figure:**
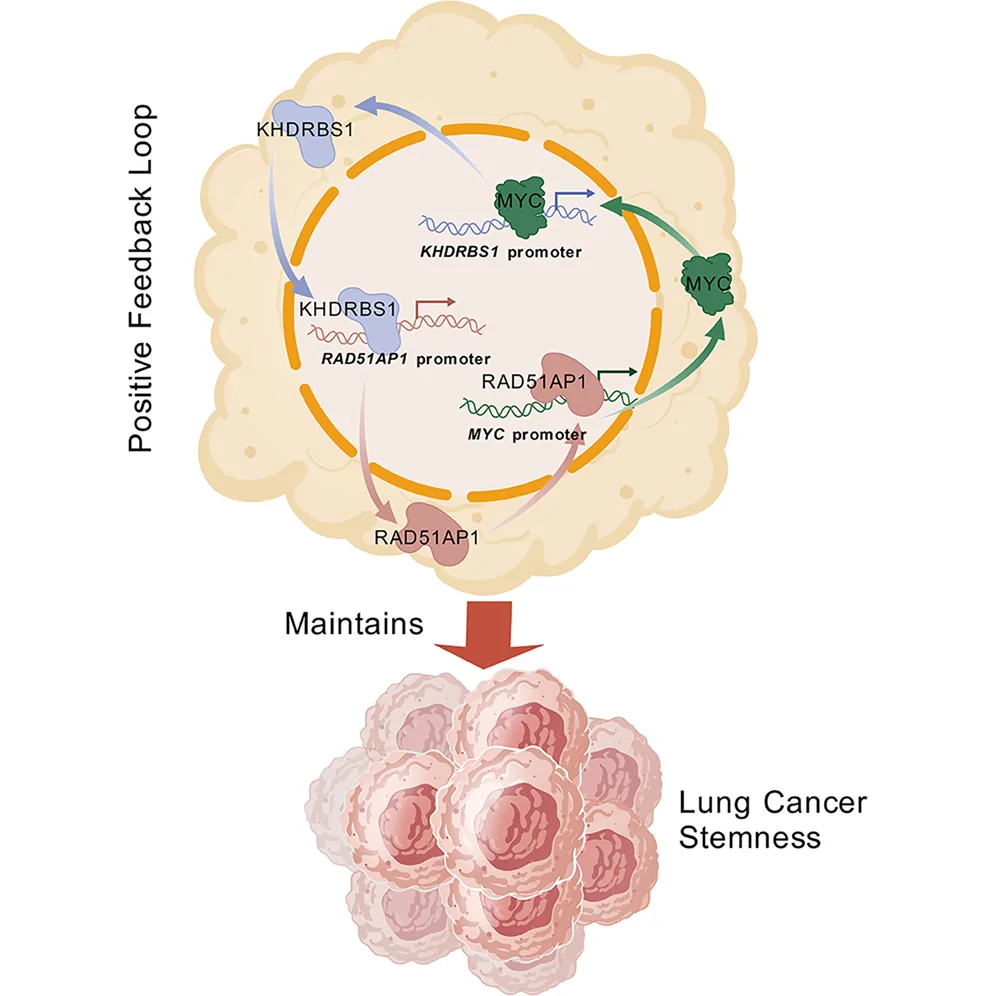

## Teaser

Molecular biology; Cell biology; Stem cells research; Cancer

## Introduction

Lung cancer remains one of the most prevalent and deadly cancers.^1^ Despite significant advancements in its treatment in recent decades, many patients still develop drug resistance and progression.^2^ This progression is largely attributed to maintenance of cancer stemness, which is closely linked to cancer stem cells (CSCs), a subpopulation of cancer cells with self-renewal and differentiation capacities.^3^ Notably, upregulation of classical pathways (Wnt, Notch, and Shh) is a key mechanism underlying this stemness maintenance.^4^ However, existing drugs that target these pathways have shown limited efficacy, indicating more complex regulatory mechanisms in CSCs.^5^ Thus, characterizing additional stemness-regulatory axes is critical for developing effective lung cancer therapies.

RAD51AP1, as a vital protein in homologous recombination repair (HRR)—a process crucial for maintaining genomic stability^6^—mainly recruits and promotes the binding of RAD51 to single-end invasion intermediate (SEI) DNA, thereby stimulating D-loop formation.^7^ It also drives R-loop formation and collaborates with RAD51 to form DR-loops, enhancing HR efficiency under transcriptionally active conditions.^8^ Recently, RAD51AP1 has been reported to promote cancer progression and is associated with poorer prognosis in multiple cancers, including hepatocellular carcinoma (HCC), ovarian cancer, and pancreatic cancer.^9^^,^^10^^,^^11^^,^^12^^,^^13^^,^^14^

Stemness maintenance is presumably one of the pivotal mechanisms by which RAD51AP1 facilitates tumor malignancy. For example, Bridges et al. demonstrated that RAD51AP1 markedly sustains CSC self-renewal, thereby promoting tumor progression in breast and colorectal cancer.^15^^,^^16^ Zeng et al. also verified that knocking down RAD51AP1 enhances chemosensitivity by inhibiting the self-renewal of ovarian CSCs.^17^ Additionally, Redmer et al. found that RAD51AP1 may be closely associated with stemness maintenance in melanoma.^18^

The role of RAD51AP1 in maintaining cancer stemness and its underlying mechanism is still unclear. Our previous study showed that *RAD51AP1* is positively correlated with lung cancer stemness.^19^ Here, we confirmed that RAD51AP1 maintains lung cancer stemness and identified the regulatory mechanism: a RAD51AP1-MYC-Sam68-RAD51AP1 positive transcriptional feedback loop.

MYC, as a major transcription factor in CSCs, is typically overexpressed across cancers to sustain cancer stemness.^20^^,^^21^ In lung cancer, it even facilitates the histological transformation of lung adenocarcinoma (LUAD) to small cell lung cancer (SCLC) by orchestrating cancer stemness.^22^ Sam68 is also upregulated in various cancers and correlates with cancer cell self-renewal.^23^^,^^24^^,^^25^ Although its role in lung cancer stemness has not been reported, Sam68 is overexpressed in lung cancer and predicts poor prognosis.^26^ Thus, targeting MYC and/or Sam68 holds promise for advancing lung cancer drug development or for sensitizing existing drugs in theory.

However, to our best knowledge, no drugs targeting MYC and/or Sam68 have been approved for lung cancer to date. Few such clinical trials for other cancers are also in early phases.^27^^,^^28^ A key reason is that MYC, as an intrinsically disordered protein (IDP), lacks a stable tertiary fold,^29^ while Sam68’s druggable targets remain unclear.^27^ Thus, targeting the upstream and downstream regulators of MYC and/or Sam68 may offer a promising breakthrough. The feedback loop identified in our study highlights key regulators of MYC and Sam68, and may thereby address this limitation, supporting subsequent translational research and drug development.

## Results

### RAD51AP1 promotes lung cancer proliferation, migration, and stemness

**Figure 1:**
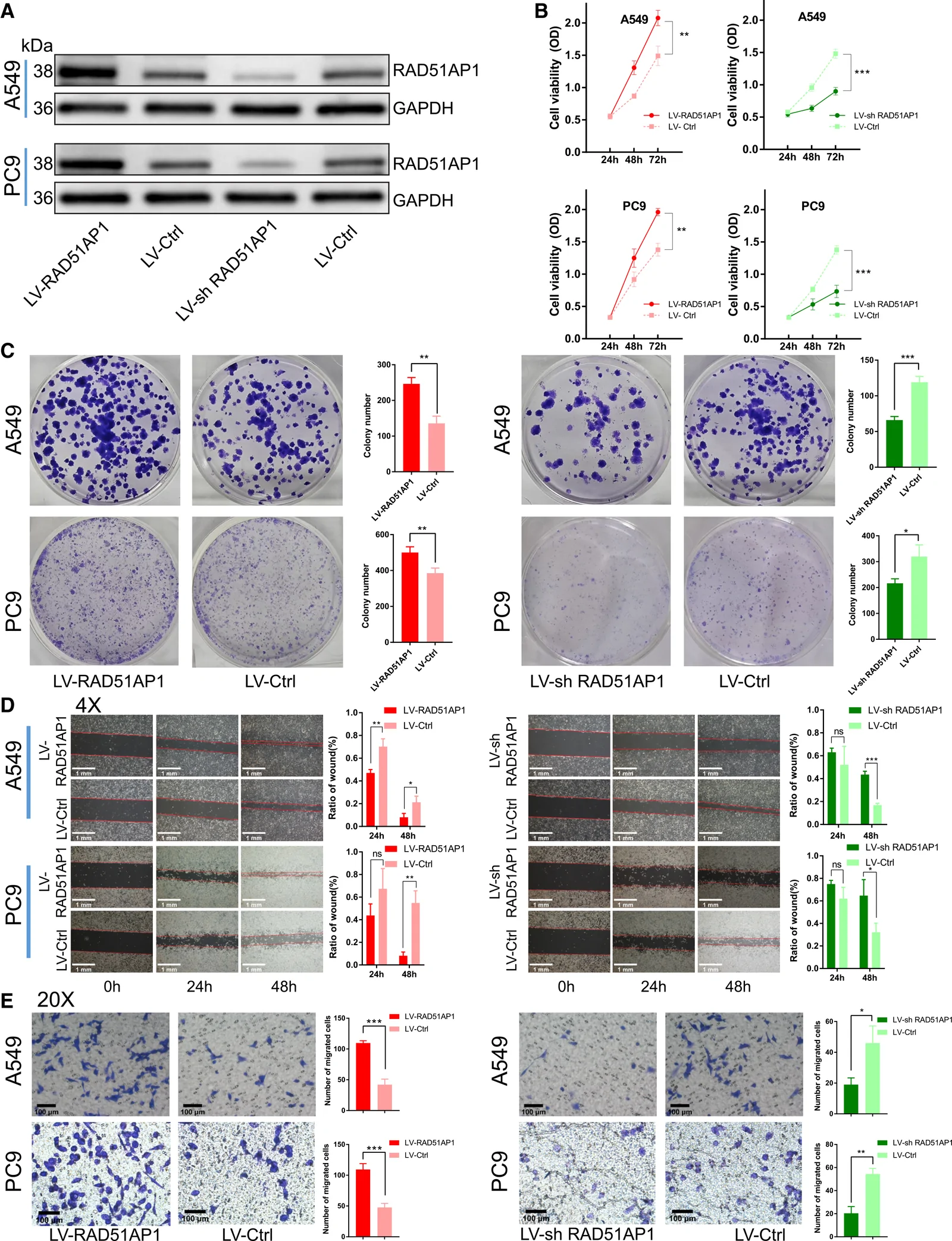
RAD51AP1 promoted lung cancer cell proliferation and migration (A) Lentiviral transfection generated RAD51AP1-overexpressing and -knockdown stable A549 and PC9 cell lines, with efficiency validated by WB. (B) CCK8 assays at 24, 48, and 72 h showed that RAD51AP1 overexpression enhanced cell viability, whereas knockdown reduced it in these cells. (C) Colony formation assays revealed that RAD51AP1 overexpression increased, and knockdown decreased, colony counts in these cells. (D) Wound healing assays showed that RAD51AP1 overexpression increased the wound closure ratio in these cells at 48 h, with knockdown exerting the opposite effect. Scale bars, 1 mm. (E) Transwell migration assays showed that RAD51AP1 overexpression significantly increased the number of migrated cells, while knockdown decreased them. Scale bars, 100 μm. All experiments were performed in triplicate. Data are represented as mean ± SD. Statistical significance was assessed using two-way ANOVA for (B); Student’s *t* test for (C), (E), all PC9 comparisons in (D), and the A549 LV-shRAD51AP1 versus LV-Ctrl comparison at 24 h in (D); and the Mann-Whitney U test for all A549 LV-RAD51AP1 versus LV-Ctrl comparisons and the A549 LV-shRAD51AP1 versus LV-Ctrl comparison at 48 h in (D). ns: *p* ≥ 0.05; ∗: *p* < 0.05; ∗∗: *p* < 0.01; ∗∗∗: *p* < 0.001.

**Figure 2:**
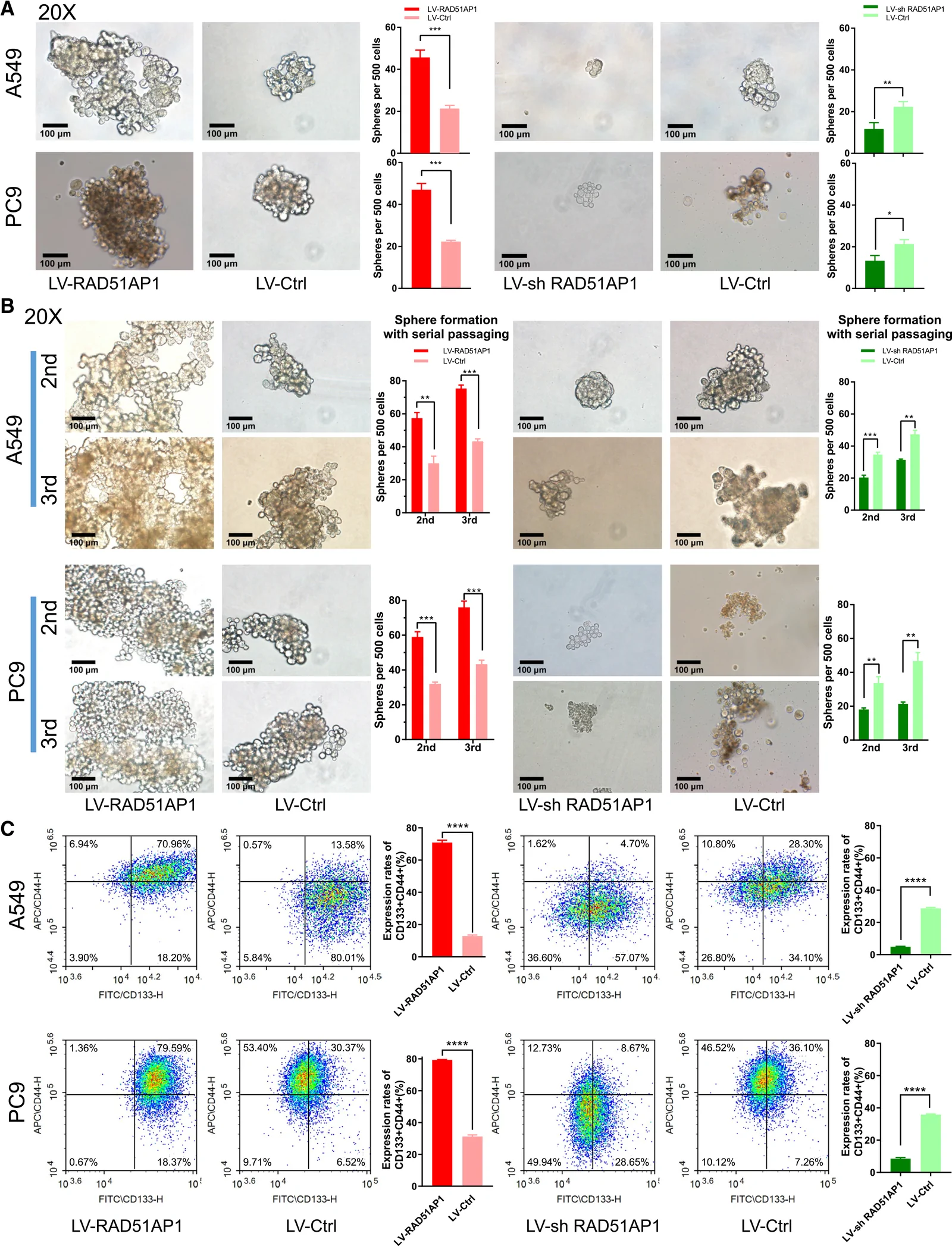
RAD51AP1 enhanced lung cancer cell stemness (A) Sphere formation assays showed that RAD51AP1 significantly increased the sphere volume and sphere-forming efficiency (SFE) in A549 and PC9 cells. Scale bars, 100 μm. (B) Cancer stem cells (CSCs) were isolated from these spheres using 40 μm cell strainers. Serial passage sphere-forming assays (secondary and tertiary) further showed that RAD51AP1 increased sphere volume and SFE in lung CSCs. Scale bars, 100 μm. (C) Flow cytometry showed that overexpressing RAD51AP1 in A549 and PC9 cells significantly increased the proportion of CD44^+^CD133^+^ subsets (*p* < 0.05). In contrast, knockdown of RAD51AP1 significantly decreased the proportion of these subsets (*p* < 0.05). All experiments were performed in triplicate. Data are represented as mean ± SD. Statistical significance was assessed using Student’s *t* test for (A) and (C) and the Mann-Whitney U test for (B). ∗: *p* < 0.05; ∗∗: *p* < 0.01; ∗∗∗: *p* < 0.001; ∗∗∗∗: *p* < 0.0001.

We verified the lentiviral transfection efficiency of RAD51AP1 in A549 and PC9 cells by WB (Figure 1A). Using CCK8 and colony formation assays, we demonstrated that RAD51AP1 promotes lung cancer cell proliferation (Figures 1B and 1C). Similarly, wound healing and Transwell migration assays confirmed its ability to enhance cell migration (Figures 1D and 1E). Furthermore, sphere formation and serial passage assays revealed that RAD51AP1 significantly improves the sphere-forming capacity of these cells (Figures 2A and 2B). Flow cytometry showed that RAD51AP1 significantly increased the proportion of CD44^+^CD133^+^ subsets in A549 and PC9 cells (Figure 2C).

**Figure 3:**
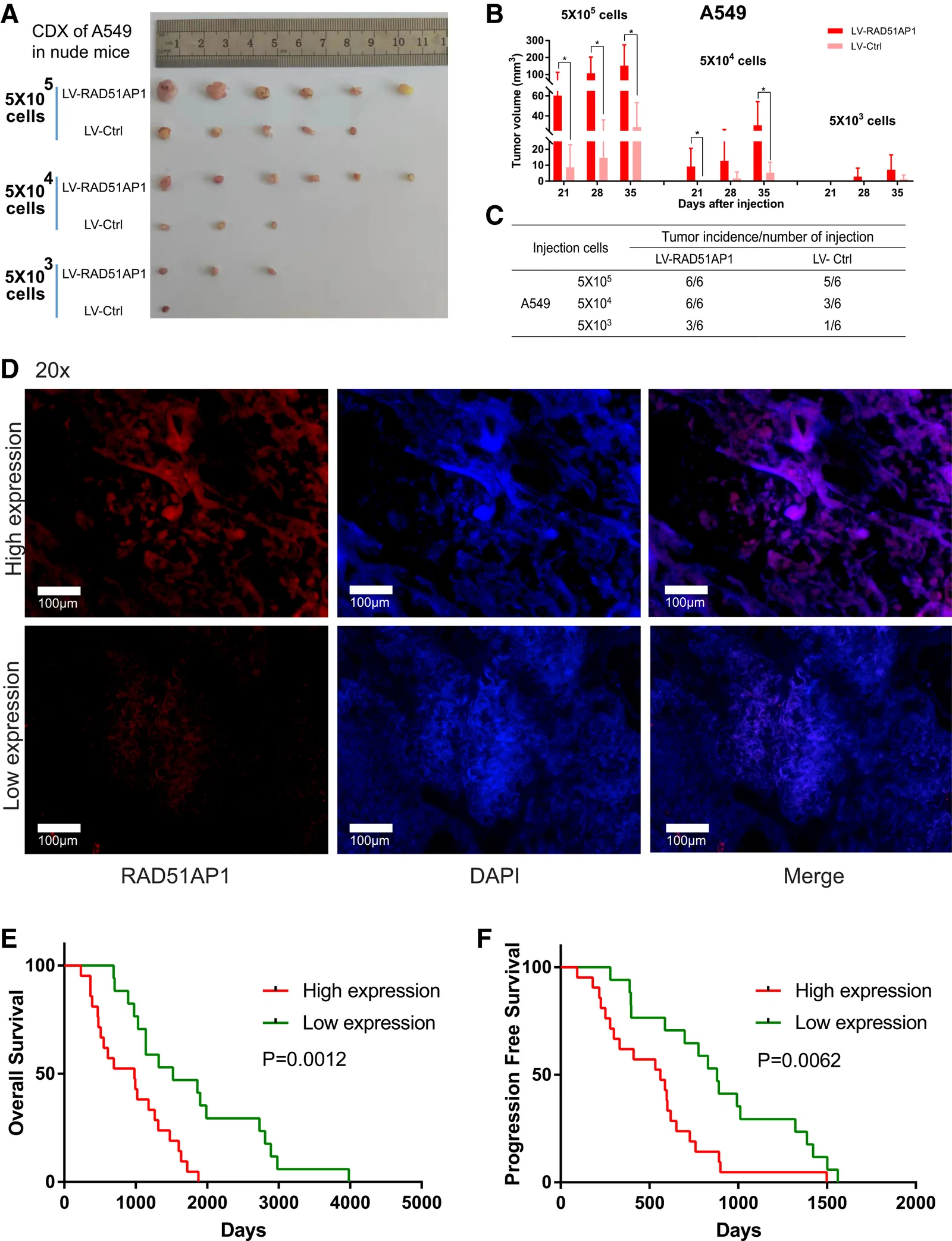
RAD51AP1 promoted A549 cell stemness *in vivo* and correlated with poor prognosis (A–C) To evaluate *in vivo* stemness, cell-derived xenografts (CDXs) with different cell densities (5 × 10^5^, 5 × 10^4^, 5 × 10^3^) were established in BALB/c nude mice (*n* = 6 per group). RAD51AP1-overexpressing A549 stable transfectants (vs. vector control) showed a significantly larger tumor size (A), higher growth rate (B), and increased tumorigenicity (C). (D–F) Tissue samples from 46 lung cancer patients were included (baseline information in Table 1), and RAD51AP1 expression was detected by immunofluorescence (IF). Patients were stratified into high (*n* = 23) and low (*n* = 23) RAD51AP1 expression groups based on the median value (M = 2.5982). Representative IF images of high and low RAD51AP1 expression in lung cancer tissues are shown (D). Scale bars, 100 μm. The clinical characteristics were comparable between the two groups (Table 2). Patients with higher RAD51AP1 expression had significantly shorter OS (E; *p* = 0.0012) and PFS (F; *p* = 0.0062). Data in (B) are represented as mean ± SD. Statistical significance in (B) was assessed using Student’s *t* test for the 5 × 10^5^-cell group at day 21 and the 5 × 10^4^-cell group at day 28, and the Mann-Whitney U test for the remaining comparisons. OS and PFS in (E) and (F) were analyzed using the Kaplan-Meier method and compared using the log rank test. ∗: *p* < 0.05.

In the cell-derived xenograft (CDX) model, tumors from RAD51AP1-overexpressing A549 cells emerged significantly earlier and were more prominent (Figures 3A–3C). To validate this finding in clinical samples, we collected 46 lung cancer tissue samples from our department (baseline details in Table 1). Immunofluorescence (IF) staining detected RAD51AP1 expression, stratifying the patients into high- and low-expression groups (Figure 3D). No significant differences in age, sex, smoking status, histology, or TNM stage were observed between the groups (Table 2). High RAD51AP1 expression was correlated with worse overall survival (OS) (Figure 3E) and progression-free survival (PFS) (Figure 3F).

**Table 1:** Baseline characteristics of 46 enrolled patients

Characteristic	Number	Percentage
**Gender**		

Female	24	52.2%
Male	22	47.8%

**Smoke status**		

Current	18	39.1%
Never	28	60.9%

**Histology**		

Adenocarcinoma	44	95.7%
Ad-SCC	2	4.3%

**Surgical procedure**		

Wedge	6	13.0%
Lobectomy	35	76.1%
Sleeve lobectomy	4	8.7%
Lobectomy with pleural nodules cautery and abrasion	1	2.2%

**Adjuvant treatment**		

With	32	69.6%
Without	14	30.4%

**T stage**		

T1a	2	4.3%
T1b	4	8.7%
T1c	3	6.5%
T2	25	54.3%
T3	8	17.4%
T4	4	8.7%

**N stage**		

N0	23	50.0%
N1	9	19.6%
N2	8	17.4%
N3	6	13.0%

**M stage**		

M0	45	97.8%
M1a	1	2.2%

**TNM staging**		

IA1	2	4.3%
IA2	2	4.3%
IA3	2	4.3%
IB	13	28.3%
IIB	8	17.4%
IIIA	10	21.7%
IIIB	6	13.0%
IIIC	2	4.3%
IVA	1	2.2%

**Progression status**		

Unprogressed	6	13.0%
Progressed	38	82.6%
Censored	2	4.3%

**Survival status**		

Survival	6	13.0%
Dead	38	82.6%
Censored	2	4.3%

Abbreviation: Ad-SCC, adenosquamous carcinoma.

**Table 2:** Comparison of clinical features between high and low RAD51AP1 expression groups

Characteristic	High RAD51AP1 expression (*n* = 23)	Low RAD51AP1 expression (*n* = 23)	*p*
Age^a^	42–74 (yr)	32–75 (yr)	0.733
Gender^b^	–	–	0.376
Male	9 (39.1%)	13 (56.5%)	–
Female	14 (60.9%)	10 (43.5%)	–
Smoke status^b^	–	–	0.130
Current	6 (26.1%)	12 (52.2%)	–
Never	17 (73.9%)	11 (47.8%)	–
Histology^b^	–	–	0.489
Adenocarcinoma	23 (100.0%)	21 (91.3%)	–
Ad-SCC	0 (0.0%)	2 (8.7%)	–
Surgical procedure^c^	–	–	0.370
Wedge	4 (17.4%)	2 (8.7%)	–
Lobectomy	17 (73.9%)	18 (78.3%)	–
Sleeve lobectomy	1 (4.3%)	3 (13.0%)	–
Lobectomy with pleural nodules cautery and pleural abrasion	1 (4.3%)	0 (0.0%)	–
Adjuvant treatment^b^	–	–	1.000
With	16 (69.6%)	16 (69.6%)	–
Without	7 (30.4%)	7 (30.4%)	–
T stage^c^	–	–	0.703
T1a	1 (4.3%)	1 (4.3%)	–
T1b	1 (4.3%)	3 (13.0%)	–
T1c	2 (8.7%)	1 (4.3%)	–
T2	13 (56.5%)	12 (52.2%)	–
T3	5 (21.7%)	3 (13.0%)	–
T4	1 (4.3%)	3 (13.0%)	–
N stage^c^	–	–	0.100
N0	10 (43.5%)	13 (56.5%)	–
N1	4 (17.4%)	5 (21.7%)	–
N2	7 (30.4%)	1 (4.3%)	–
N3	2 (8.7%)	4 (17.4%)	–
M stage^b^	–	–	1.000
M0	22 (95.7%)	23 (100.0%)	–
M1a	1 (4.3%)	0 (0.0%)	–
TNM staging^c^	–	–	0.415
IA1	1 (4.3%)	1 (4.3%)	–
IA2	0 (0.0%)	2 (8.7%)	–
IA3	1 (4.3%)	1 (4.3%)	–
IB	6 (26.1%)	7 (30.4%)	–
IIB	5 (21.7%)	3 (13.0%)	–
IIIA	5 (21.7%)	5 (21.7%)	–
IIIB	4 (17.4%)	2 (8.7%)	–
IIIC	0 (0.0%)	2 (8.7%)	–
IVA	1 (4.3%)	0 (0.0%)	–

Abbreviation: Ad-SCC, adenosquamous carcinoma.

a The Mann-Whitney U test was used to compare age between the two groups.

b Fisher’s exact test was used for categorical comparisons where appropriate.

c The likelihood-ratio chi-square test was used when more than 50% of cells had expected counts of less than 5.

### RAD51AP1 enhances lung cancer stemness by promoting MYC expression

**Figure 4:**
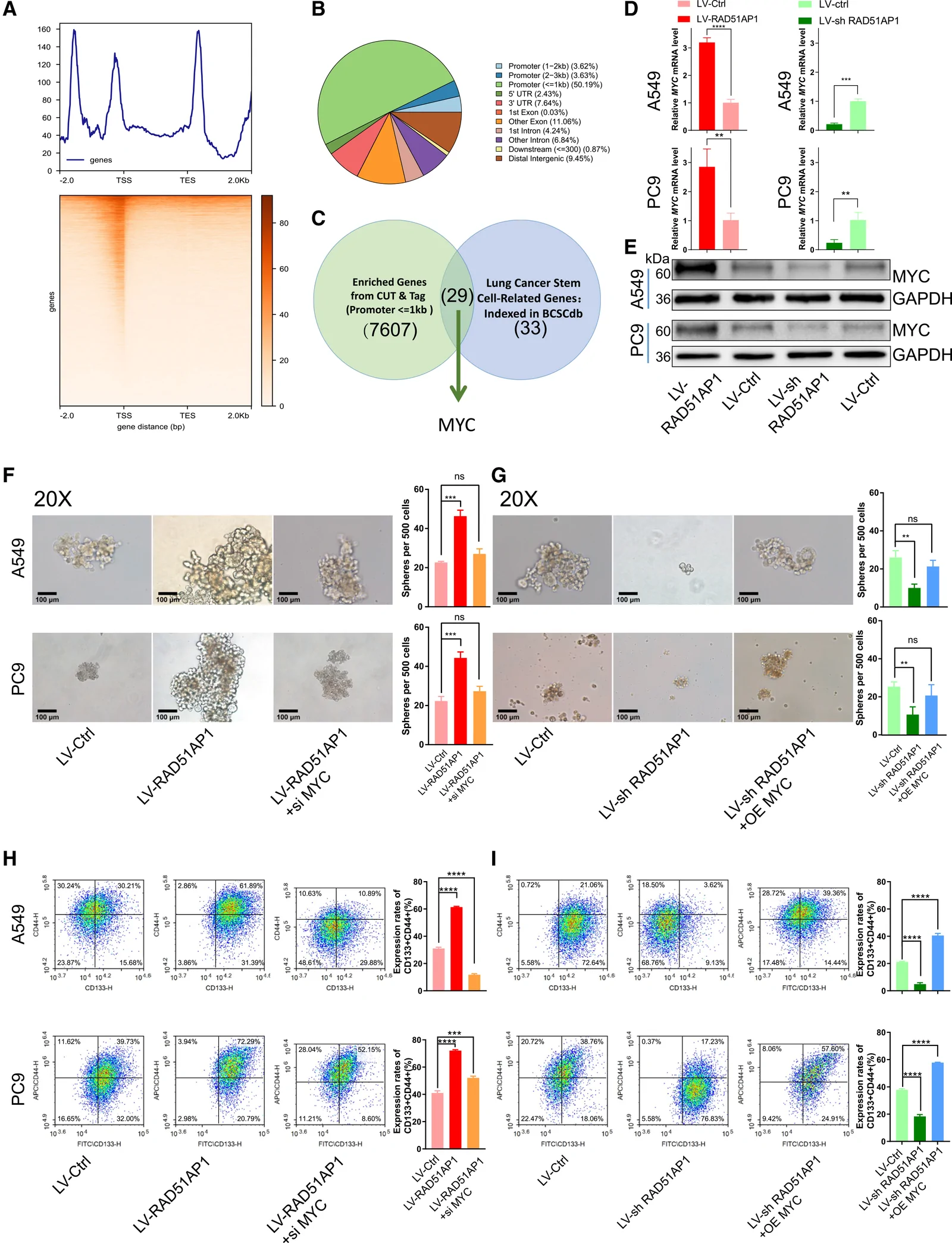
Screening and validation of the downstream regulatory mechanisms of RAD51AP1 (A and B) Cleavage under targets and tagmentation (CUT&Tag) analysis: DNA sequences enriched by RAD51AP1 were detected (CUT&Tag peak annotations in Data S1). Results showed that enriched gene peaks were mainly concentrated at the transcription start site (TSS) and the 2-kb region upstream of the TSS (A), with the highest enrichment in promoter ≤1 kb (50.19%) (B; marked in lime green). (C) Overlapping these peaks with lung cancer stem cell-related genes from BCSCdb (http://dibresources.jcbose.ac.in/ssaha4/bcscdb/index.php)^30^ revealed that the nuclear protein RAD51AP1 may directly bind to the *MYC* promoter region. Lung cancer stem cell biomarkers (related genes) downloaded from the BCSCdb are provided in Table S1. (D and E) qPCR (D) and WB (E) showed that RAD51AP1 increased MYC mRNA and protein expression in both A549 and PC9 cells (*n* = 3). (F) MYC knockdown significantly reversed the RAD51AP1-induced increase in SFE and sphere size in A549 and PC9 cells (*n* = 3). Scale bars, 100 μm. (G) MYC overexpression significantly rescued RAD51AP1 knockdown-induced decrease in SFE and sphere size in these cells (*n* = 3). Scale bars, 100 μm. (H) Flow cytometry showed that MYC knockdown significantly reversed the RAD51AP1-induced increase in the proportion of CD44^+^CD133^+^ subsets in both A549 and PC9 cells (*n* = 3). (I) Overexpressing MYC in LV-shRAD51AP1 A549 and PC9 cells rescued the reduced proportion of CD44^+^CD133+ subsets (*n* = 3). Data in (D) and (F)–(I) are represented as mean ± SD. Statistical significance was assessed using Student’s *t* test, ns: *p* ≥ 0.05; ∗∗: *p* < 0.01; ∗∗∗: *p* < 0.001; ∗∗∗∗: *p* < 0.0001.

Given that RAD51AP1 is a primary nuclear protein and is broadly involved in HR,^8^ we used cleavage under targets and tagmentation (CUT&Tag) to identify its potential binding to the *MYC* promoter (see Figures 4A–4C for screening details). qPCR and WB showed that RAD51AP1 significantly upregulates MYC mRNA (Figure 4D) and protein (Figure 4E) in lung cancer cells. Furthermore, MYC knockdown reversed RAD51AP1 overexpression-induced enhancement of sphere-forming capacity in A549 and PC9 cells (Figure 4F). Conversely, MYC overexpression rescued RAD51AP1 knockdown-induced stemness inhibition (Figure 4G). Meanwhile, flow cytometry showed that MYC knockdown significantly reversed the RAD51AP1-induced increase in the proportion of CD44^+^CD133^+^ subsets, while MYC overexpression restored the reduced CD44^+^CD133^+^ population caused by shRAD51AP1 (Figures 4H and 4I).

### RAD51AP1 transcriptionally upregulates MYC by directly binding its promoter region

**Figure 5:**
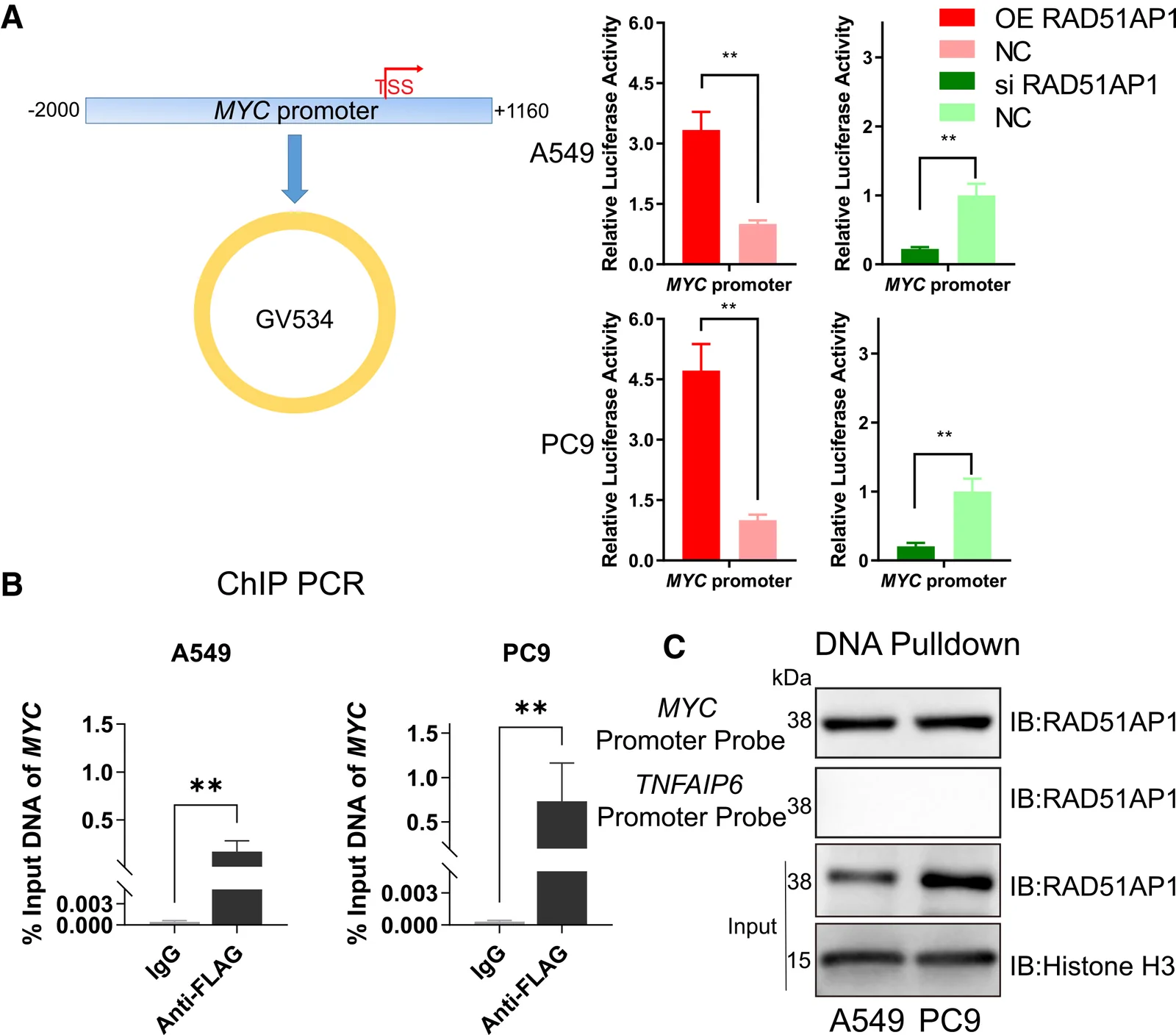
RAD51AP1 directly bound to the *MYC* promoter region to transcriptionally upregulate *MYC* (A) Dual-luciferase reporter assay: a GV534 luciferase reporter vector containing the *MYC* promoter (−2000 to +1160) was constructed (left image). Results showed that RAD51AP1 significantly increased the relative luciferase activity in both A549 and PC9 cells (right image, *n* = 6). (B) ChIP-qPCR demonstrating that enrichment of the *MYC* promoter region in RAD51AP1 immunoprecipitates (anti-FLAG was used, as no commercial ChIP-grade antibody against RAD51AP1 was available during the study) was significantly higher than that in IgG (*n* = 6). Primers used for the *MYC* promoter region are listed in Table 3. (C) DNA pull-down-WB revealed that the *MYC* promoter probe captured RAD51AP1 in both A549 and PC9 cells. The *TNFAIP6* promoter probe served as a negative control. The primers used for the probe construction are listed in Table 3. Data in (A) and (B) are represented as mean ± SD. Statistical significance was assessed using the Mann-Whitney U test. ∗∗: *p* < 0.01.

A dual-luciferase reporter assay showed that RAD51AP1 significantly increased the transcriptional activity of the *MYC* promoter plasmid (Figure 5A). We further designed *MYC* promoter primers (Table 3) and performed chromatin immunoprecipitation (ChIP)-qPCR assays using A549 and PC9 cells stably transfected with LV-RAD51AP1(3×FLAG-tagged). The results revealed significant enrichment of the *MYC* promoter by RAD51AP1 (Figure 5B). For DNA pull-down-WB, we constructed a *MYC* promoter probe with primers (Table 3), which demonstrated the specific binding of the probe to RAD51AP1 (Figure 5C).

**Table 3:** Primer sequences used in this study

Primer name	Sequence (5′ to 3′)
ACTIN-F	TCGTGCGTGACATTAAGGAGAAGC
ACTIN-R	CAGGAAGGAAGGCTGGAAGAGTG
RAD51AP1-F	GTGCGGCCTGTGAGACATAA
RAD51AP1-R	GGCACTGCACTAGCTGGAATA
MYC-F	TACAACACCCGAGCAAGGAC
MYC-R	CTAACGTTGAGGGGCATCGT
Sam68-F	ACAAGTACCTGCCCGAACTC
Sam68-R	TTCCCCACAAAATTGAACTTGGG
RAD51AP1 −2000/+50 F (biotin-labeled)	biotin-AAATCAGTTCTTACATTATTTTCTACACTG
RAD51AP1 −2000/+50 F (unlabeled)	AAATCAGTTCTTACATTATTTTCTACACTG
RAD51AP1 −2000/+50 R	GGTCCCTTTCAAGGCTTGG
MYC −2000/+1160 F	biotin-AAATTAACCGGGTGTGGTGGTGCACTGCAC
MYC −2000/+1160 R	CGTCTAAGCAGCTGCAAGGAGAGCCTTTCA
Sam68 −2000/+128 F	biotin-TAATTTTTGTATTTTTAGTAGAGACGGGGT
Sam68 −2000/+128 R	CTGGGAGGATGTGCGATCCAAGGAGCGAGA
TNFAIP6 −2000/+75 F	biotin-AACCCCATTTCCTCTTTCC
TNFAIP6 −2000/+75 R	ATCGTCAGTTGTAGTGAAG
RAD51AP1 −2000/+50 F (ChIP)	AGCTGACTTGCCGACATACTT
RAD51AP1 −2000/+50 R (ChIP)	GACATAGGGGCACTTGGACT
MYC −2000/+1160 F (ChIP)	CAGGCAGACACATCTCAGGG
MYC −2000/+1160 R (ChIP)	GCTGCCAAAGCAGCAGATAC
Sam68 −2000/+128 F (ChIP)	GTCACGTGGAAAACGAAACG
Sam68 −2000/+128 R (ChIP)	GAAGTCGCCTCCCGACTC

### Sam68 transcriptionally upregulates RAD51AP1 via direct binding to its promoter

**Figure 6:**
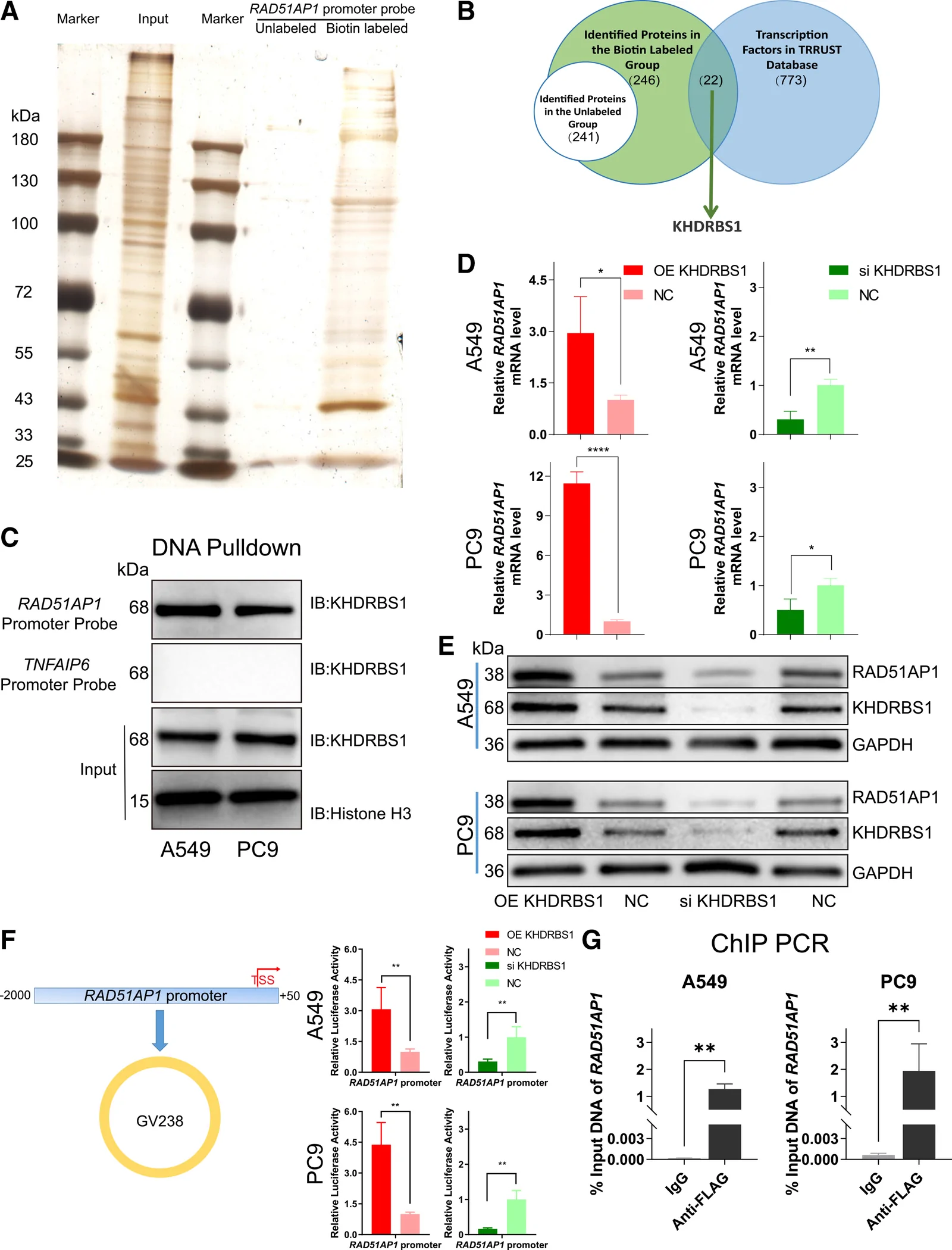
Sam68 transcriptionally upregulated *RAD51AP1* by directly binding to its promoter (A) Silver staining of DNA pull-down assays: biotin-labeled and unlabeled primers (listed in Table 3) were designed to construct the *RAD51AP1* promoter probe, and DNA pull-down products were subjected to silver staining for comparative analysis. (B) Schematic for Sam68 screening: DNA pull-down products were analyzed using liquid chromatography-mass spectrometry (LC-MS) (unlabeled/labeled products in Data S2 and S3). Proteins in the biotin-labeled group (green circle) were filtered to exclude those in the unlabeled group (blank circle) and then overlapped with 795 transcription factors (TFs) from the TRRUST database (cyan circle; https://www.grnpedia.org/trrust/).^31^ This screening identified Sam68 (KHDRBS1) as a potential *RAD51AP1*-regulating TF. (C) DNA pull-down-WB confirmed that the *RAD51AP1* promoter probe captured Sam68 in both A549 and PC9 cells. The *TNFAIP6* promoter probe served as a negative control. (D and E) qPCR and WB assays showed that Sam68 upregulated RAD51AP1 mRNA (D) and protein (E) expression in A549 and PC9 cells (*n* = 3). (F) Dual-luciferase reporter assay: *RAD51AP1* promoter (−2000 to +50) was cloned into the GV238 plasmid (left image). Relative luciferase activity was significantly higher in Sam68-overexpressing lung cancer cells and lower in Sam68-silenced cells (right image, *n* = 6). (G) ChIP-qPCR assay: Sam68 significantly enriched the *RAD51AP1* promoter in A549 and PC9 cells (*n* = 6). Anti-FLAG was used, as no commercial ChIP-grade Sam68 antibody was available and the overexpression plasmid carried a 3×FLAG tag. IgG was used as a negative control. The primers used are listed in Table 3. Data in (D), (F), and (G) are represented as mean ± SD. Statistical significance was assessed using Student’s *t* test for (D) and the Mann-Whitney U test for (F) and (G). ∗: *p* < 0.05; ∗∗: *p* < 0.01; ∗∗∗∗: *p* < 0.0001.

For upstream analyses, biotin-labeled and unlabeled probes (primers in Table 3) were constructed for DNA pull-down assays. Products were analyzed by silver staining (Figure 6A) and detected by liquid chromatography-mass spectrometry (LC-MS) (Data S2 and S3). We then removed non-specifically bound proteins from the biotin-labeled probe pull-down and overlapped the results with previously reported transcription factors. This screening identified Sam68 (also known as KHDRBS1: a heteronuclear ribonucleoprotein particle K homology [KH] single domain-containing, RNA-binding, signal transduction-associated protein 1) as a potential upstream transcriptional regulator of *RAD51AP1* (screening schematic in Figure 6B).

Subsequent experiments validated this regulatory relationship, showing that DNA pull-down-WB confirmed that Sam68 binds to the *RAD51AP1* promoter in A549 and PC9 cells (Figure 6C). Sam68 also upregulated RAD51AP1 mRNA (Figure 6D) and protein (Figure 6E) levels. The dual-luciferase reporter assay showed that Sam68 significantly increased *RAD51AP1* transcriptional activity in A549 and PC9 cells (Figure 6F), whereas ChIP-qPCR (primers in Table 3) further revealed its significant enrichment at the *RAD51AP1* promoter (Figure 6G).

### MYC positively upregulates Sam68 via direct promoter binding, closing the feedback loop

**Figure 7:**
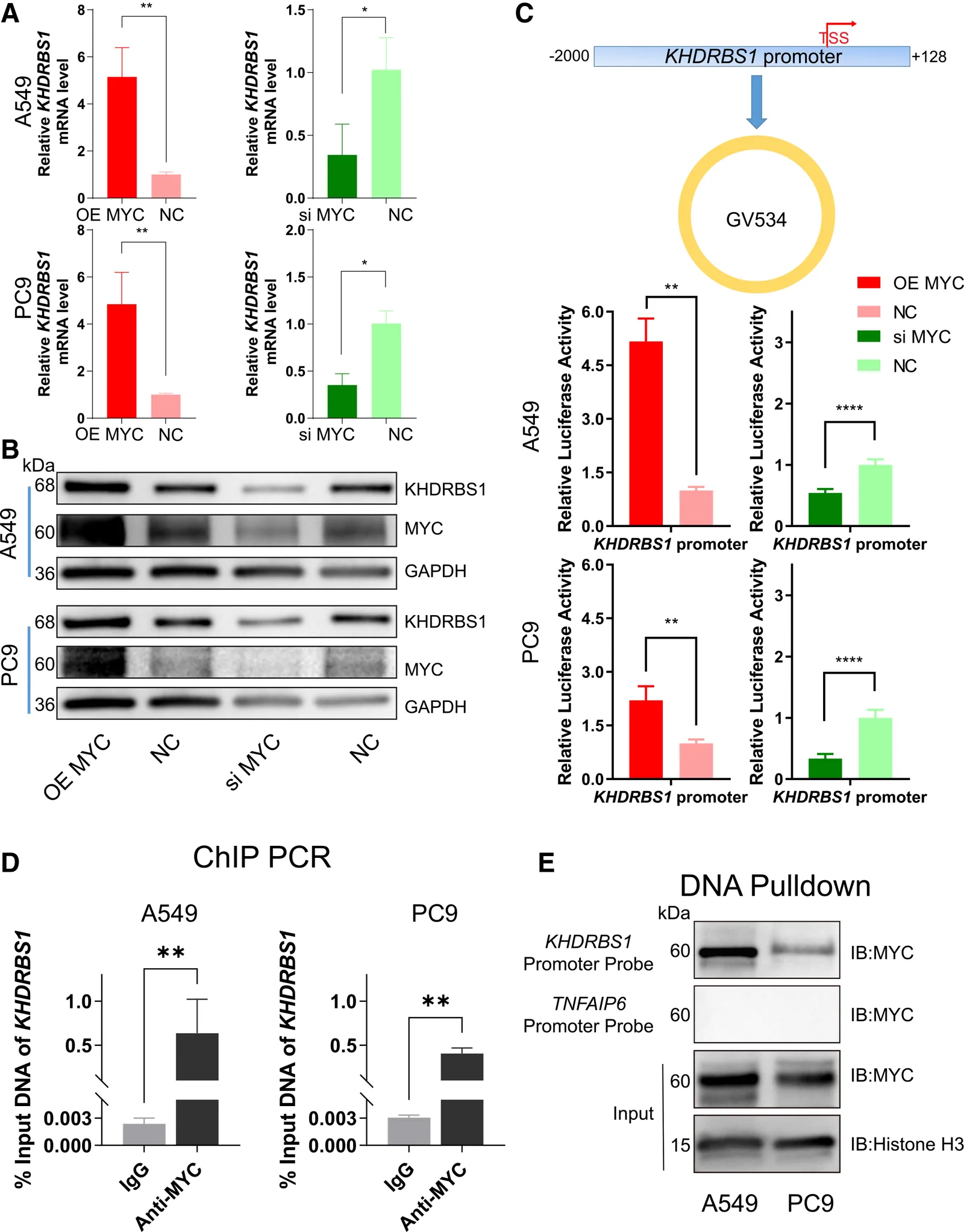
MYC fed back to bind the *Sam68* promoter and upregulated its expression (A and B) MYC may feedback to upregulate *Sam68* in lung cancer, as its transcriptional upregulation of *Sam68* in breast cancer has been reported by Turdo et al.^32^ Our results showed that MYC indeed promoted Sam68 mRNA (A) and protein (B) expression in both A549 and PC9 cells (*n* = 3). (C) Dual-luciferase reporter assays: The *Sam68* promoter (−2000 to +128) was cloned into the GV534 plasmid (to minimize the potential effects of MYC on GV238), as shown in the upper image. The results demonstrated that MYC significantly increased *Sam68* promoter-driven relative luciferase activity (lower image, *n* = 6). (D) ChIP-qPCR assays revealed that MYC significantly increased the percentage of input DNA at the *Sam68* promoter (*n* = 6). Primers used in this assay are listed in Table 3. (E) DNA pull-down assays further confirmed that the *Sam68* promoter probe binds MYC in A549 and PC9 cells. The probe-construction primers used are listed in Table 3. Data in (A), (C), and (D) are represented as mean ± SD. Statistical significance was assessed using Student’s *t* test for (A) and for the siMYC versus NC comparisons in (C), and the Mann-Whitney U test for the MYC overexpression versus NC comparisons in (C) and for (D). ∗: *p* < 0.05; ∗∗: *p* < 0.01; ∗∗∗∗: *p* < 0.0001.

Interestingly, MYC has been reported to significantly transcriptionally upregulate *Sam68* in breast cancer.^32^ To determine whether MYC exerts a similar regulatory function in lung cancer, we performed qPCR and WB analyses, which showed that MYC significantly upregulated Sam68 mRNA (Figure 7A) and protein (Figure 7B) in lung cancer cells. Additionally, a dual-luciferase reporter assay revealed that MYC significantly increased *Sam68* transcriptional activity (Figure 7C). Both ChIP-qPCR and DNA pull-down assays confirmed that MYC binds significantly to the *Sam68* promoter (Figures 7D and 7E).

**Figure 8:**
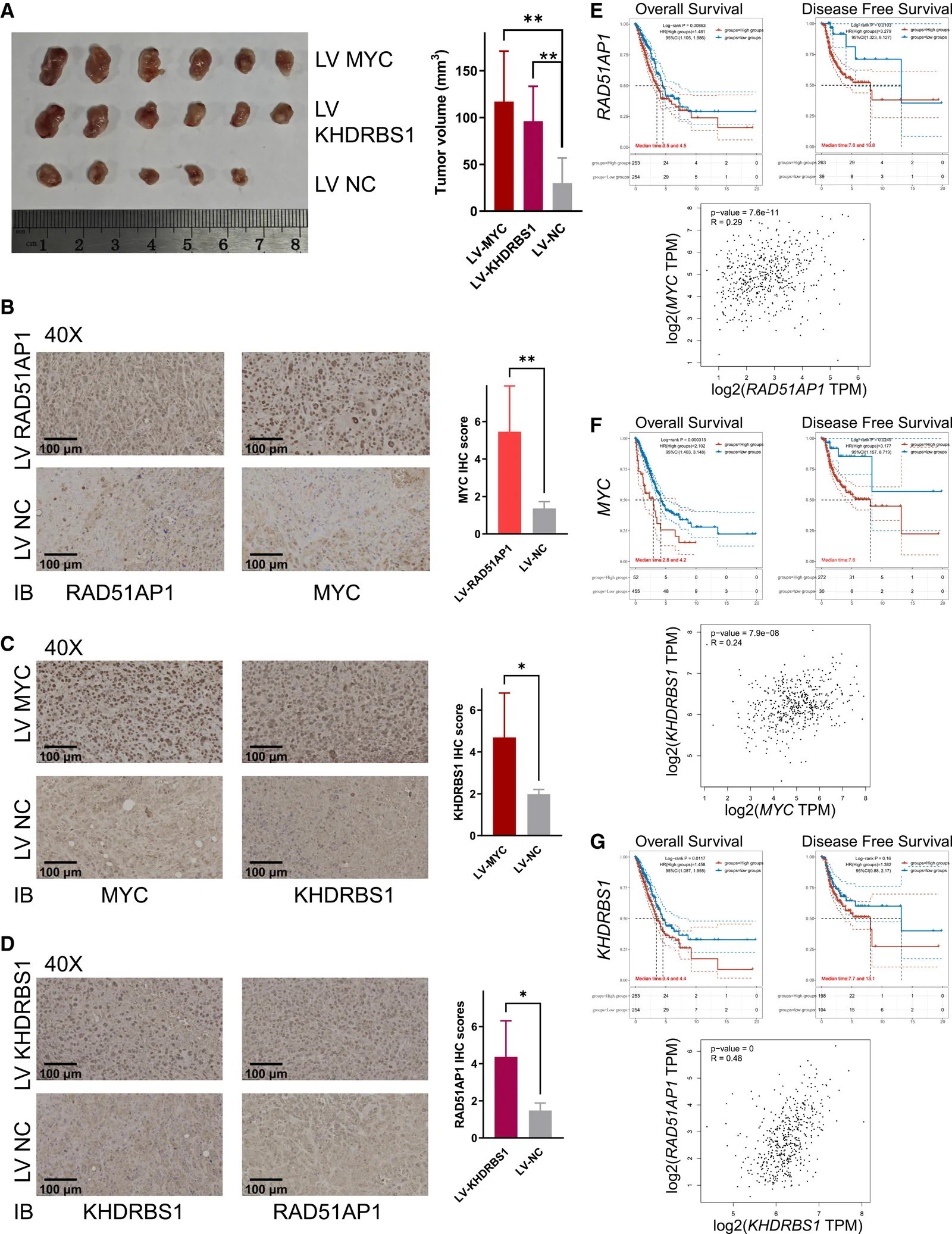
RAD51AP1-MYC-Sam68 feedback loop drives CDX growth and unfavorable LUAD prognosis (A) To validate the *in vivo* role of the RAD51AP1-MYC-Sam68 loop, we established CDX models using cells infected with MYC- or Sam68-overexpressing lentiviruses (cell density: 5 × 10^5^; *n* = 6 per group), following the protocol described in Figures 3A–3C. Results showed that both MYC and Sam68 overexpression led to significantly larger tumors than in the control group (*p* < 0.01). (B) Immunohistochemical (IHC) staining was performed to detect MYC expression in the CDX models (5 × 10^5^ cells) described in Figures 3A–3C. The results indicated that RAD51AP1 significantly promoted MYC expression. Scale bars, 100 μm. (C and D) Similarly, MYC and Sam68 also promoted the expression of Sam68 (C) and RAD51AP1 (D), respectively. Scale bars, 100 μm. (E–G) Bioinformatics analysis of the RAD51AP1-MYC-Sam68 loop in LUAD: survival data were extracted from The Cancer Genome Atlas (TCGA; https://portal.gdc.cancer.gov/) and analyzed using the survival and survminer packages in R software. Correlation analysis was performed via Gene Expression Profiling Interactive Analysis (GEPIA; http://gepia2.cancer-pku.cn/#index).^33^ In LUAD, high expression of *RAD51AP1* (E), *MYC* (F), and *Sam68* (G) was all associated with poor prognosis, and their expression levels were positively correlated with each other. Data in (A)–(D) are represented as mean ± SD. Statistical significance was assessed using Student’s *t* test for (A) and Welch’s *t* test for (B)–(D). ∗: *p* < 0.05; ∗∗: *p* < 0.01.

Furthermore, overexpression of MYC or Sam68 in A549 cells both promoted tumor growth of CDXs in nude mice (Figure 8A). Immunohistochemical (IHC) staining showed that RAD51AP1 significantly upregulated MYC expression, MYC promoted Sam68 expression, and Sam68 enhanced RAD51AP1 expression, forming a positive feedback loop *in vivo* (Figures 8B–8D). Bioinformatics analysis showed that high *RAD51AP1* expression was associated with worse OS (*p* = 0.0086) and disease-free survival (DFS) (*p* = 0.0103) in LUAD patients (Figure 8E). Additionally, *RAD51AP1* expression was positively correlated with *MYC* in LUAD (Figure 8E). High expression of *MYC* and *Sam68* also predicted worse prognosis, with *MYC* positively correlated with *Sam68* (Figure 8F) and *Sam68* positively correlated with *RAD51AP1* (Figure 8G).

## Discussion

We found that RAD51AP1 significantly promoted cancer stemness in lung cancer both *in vitro* and *in vivo* (Figures 2 and 3). It also enhanced lung cancer cell proliferation and migration, and was associated with poor prognosis (Figures 1 and 3). Mechanistically, RAD51AP1 maintained lung cancer stemness primarily through MYC upregulation (Figure 4). Specifically, it directly bound to the *MYC* promoter region to transcriptionally upregulate MYC expression (Figure 5). Upstream, our screening and validation showed that Sam68 directly bound to the *RAD51AP1* promoter to promote its transcription (Figure 6). Interestingly, MYC also directly bound to the *Sam68* promoter and transcriptionally upregulated its expression (Figure 7). Furthermore, RAD51AP1, MYC, and Sam68 all promoted CDX tumorigenesis and each other’s expression, while bioinformatics analysis showed that their high expression predicted poor prognosis and their expressions were positively correlated with each other(Figures 3A–C and 8). These results suggest that a RAD51AP1-MYC-Sam68-RAD51AP1 positive feedback loop stably maintains lung cancer stemness. Notably, this feedback loop operated at the transcriptional level, as shown in the graphical abstract (created using BioGDP.com^34^).

As previously reported, RAD51AP1 promotes tumor proliferation and stemness in multiple cancers.^15^^,^^16^ Consistent with these findings, our study confirmed that RAD51AP1 sustains stemness in lung cancer. However, the mechanism by which RAD51AP1 maintains cancer stemness remains unclear. Our data show that RAD51AP1 directly binds to the *MYC* promoter region to transcriptionally upregulate *MYC* (Figure 5). MYC is essential for maintaining cancer stemness, including lung cancer.^35^ Thus, we conclude that RAD51AP1 maintains lung cancer stemness through the transcriptional upregulation of *MYC*. This observation of their direct interactions may provide a working model for cancer stemness maintenance.

Importantly, this part of our study had a limitation: we did not identify the specific binding site of RAD51AP1 in the *MYC* promoter region. The 1,160 bp distance between *MYC*’s transcription start site (TSS) and coding sequence (CDS)—a segment that has also been included in the luciferase plasmid (Figure 5A)—raises the possibility that RAD51AP1 binds to a regulatory region rather than the traditional promoter. However, without identifying the exact binding sites, this remains unclear. Therefore, defining RAD51AP1 as a conventional transcription factor requires extreme caution.

We also demonstrated that Sam68 directly binds to the *RAD51AP1* promoter to promote its transcription (Figure 6). Sam68 (encoded by *KHDRBS1*) is a multifunctional protein that regulates gene expression via RNA binding, mediates signal transduction, and participates in processes including the cell cycle, tumorigenesis, and DNA damage repair.^36^^,^^37^ It is also shown to be involved in the self-renewal of CSCs in breast and colorectal cancers.^23^^,^^24^^,^^25^ In lung cancer, Sam68 is significantly overexpressed compared to normal tissues and is associated with a poor prognosis.^26^ However, their relationship with lung cancer stemness has rarely been reported. Our demonstration of the direct Sam68-*RAD51AP1* promoter interaction and its role in transcriptional upregulation explains how Sam68 promotes stemness in lung cancer, along with the underlying mechanism.

However, our study did not clarify the specific mechanism by which Sam68 acts on the *RAD51AP1* promoter. Sam68 contains proline-rich sequences and tyrosine-rich regions, which enable it to bind to signaling molecules with domains such as SH3, WW, and SH2, thereby participating in various biological processes including transcriptional regulation (as reviewed by Frisone et al.).^38^ This suggests that Sam68 may also recruit other molecules via these structural features to upregulate RAD51AP1 expression. Such fine regulatory mechanisms were not explored in this part of our study, and this limitation warrants further investigation in future research.

Notably, building on our finding that MYC transcriptionally upregulates *Sam68* (Figure 7), we demonstrated a regulatory model: the RAD51AP1-MYC-Sam68-RAD51AP1 positive feedback loop, which promotes lung cancer stemness maintenance. While positive feedback loops are common in cancer stemness regulation (e.g., Tan et al. identified a WNT2-SOX4 loop in gastric cancer^39^), and MYC-mediated transcriptional upregulation of *Sam68* itself is not uncommon (as reported in breast cancer^32^), such direct transcriptional cross-regulation between three proteins—RAD51AP1, MYC, and Sam68—forming a positive feedback loop remains rarely observed in lung cancer. Our characterization of this feedback loop—wherein the three proteins interact with their downstream promoters—may provide a theoretical basis for targeting lung cancer stemness-sustaining processes. Although no drugs targeting RAD51AP1, MYC, and/or Sam68 have been approved for clinical use in lung cancer to date, developing agents, including small-molecule inhibitors and/or neutralizing antibodies that disrupt this positive feedback loop, may offer therapeutic promise by suppressing cancer stemness and improving clinical outcomes.

### Limitations of the study

Our study revealed a RAD51AP1-MYC-Sam68-RAD51AP1 positive feedback loop that drives lung cancer stemness maintenance. That said, we have not yet clarified which event triggers this positive feedback loop in our study. Both RAD51AP1 and Sam68 are closely linked to DNA damage repair. Whether the high replicative stress in tumors induces overexpression of RAD51AP1 and/or Sam68—thereby initiating this feedback loop—remains to be further investigated. Additionally, how to terminate this positive feedback regulation represents another critical direction for future exploration.

Meanwhile, this study focused only on transcriptional regulation among these molecules. Whether they are regulated at other levels, including translational and/or post-translational levels, remains unclear and requires further investigation. Furthermore, as our work is primarily mechanistic, its clinical significance and translational potential need to be further validated in future clinical studies.

## Resource availability

### Lead contact

Requests for further information and resources should be directed to and will be fulfilled by the lead contact, Jun Chen (huntercj2004@qq.com).

### Materials availability

All reagents generated in this study are available from the lead contact with a completed materials transfer agreement.

### Data and code availability

• All data reported in this paper will be shared by the lead contact upon request.
• This paper does not report original code.
• Any additional information required to reanalyze the data reported in this paper is available from the lead contact upon request.

## Acknowledgments

This work was supported by the 10.13039/501100001809National Natural Science Foundation of China (grant nos. 82303537 and 82473191). We are grateful to Dr. Dan Wang and Dr. Tao Shi for valuable technical support and insightful discussions throughout this study.

## Author contributions

Conceptualization, R.L. and J.C.; methodology, M.L., J.W., and Z.H.; investigation, R.L., M.L., Z.Z., S.Y., and Z.H.; writing – original draft, R.L., G.Z., and Z.H.; writing – review and editing, R.L. and J.C.; funding acquisition, R.L. and J.C.; resources, P.C. and H.F.; supervision, J.C.

## Declaration of interests

The authors declare no competing interests.

## Declaration of generative AI and AI-assisted technologies in the writing process

During the preparation of this work, the authors used DeepSeek in order to assist with language polishing and grammar correction. After using this tool or service, the authors reviewed and edited the content as needed and take full responsibility for the content of the publication.

## STAR★METHODS

### Key resources table

**Table:** 

REAGENT or RESOURCE	SOURCE	IDENTIFIER
**Antibodies**		

RAD51AP1	Proteintech	11255-1-AP; RRID: AB_2300786
MYC	CST	5605S; RRID: AB_1903938
Sam68	Proteintech	10222-1-AP; RRID: AB_2130292
GAPDH	Proteintech	60004-1-Ig; RRID: AB_2107436
histone H3	Proteintech	17168-1-AP; RRID: AB_2716755

**Biological samples**		

FFPE from lung cancer patients	Tianjin Medical University	N/A

**Chemicals, peptides, and recombinant proteins**		

TRIzol Reagent	Invitrogen	15596026CN
Lipofectamine 2000	Invitrogen	11668500
Puromycin	Sigma-Aldrich	P9620
Crystal Violet	Sigma-Aldrich	32675
Paraformaldehyde (4%)	Sigma-Aldrich	P6148
Triton X-100	Sigma-Aldrich	93443
DAPI mounting medium	Beyotime Biotechnology	P0131
Epidermal Growth Factor (EGF)	Gibco	PHG0314
Basic Fibroblast Growth Factor (bFGF)	ACRO Biosystems	BFF-H4117
B27 Supplement	Beyotime Biotechnology	C0347
Streptavidin Magnetic Beads	Beyotime Biotechnology	P2151

**Critical commercial assays**		

Cell Counting Kit-8 (CCK-8)	APExBIO	K1018
Power SYBR Green PCR Master Mix	Applied Biosystems	4367659
Dual-Luciferase Reporter Gene Assay Kit	Beyotime Biotechnology	RG027
BeyoChIP™ ChIP Assay Kit	Beyotime Biotechnology	P2080S
Gel DNA Extraction Kit	TaKaRa	9762
Fast Silver Stain Kit	Beyotime Biotechnology	P0017S

**Experimental models: Cell lines**		

A549	ATCC	CRM-CCL-185
PC9	Procell	CL-0668

**Experimental models: Organisms/strains**		

BALB/c nude mice (CAnN.Cg-*Foxn1*^*nu*^/Crl)	Charles River	401

**Oligonucleotides**		

Primers	This study	Table 3

**Recombinant DNA**		

RAD51AP1 overexpression plasmid	This study	N/A
Sam68 overexpression plasmid	This study	N/A
MYC overexpression plasmid	This study	N/A
MYC promoter luciferase reporter plasmid	This study	N/A
RAD51AP1 promoter luciferase reporter plasmid	This study	N/A
Sam68 promoter luciferase reporter plasmid	This study	N/A
Renilla luciferase control plasmid	This study	N/A

**Software and algorithms**		

R software	R Project	v4.0.3
NovoExpress	Agilent Technologies	v1.5.0
Proteome Discoverer	Thermo Scientific	v2.5
SPSS	IBM	v23
fastp	OpenGene	v0.20.0
Bowtie 2	Johns Hopkins University	v2.3.4.3
SEACR	Fred Hutchinson Cancer Center	v1.3
IGV	Broad Institute	v2.17.4
ChIPseeker	Bioconductor	v1.12.1

### Experimental model and study participant details

#### Cell culture

Human lung adenocarcinoma A549 and PC9 cell lines were used in this study. The A549 cell line was purchased from the American Type Culture Collection (ATCC; CRM-CCL-185), and PC9 was purchased from Procell (Wuhan, China; CL-0668). All cells were cultured in RPMI 1640 medium (Gibco, USA) supplemented with 10% fetal bovine serum (FBS) and 1% penicillin-streptomycin, and were maintained in a humidified incubator at 37 °C with 5% CO_2_. All cell lines were authenticated by short tandem repeat (STR) profiling. Mycoplasma contamination was routinely tested and confirmed negative.

#### Animal experiments

Four-to five-week-old female BALB/c nude mice (CAnN.Cg-*Foxn1*^*nu*^/Crl; strain code 401) were used to establish the CDX model. Mice were maintained under specific pathogen-free (SPF) conditions with free access to food and water. 5×10^3^, 5×10^4^, and 5×10^5^ overexpressed RAD51AP1 or NC A549 cells were injected into the groins of mice. Each group consisted of six mice. Tumor size was measured and calculated every 7 days at 3 weeks after injection. For the validation of tumorigenesis mediated by MYC and KHDRBS1, the cell number used was 5×10^5^. The mice were euthanized, and tumor incidence was recorded five weeks after inoculation. The studies involving animals and their data or biological material were approved by the Laboratory Animal Welfare and Ethics Committee of Tianjin Medical University General Hospital (IRB2023-DWFL-103). As the *in vivo* experiments were performed exclusively in female mice, potential sex-related differences were not evaluated. This represents a limitation of the animal experiments.

#### Specimens

All 46 paraffin-embedded lung cancer tissues with complete clinical data were collected from the Tianjin Medical University General Hospital between January 2011 and December 2012. All participants were Asian. Based on the median RAD51AP1 fluorescence intensity (2.5982), the 46 patients were stratified into RAD51AP1 high-expression (*n* = 23) and low-expression (*n* = 23) groups. The patients were 32–75 years old. The baseline characteristics of the patients, including sex, smoking status, and histology, are detailed in Table 1. No statistically significant differences in these baseline characteristics were observed between the RAD51AP1 high-expression and low-expression groups (Table 2). Sex distribution did not differ significantly between the RAD51AP1 high- and low-expression groups (*p* = 0.376). However, the limited cohort size (*n* = 46) precluded adequately powered sex-stratified analyses, representing a limitation of this study. This study was performed in compliance with the CIOMS/WHO International Ethical Guidelines for Biomedical Research Involving Human Subjects (2002). The studies involving human participants and their clinical data were approved by the Medical Ethics Committee of Tianjin Medical University General Hospital (IRB2023-KY-074).

### Method details

#### Transfection and lentivirus infection

Lenti-RAD51AP1, Lenti-sh RAD51AP1, Lenti-MYC, and Lenti-Sam68 were provided by GeneChem (Shanghai, China) with their GV492 and GV493 vectors, respectively. si-RAD51AP1 (siG000010635B-1–5), si-MYC (SIGS0003660-1), and si-Sam68 (siG000010657A-1–5) were purchased from RiboBio (Guangzhou, China). The overexpressed RAD51AP1 (with 3XFLAG), Sam68 (with 3XFLAG), and MYC plasmids were produced by GeneChem (Shanghai, China).

The plasmids were transfected into cells using Lipofectamine 2000 (Invitrogen, USA). The siRNAs or lentiviral vectors were transfected or infected into cells according to the manufacturer’s instructions. Stable cell lines were established by monoclonal screening with puromycin after lentiviral infection.

#### Cell counting Kit-8 assay and colony formation assay

Cell proliferation was assessed as reported previously.^19^ Briefly, 4000 cells were seeded in each well and incubated for 24h, 48h, and 72h. Then, 10 μL CCK8 (APExBIO, United States) was added to each well. After 1h incubation, the OD value was measured at 450 nm. For the colony formation assay, 1000 cells were seeded in each well in 6-well plates. After 10–14 days of incubation, cells were fixed with 4% paraformaldehyde for 15 min and stained with 0.1% crystal violet for 30 min. Colonies were imaged and counted.

#### Wound-healing and transwell migration assay

*In vitro* migration assays were performed as previously described.^40^ In the wound-healing assay, a 1 mL sterile pipette tip was used to create a wound after the cells reached confluence. The floating cells were then washed with serum-free medium, and wound closure was observed at 0h, 24h, and 48h under an inverted microscope. The wound area at each time point was normalized and analyzed across experimental triplicates.

For the transwell migration assay, 5 × 10^4^ cells in serum-free medium were seeded in the upper transwell chambers (Corning, USA). After 24h of incubation, the cells migrated to the underside and were fixed with 4% paraformaldehyde and stained with 0.1% crystal violet. The experiment was performed in triplicate and the visual fields were imaged and counted under a microscope.

#### Quantitative real-time PCR

Quantitative real-time PCR (qRT-PCR) was performed as previously described.^40^ Total RNA was extracted using the TRIzol Reagent (Invitrogen, USA). Then, 2 μg RNA was reverse transcribed to cDNA, and the reaction was performed with Power SYBR Green PCR Master Mix (Applied Biosystems, USA). β-actin was used as an internal control. The primer sequences used are listed in Table 3.

#### Western blot analysis

Western blotting was performed as described previously.^40^ Nuclear and cytoplasmic protein fractions were isolated according to the manufacturer’s instructions (Beyotime Biotechnology, China). The denatured protein extracts were electrophoresed on 10% SDS-PAGE and transferred to PVDF membranes (Merck Millipore, USA). Membranes were blocked and incubated with primary antibodies overnight at 4°C under gentle rocking. After incubation with the secondary antibody for 1h at room temperature, the bands were visualized and imaged. The Primary antibodies used were anti-RAD51AP1 (1:1000, Proteintech, China), anti-MYC (1:1000, CST, USA), anti-Sam68 (1:2000, Proteintech, China), anti-GAPDH (1:5000, Proteintech, China), and anti-histone H3 (1:2000, Proteintech, China).

#### Sphere formation assay

Cells were plated in 6-well Ultra-Low Attachment Plates (Corning, USA) and cultured in DMEM/F12 (Gibco, USA) containing 2% B27 (Beyotime Biotechnology, China), 20 ng/mL EGF (Gibco, USA), and 10 ng/mL bFGF (ACRO Biosystems, China). After two weeks of incubation, the primary spheres were imaged and counted under an inverted microscope. Subsequently, SFE was calculated. Afterward, 40 μm cell strainers (Corning, USA) were used to harvest the primary spheres. Single cells were dissociated using trypsin and seeded in 6-well Ultra-Low Attachment Plates for secondary sphere assays. We then repeated the above process to perform a third sphere formation assay.

#### Flow cytometric analysis

The sphere pellets were digested with trypsin-EDTA to dissociate spheroids into single-cell suspension. Cells were washed twice with cold PBS and centrifuged at 1800 rpm for 5 min. The cell pellets were resuspended in 500 μL PBS, and 100 μL of each suspension was incubated with 5 μL FITC-conjugated anti-human CD133 antibody (BioLegend, USA) and 5 μL APC-conjugated anti-human CD44 antibody (BioLegend, USA) for 30 min in the dark at 4 °C. After staining, cells were washed twice with PBS to remove unbound antibodies, then resuspended in 300 μL PBS. Samples were analyzed on the NovoCyte 2000R flow cytometer (Agilent Technologies), and fluorescence signals were recorded. Data processing and quantification of CD44^+^CD133^+^ subset proportions were performed using NovoExpress software (v1.5.0). All experiments were performed in triplicate (*n* = 3).

#### Immunofluorescence

All samples were subjected to immunofluorescence staining to detect RAD51AP1 expression under the following procedure. First, after being placed in an oven set at 60°C for 1 h, the sections were deparaffinized with xylene for 40 min and rehydrated with ethanol at concentrations ranging from 100% to 70%. Citrate buffer antigen retrieval solution was used for antigen retrieval. A 0.5% Triton-PBS solution was used for membrane permeabilization. The sections were then blocked with 5% Normal Goat Serum (NGS) solution for 1 h RAD51AP1 (1:200; Proteintech, China) was used as the primary antibody and incubated overnight at 4°C. The sections were then incubated with the fluorescent secondary antibody 488 (1:200; Proteintech, China) at room temperature for 1 h. DAPI anti-fading mounting medium was used to mount sections.

#### CUT&Tag

The CUT&Tag assay was performed using the HyperactiveTM *In-Situ* ChIP Library Prep Kit for Illumina (cat.no.TD903-TD904, Vazyme Biotech, China) according to the manufacturer’s instruction.^41^ Briefly, the prepared ConA beads were added to resuspended LV RAD51AP1 (with 3XFLAG) A549 cells and incubated at room temperature to bind the cells. After cell membrane permeation, primary anti-FLAG antibody (Proteintech, China), secondary antibody, and hyperactive pA-Tn5 transposase were incubated with the bead-bound cells. The Hyperactive pA-Tn5 transposase cleaved DNA fragments bound to the target protein and ligated P5/P7 adapters, after which the libraries were amplified by PCR with P5 and P7 primers and sequenced on the Illumina NovaSeq6000 platform, and 150bp paired-end reads were generated for the following analysis. The raw sequence data were first quality trimmed using the fastp^42^ software to obtain clean reads. The clean reads were then aligned to the reference genome using Bowtie 2^43^ and analyzed using SEACR software. Visualization of peak distribution was performed using IGV. The peaks were then annotated using the ChIPseeker^44^ software.

#### Dual-luciferase reporter assay

Transient transfection of plasmids or siRNA to overexpress or knock down RAD51AP1, MYC, and Sam68 was performed using dual-luciferase reporter assays. The *MYC* TSS upstream 2000bp to downstream 1160bp (Figure 5A), −2000 to +50 bp of *RAD51AP1* (Figure 6F), and −2000 to +128 bp of *Sam68* (Figure 7C) were selected as their respective DNA promoter regions. All luciferase reporter plasmids and Renilla plasmids were produced by Genechem (Shanghai, China) and co-transfected into cells for 48h. Luciferase activity was measured using a dual-luciferase reporter gene assay kit (Beyotime, China, #RG027) following the manufacturer’s instructions.

#### Bioinformatics analysis

All bioinformatics data were from The Cancer Genome Atlas (TCGA) (https://portal.gdc.cancer.gov/). Survival analyses with log rank tests were conducted using the survival and survminer packages in R software (v4.0.3). The open-access bioinformatics analysis tool GEPIA (http://gepia2.cancer-pku.cn/#index)^45^ was used for the correlation analyses. All parameters were set to default values.

#### DNA pulldown assay

Biotin-labeled and unlabeled DNA probes were PCR-amplified using the primers listed in Table 3. After amplification, the products were separated by 1% agarose gel electrophoresis. Target fragment bands were excised under UV light and purified using a TaKaRa Gel DNA Extraction Kit (Japan). For the DNA pulldown assay, the extracted DNA probes were incubated with pre-washed streptavidin magnetic beads (Beyotime Biotechnology, China) for 30 min at room temperature. After two washes, the nuclear extracts were incubated with bead-probe complexes at 4 °C for 4 h with rotation. Following elution and centrifugation, the supernatants were analyzed by WB.

#### IHC staining

The expression of RAD51AP1, MYC, and Sam68 in CDX tissues was detected by IHC staining as previously described.^46^ Briefly, tissue sections were deparaffinized, subjected to antigen retrieval in 5 mM Tris-HCl for 10 min via microwave pretreatment, and endogenous peroxidase activity was quenched with 3% H_2_O_2_. Non-specific binding sites were blocked with serum, followed by overnight incubation at 4°C with primary antibodies against RAD51AP1, MYC, or Sam68 (all 1:200, Proteintech, China). After washing, sections were incubated with horseradish peroxidase (HRP)-conjugated secondary antibody for 30 min, stained with diaminobenzidine (DAB), and counterstained with hematoxylin. Five random fields per section were selected, and two pathologists independently quantified the relative protein expression levels.

#### Silver staining and LC-MS

Silver staining of *RAD51AP1* promoter probe pulldown products was performed using a Fast Silver Stain Kit (Beyotime, China) following the manufacturer’s instructions. Nuclear extracts served as the input control, and unlabeled probe pulldown products were used as the negative controls. The protein composition of biotinylated DNA probe pulldown products was analyzed using LC-MS at Lumingbio (Shanghai, China). Briefly, the protein samples were digested, desalted, and separated using an EASY-nLC 1200 Nano-HPLC system (Thermo Scientific, USA). Detection was performed using a Fusion mass spectrometer (Thermo Scientific, USA) in data-dependent acquisition mode, followed by database searching using Proteome Discoverer 2.5 software.

#### ChIP qPCR

ChIP assays were performed using the BeyoChIP ChIP Assay Kit (Beyotime, China) following the manufacturer’s protocol. Briefly, cells were cross-linked with formaldehyde for 10 min, lysed on ice, and sonicated to shear the genomic DNA. Anti-MYC antibody (CST, USA), anti-FLAG antibody (Proteintech, China), or IgG was added, followed by overnight rotation at 4 °C. After adding 80 μL of Protein A/G Magnetic Beads/Salmon Sperm DNA, the mixture was rotated at 4 °C for 1 h. The bead-bound complexes were then washed sequentially five times with the corresponding buffers. Following elution, the supernatant was collected, supplemented with 20 μL of 5M NaCl, and heated at 65 °C for 4 h to reverse the cross-linking. Finally, after DNA purification, the products were detected by qPCR using the primers listed in Table 3.

### Quantification and statistical analysis

The quantification of protein expression by IHC staining is described in detail in the IHC staining section of the Methods. The median value of immunofluorescence intensity was used as the cutoff to stratify patients into RAD51AP1 high-expression and low-expression groups. SPSS version 23 (IBM, USA) was used for the statistical analysis. Statistical tests used for individual comparisons, including Student’s *t* test, Welch’s *t* test, Mann-Whitney U test, and two-way ANOVA, are specified in the corresponding figure legends. The Kaplan–Meier method and log rank test were used to plot survival curves and compare the OS and PFS. Statistical significance is indicated by asterisks as ∗*p* < 0.05; ∗∗*p* < 0.01; ∗∗∗*p* < 0.001; ∗∗∗∗*p* < 0.0001.

## Supplemental information

Document S1. Table S1 and Data S4

Data S1. CUT & TAG peak annotations

Data S2. DNA pull-down MS for unlabeled *RAD51AP1* promoter probe

Data S3. DNA pull-down MS for labeled *RAD51AP1* promoter probe

Data S5. Ethics approvals for animal and human studies
