# Supplementary material for: RAD51AP1-MYC-Sam68 positive transcriptional feedback loop maintains lung cancer stemness
Source: iScience. 2026 Aug 31;29(9):117397. doi: 10.1016/j.isci.2026.117397 (PMC13562426; doi:10.1016/j.isci.2026.117397)
Supplement: Supplementary file 1 [file mmc1.pdf]

iScience, Volume *29*

Supplemental information

RAD51AP1-MYC-Sam68 positive transcriptional feedback loop maintains lung cancer stemness

Renwang Liu, Mingbiao Li, Zixuan Hu, Shen Yang, Guangsheng Zhu, Peijun Cao, Haiyang Fan, Jianfang Wang, Zitong Zhang, and Jun Chen

Table S1. The biomarkers of lung cancer stem cells download from Biomarkers of Cancer Stem Cells Database (BCSCdb)

Gene Symbols	HGNC_ID
NANOG	20857
POU5F1	9221
SOX2	11195
ALDH1A1	402
ALCAM	400
CD44	1681
ABCG2	74
FSCN1	11148
NES	7756
PROM1	9454
SCD	10571
SH2D3A	16885
LGR5	4504
ABCB1	40
CD24	1645
MET	7029
EPCAM	11529
CTNNB1	2514
LIN28A	15986
ABCG5	13886
BHLHA15	22265
CAV1	1527
DRD2	3023
FZD7	4045
GLI1	4317
KDM1A	29079
KIT	6342
MSI1	7330
MYC	7553
NOTCH1	7881
REL	9954
RELA	9955
RELB	9956
THY1	11801
BMI1	1066
CCND1	1582
CDKN1A	1784
DLL4	2910
EZH2	3527
GLDC	4313
GSK3B	4617

ITGA6	6142
JAG1	6188
JUNB	6205
KLF4	6348
LIN28B	32207
MDM2	6973
MIRLET7C	31480
MIRLET7F1	31483
MIRLET7G	31485
MMP9	7176
NOTCH3	7883
PTEN	9588
SNAI1	11128
SUZ12	17101
TERT	11730
TGFB1	11766
CD274	17635
ITGB1	6153
ITGB4	6158
LGR6	19719
PLAUR	9053

Abbreviation： HGNC_ID, HUGO Gene Nomenclature Committee ID.

Data S4: Original WB Images

Figure 1A

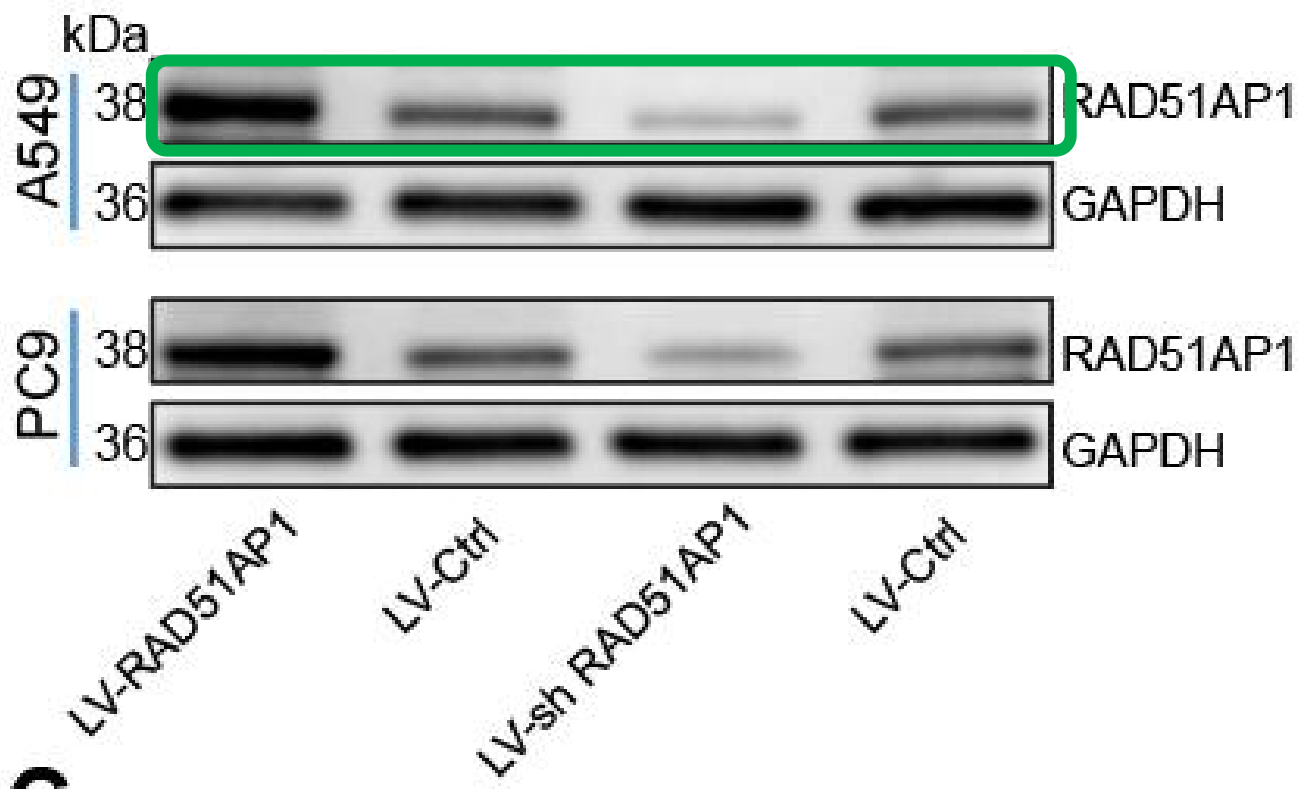

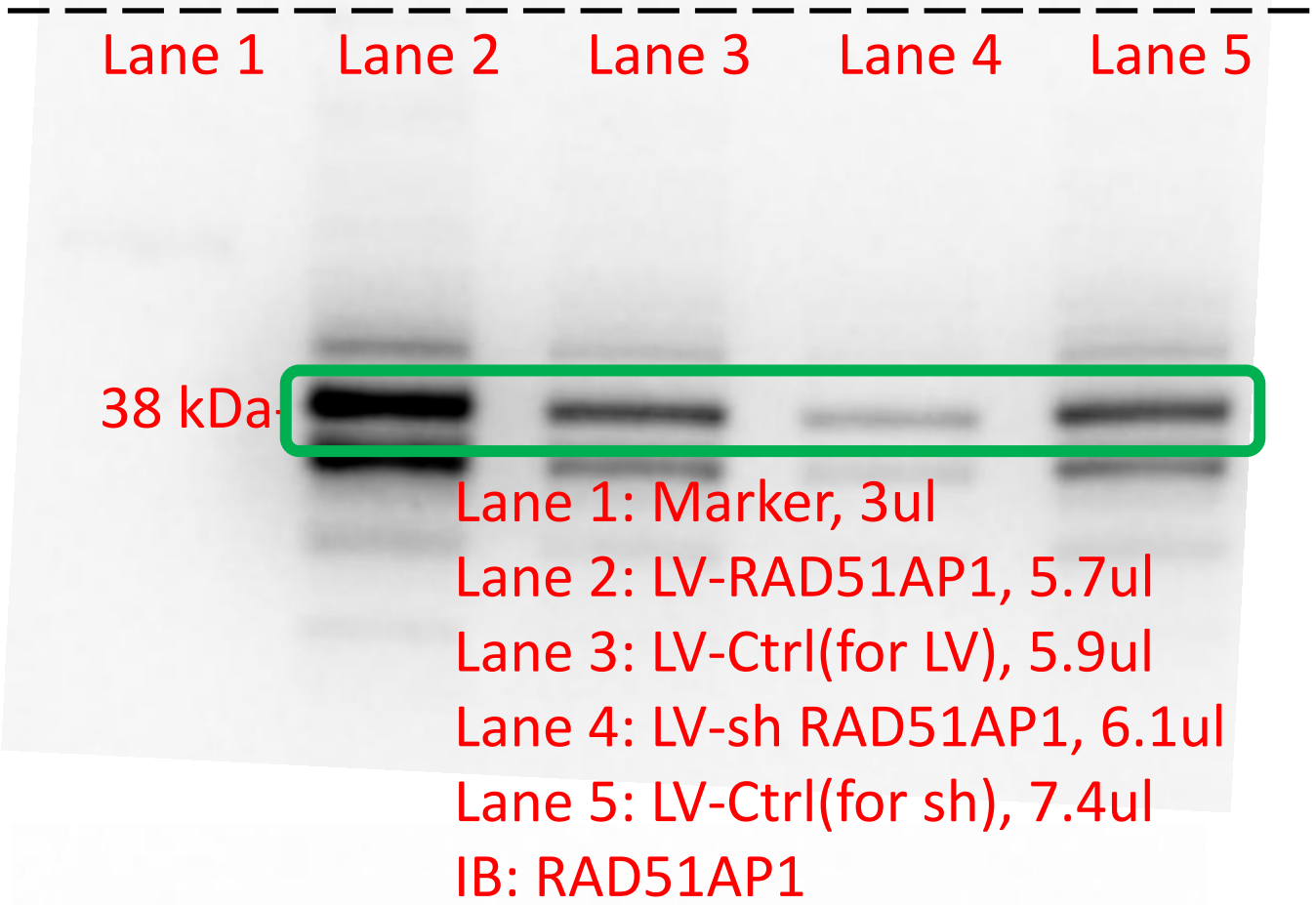

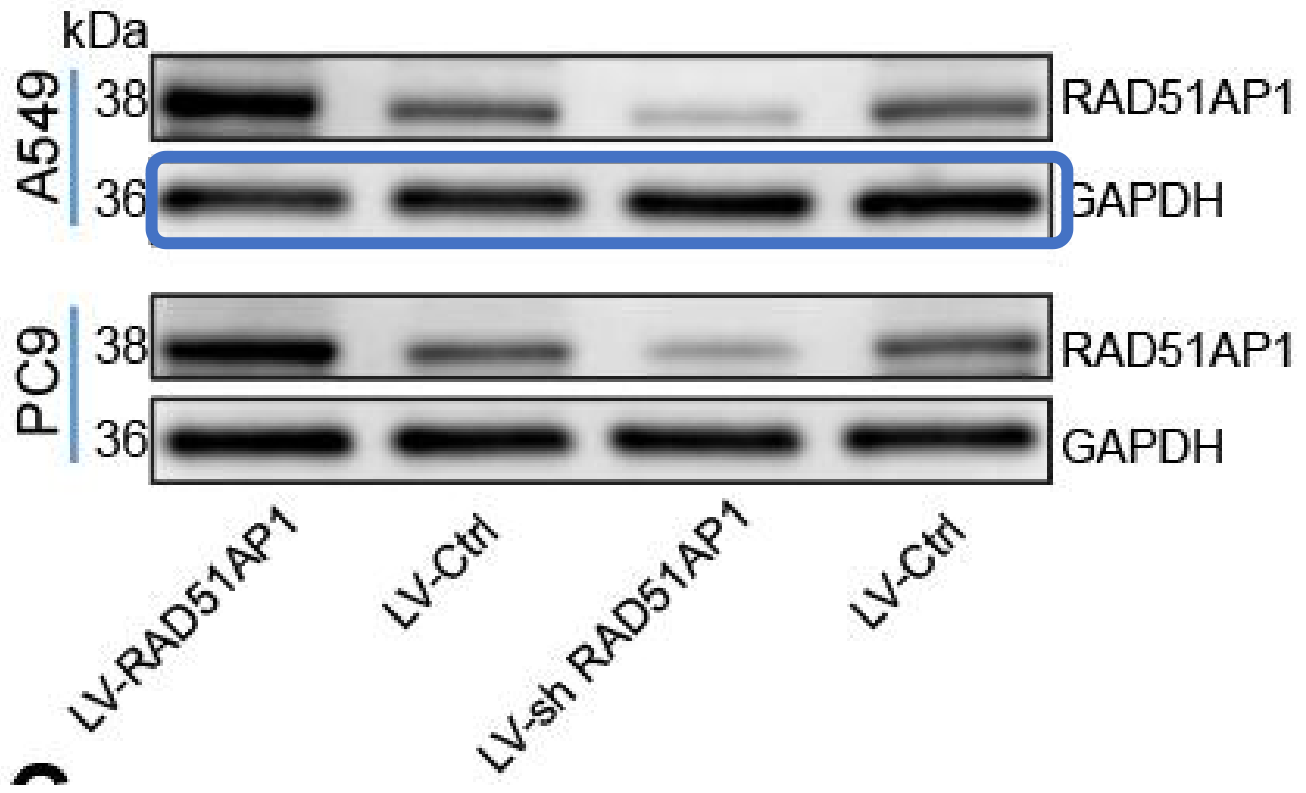

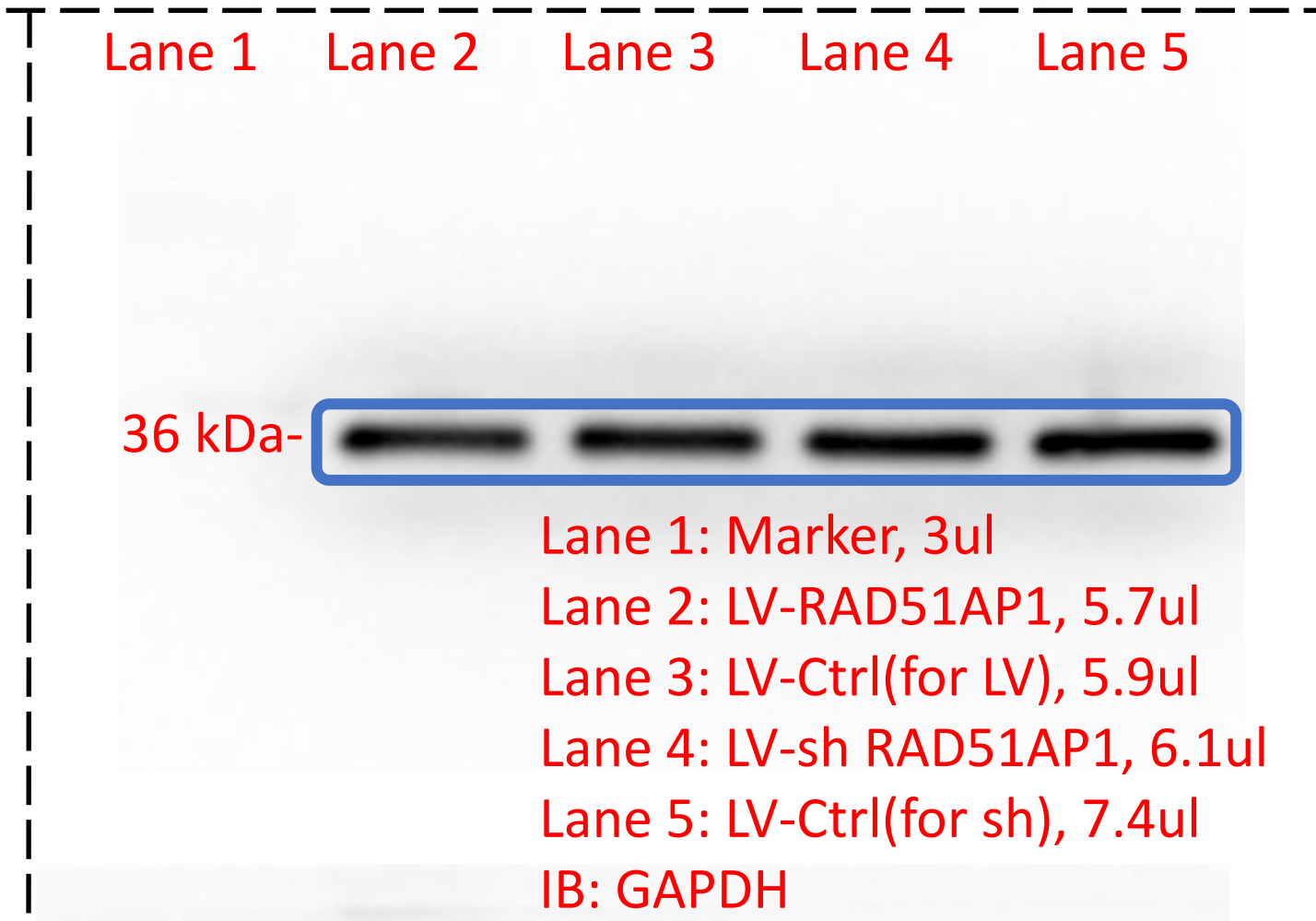

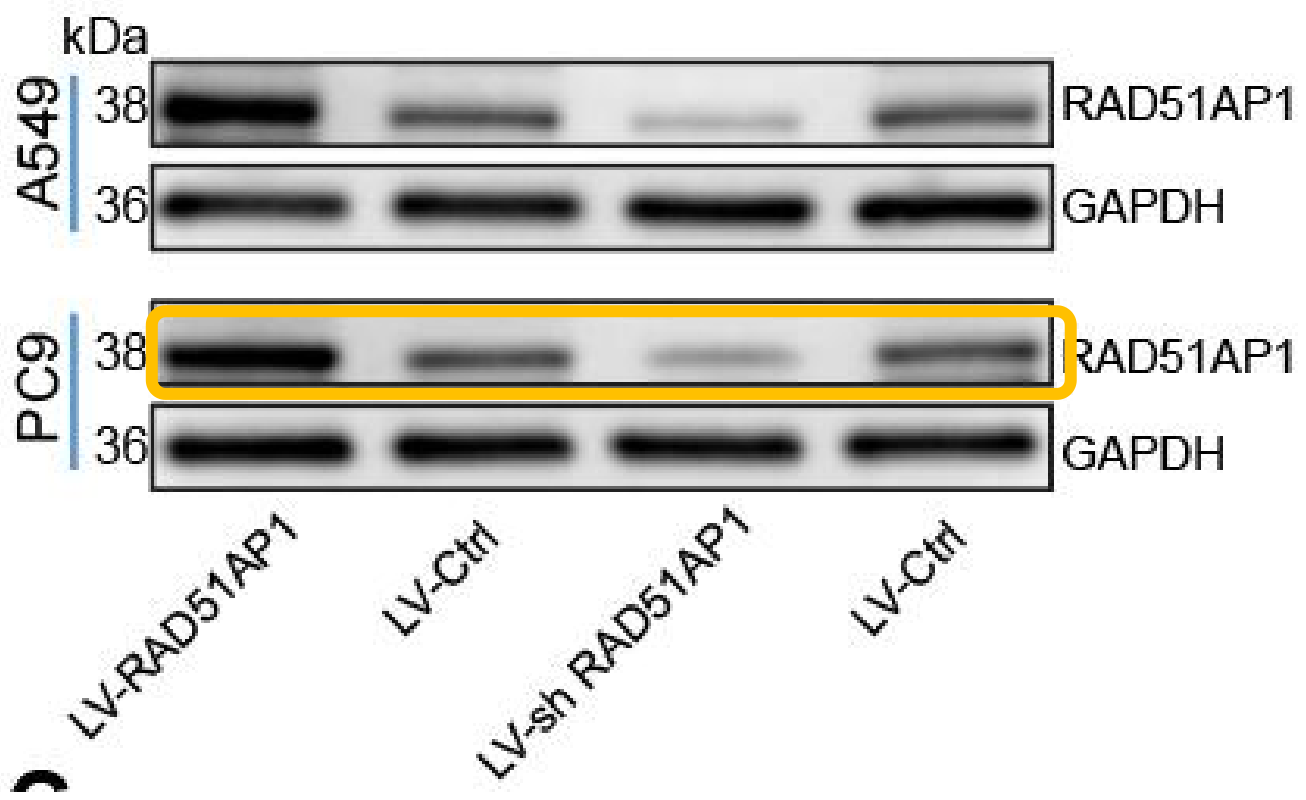

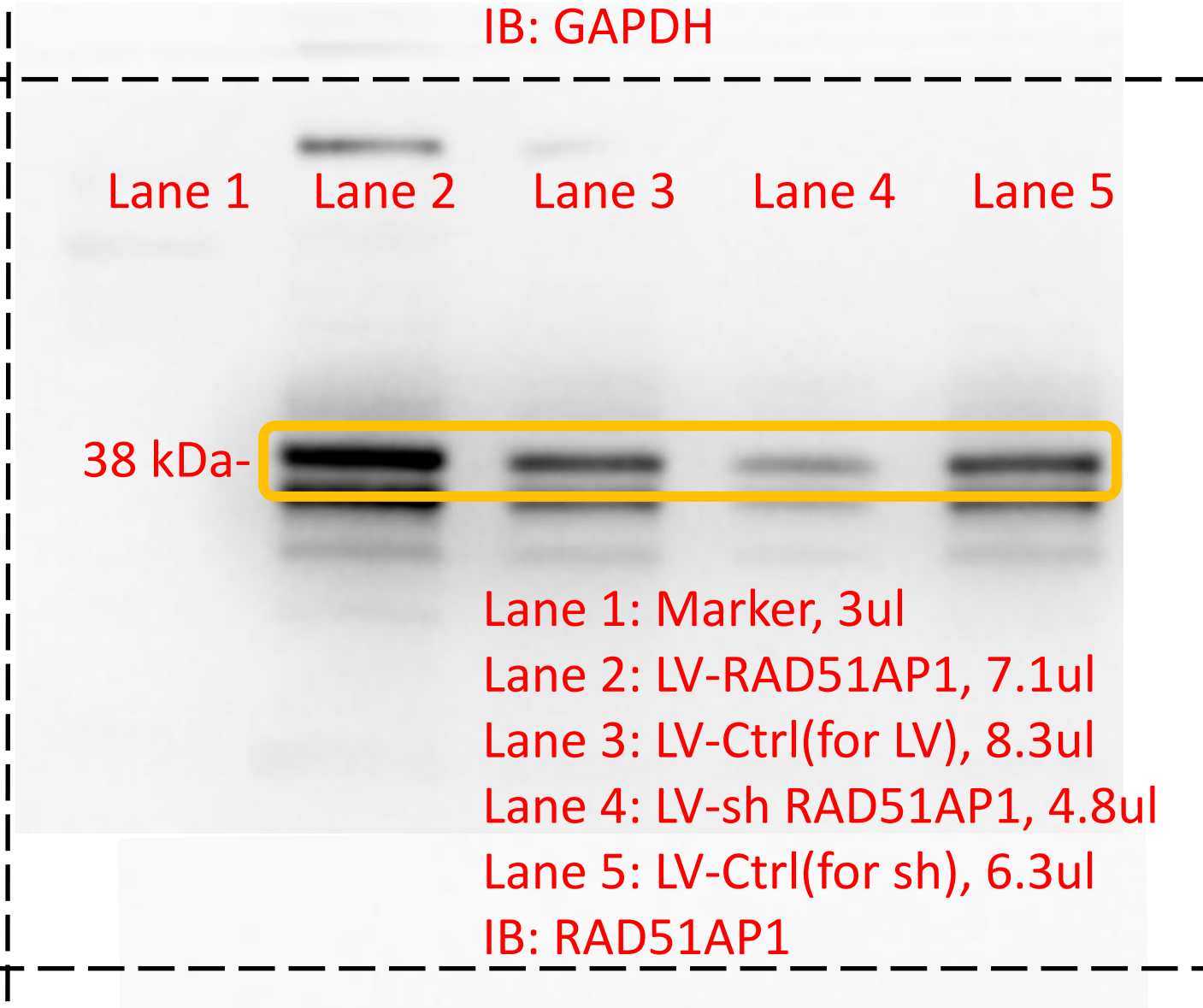

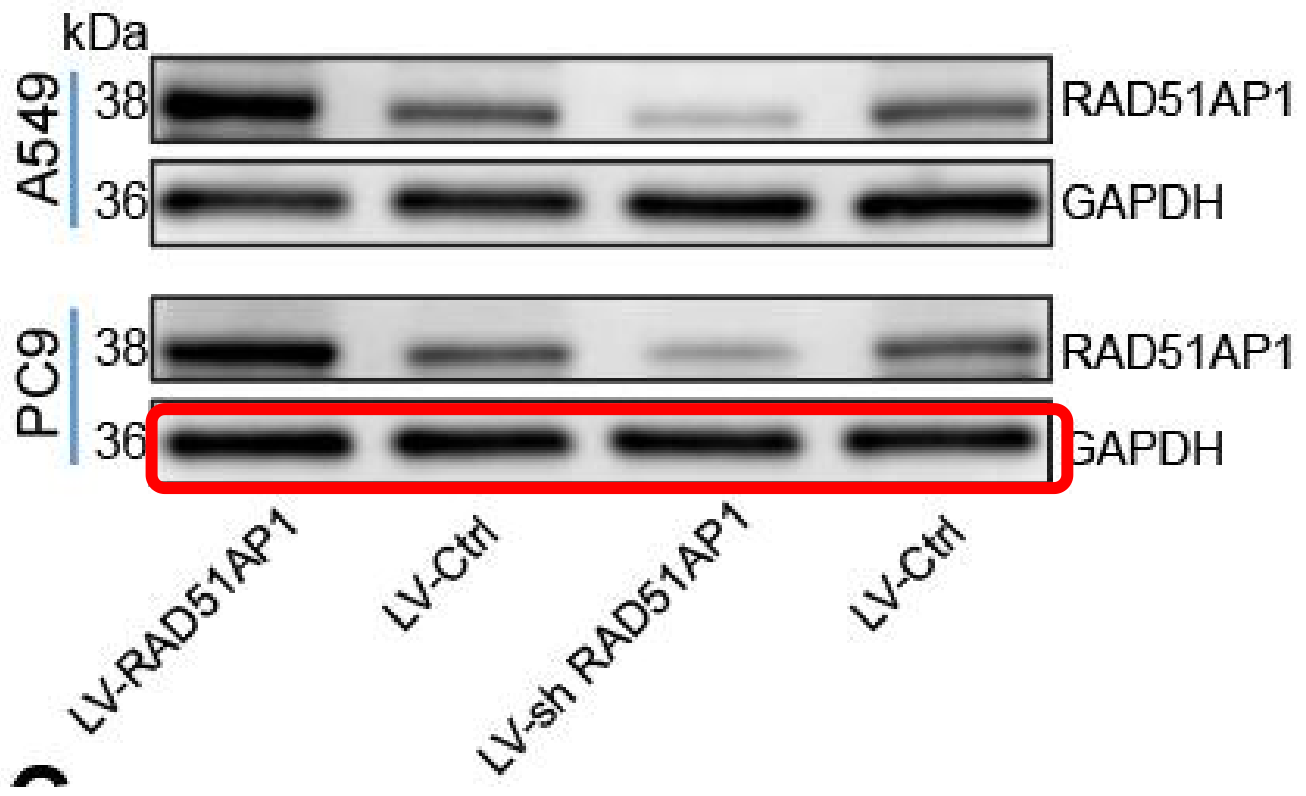

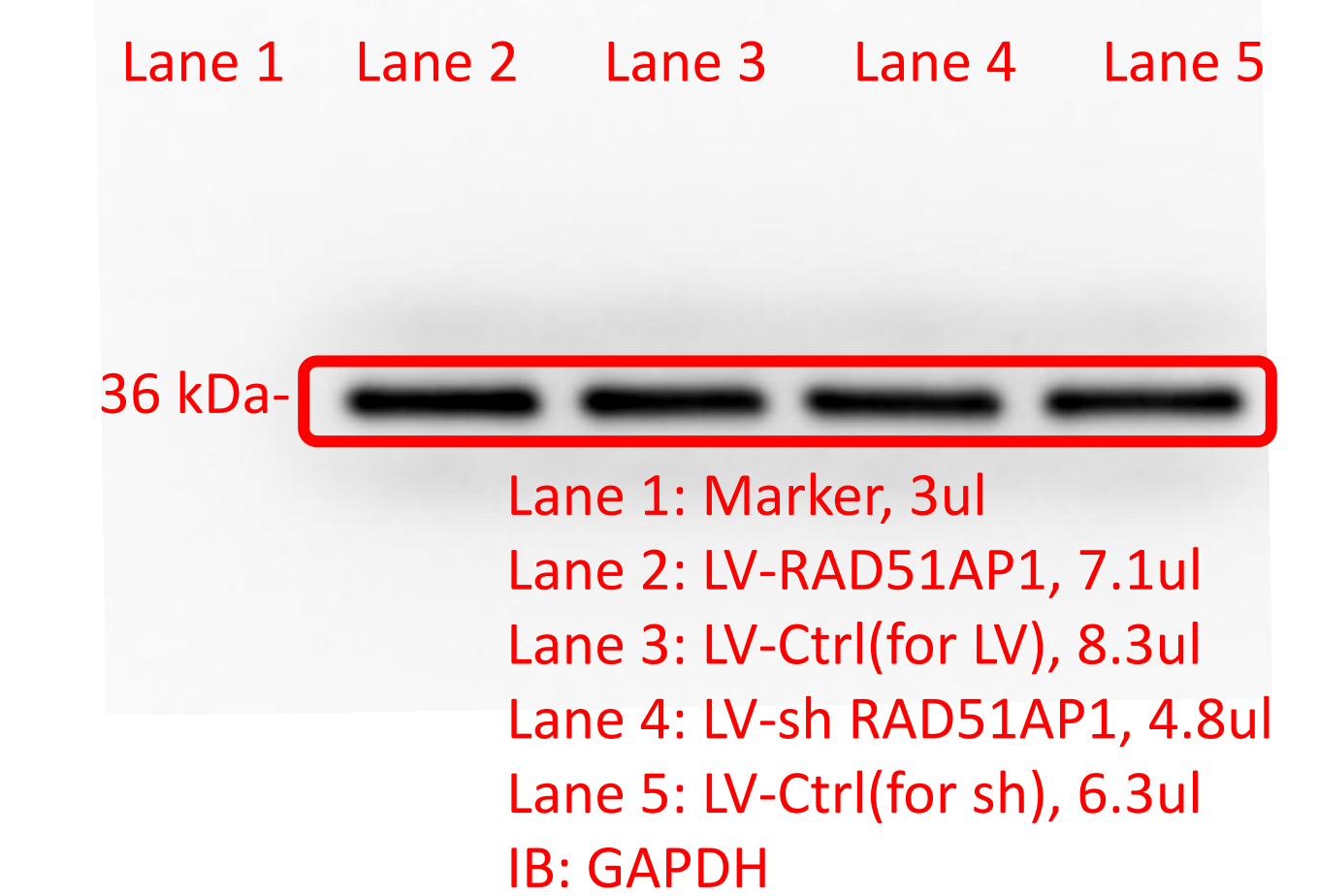

Figure 4E

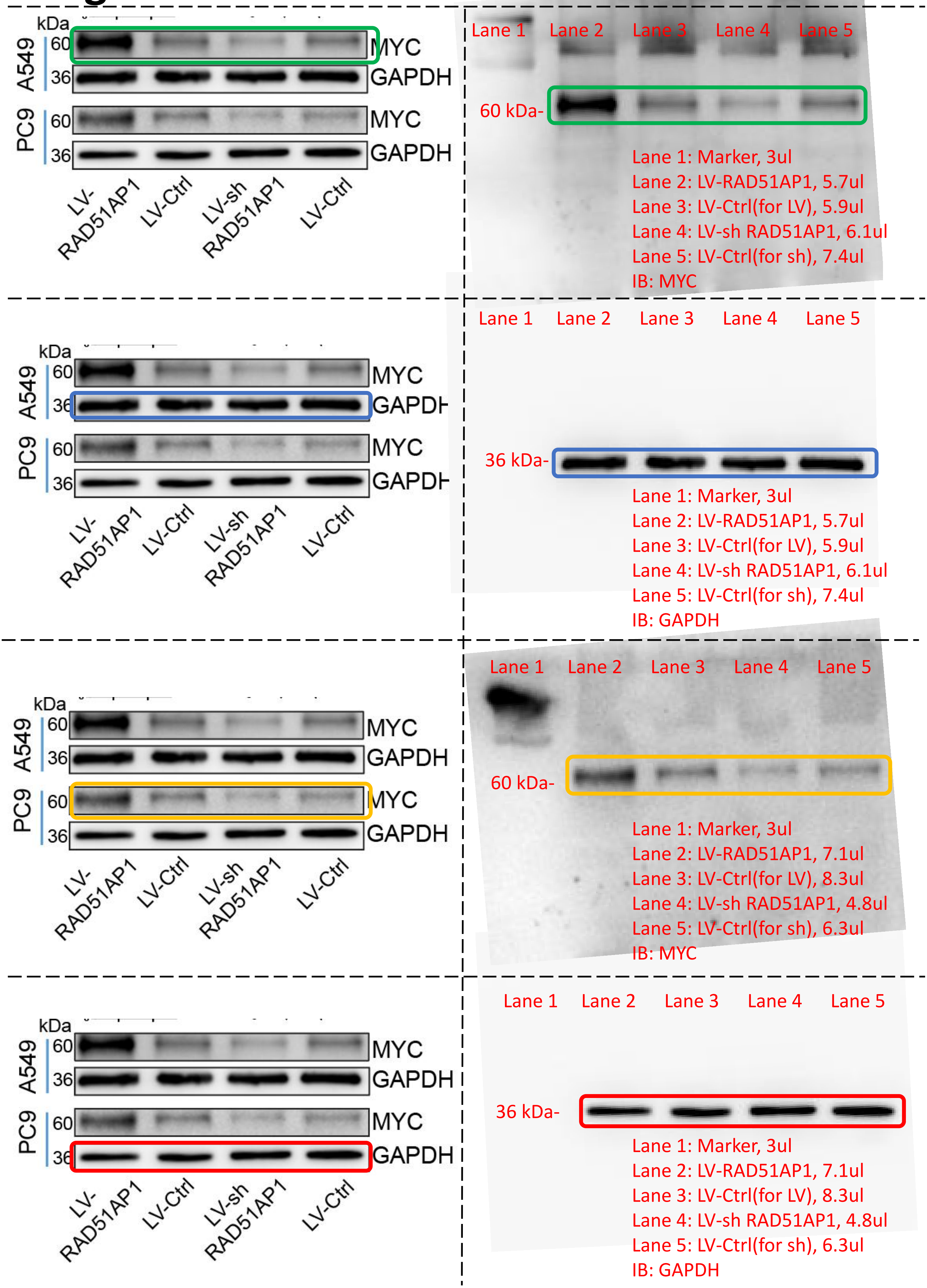
kDa
A549
60
MYC
36
GAPDH
PC9
60
MYC
36
GAPDH
LV-RAD51AP1
LV-Ctrl
LV-sh RAD51AP1
LV-Ctrl
Lane 1 Lane 2 Lane 3 Lane 4 Lane 5
60 kDa-
Lane 1: Marker, 3ul
Lane 2: LV-RAD51AP1, 5.7ul
Lane 3: LV-Ctrl(for LV), 5.9ul
Lane 4: LV-sh RAD51AP1, 6.1ul
Lane 5: LV-Ctrl(for sh), 7.4ul
IB: MYC
kDa
A549
60
MYC
36
GAPDH
PC9
60
MYC
36
GAPDH
LV-RAD51AP1
LV-Ctrl
LV-sh RAD51AP1
LV-Ctrl
Lane 1 Lane 2 Lane 3 Lane 4 Lane 5
36 kDa-
Lane 1: Marker, 3ul
Lane 2: LV-RAD51AP1, 5.7ul
Lane 3: LV-Ctrl(for LV), 5.9ul
Lane 4: LV-sh RAD51AP1, 6.1ul
Lane 5: LV-Ctrl(for sh), 7.4ul
IB: GAPDH
kDa
A549
60
MYC
36
GAPDH
PC9
60
MYC
36
GAPDH
LV-RAD51AP1
LV-Ctrl
LV-sh RAD51AP1
LV-Ctrl
Lane 1 Lane 2 Lane 3 Lane 4 Lane 5
60 kDa-
Lane 1: Marker, 3ul
Lane 2: LV-RAD51AP1, 7.1ul
Lane 3: LV-Ctrl(for LV), 8.3ul
Lane 4: LV-sh RAD51AP1, 4.8ul
Lane 5: LV-Ctrl(for sh), 6.3ul
IB: MYC
kDa
A549
60
MYC
36
GAPDH
PC9
60
MYC
36
GAPDH
LV-RAD51AP1
LV-Ctrl
LV-sh RAD51AP1
LV-Ctrl
Lane 1 Lane 2 Lane 3 Lane 4 Lane 5
36 kDa-
Lane 1: Marker, 3ul
Lane 2: LV-RAD51AP1, 7.1ul
Lane 3: LV-Ctrl(for LV), 8.3ul
Lane 4: LV-sh RAD51AP1, 4.8ul
Lane 5: LV-Ctrl(for sh), 6.3ul
IB: GAPDH

Figure 5C

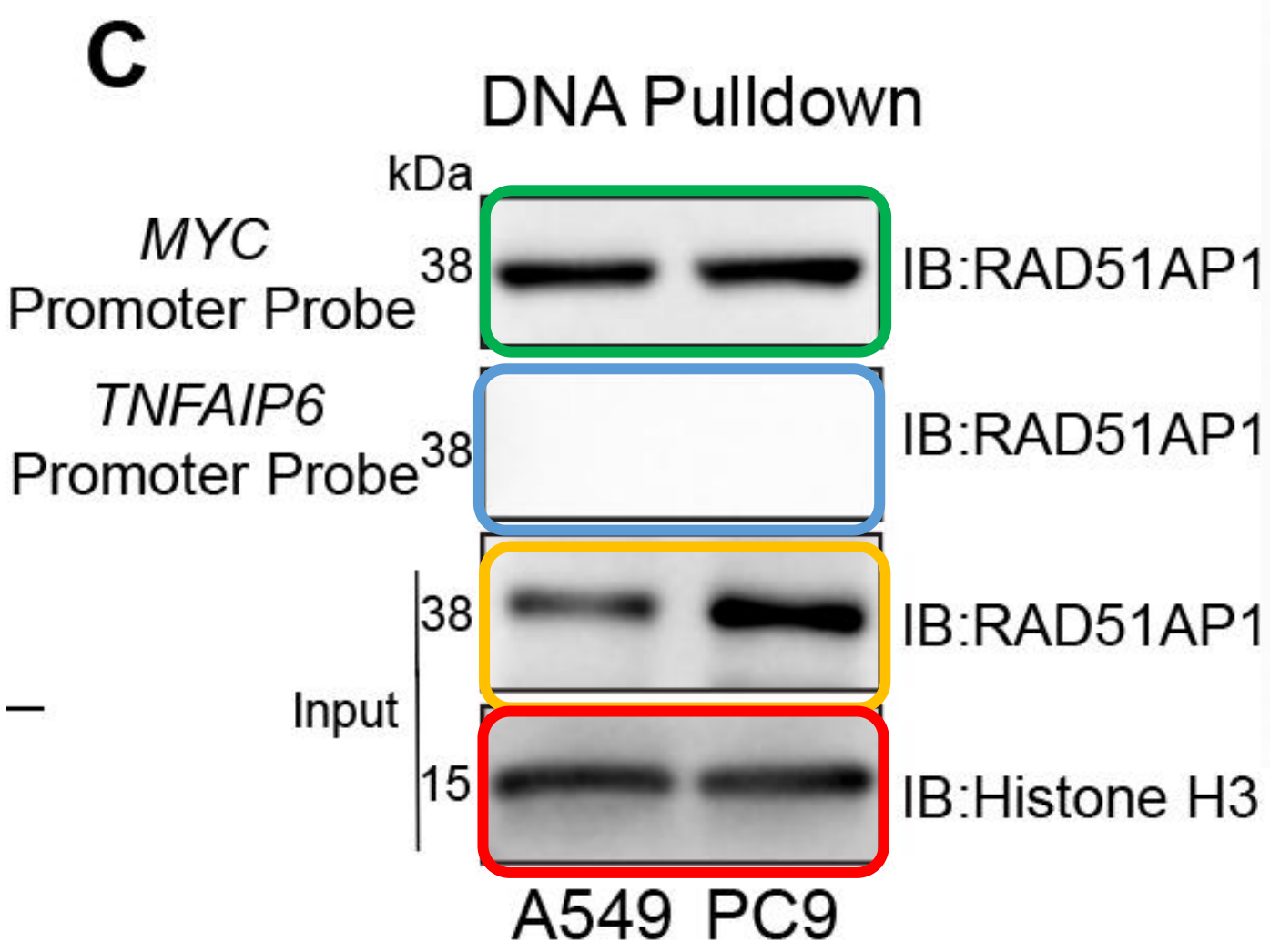

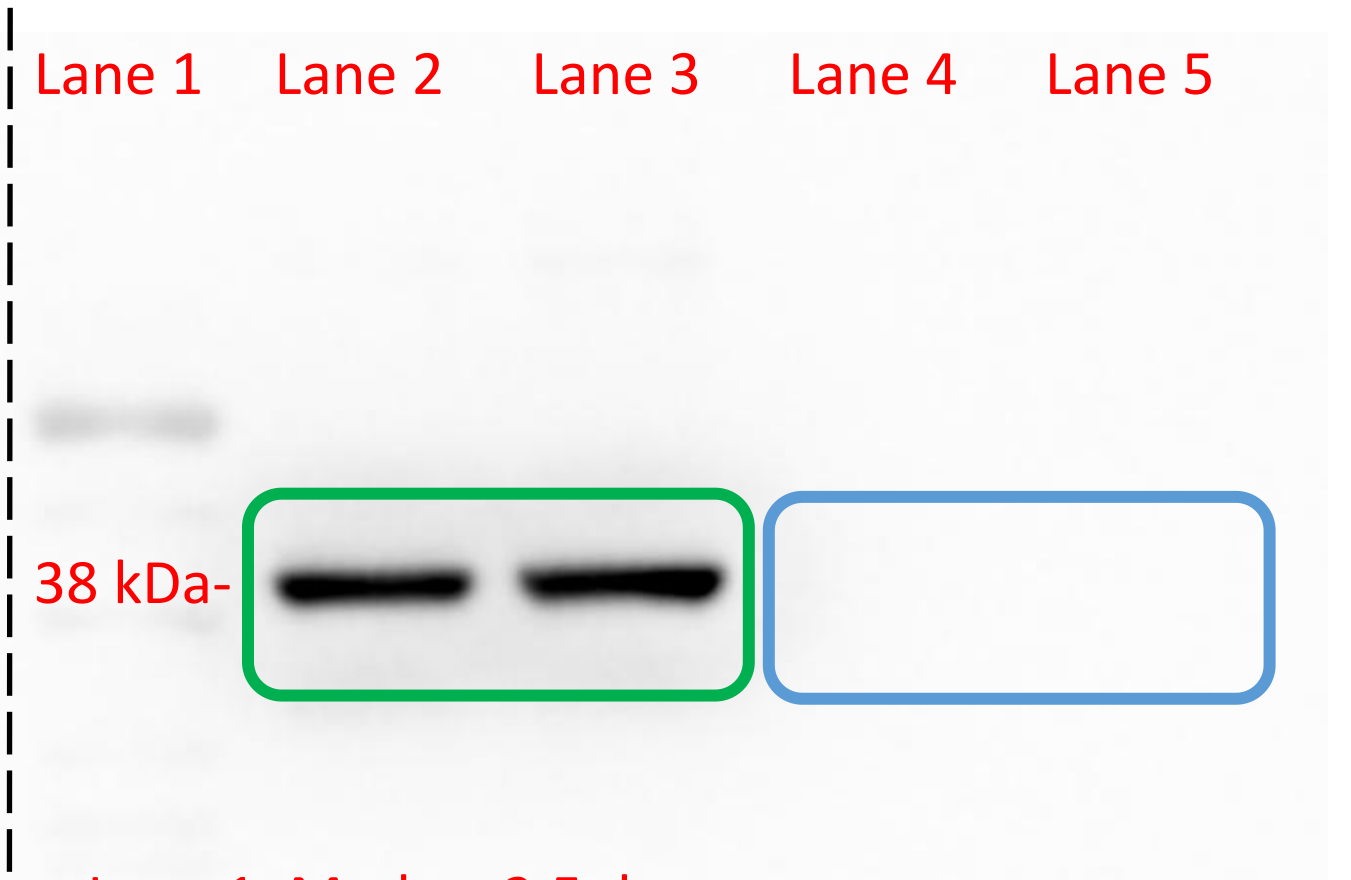

Lane 1: Marker, 2.5ul
Lane 2: *MYC* Promoter Pulldown (A549), 25ul
Lane 3: *MYC* Promoter Pulldown (PC9), 25ul
Lane 4: *TNFAIP6* Promoter Pulldown (A549), 25ul
Lane 5: *TNFAIP6* Promoter Pulldown (PC9), 25ul
IB: RAD51AP1

PS： Perform elution at a constant 37 °C for 2 h, and mix every 20 min.

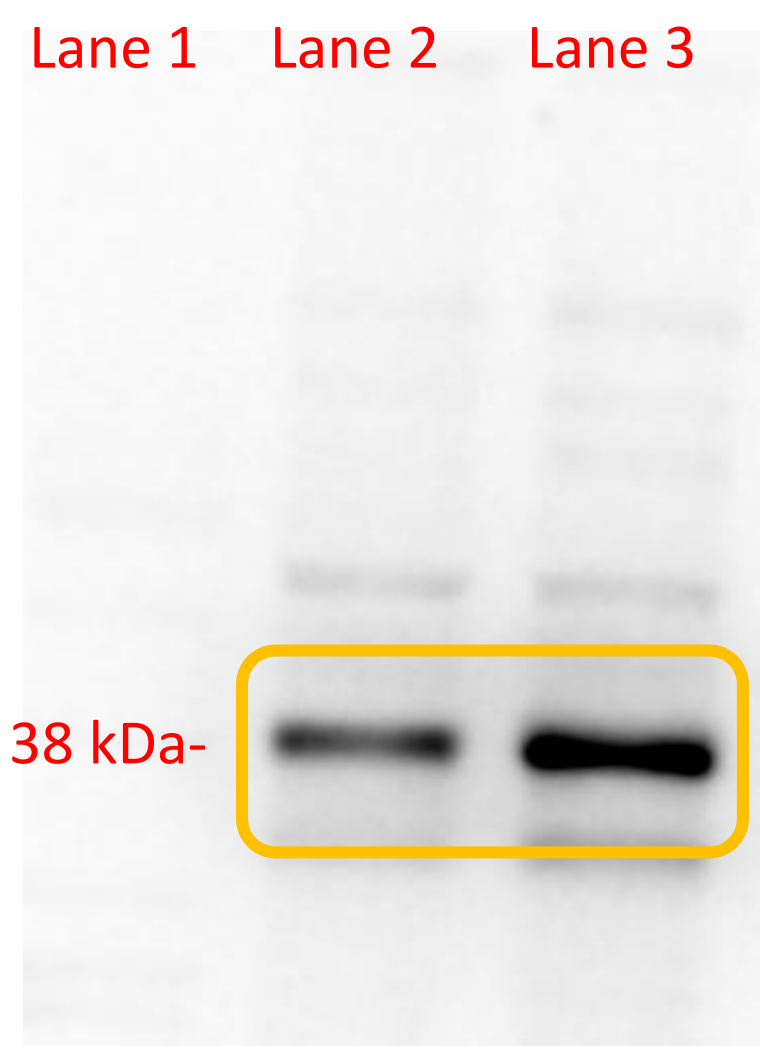

Lane 1: Marker, 2.5ul
Lane 2: Input (A549), 2.5ul
Lane 3: Input (PC9), 2.5ul
IB: RAD51AP1

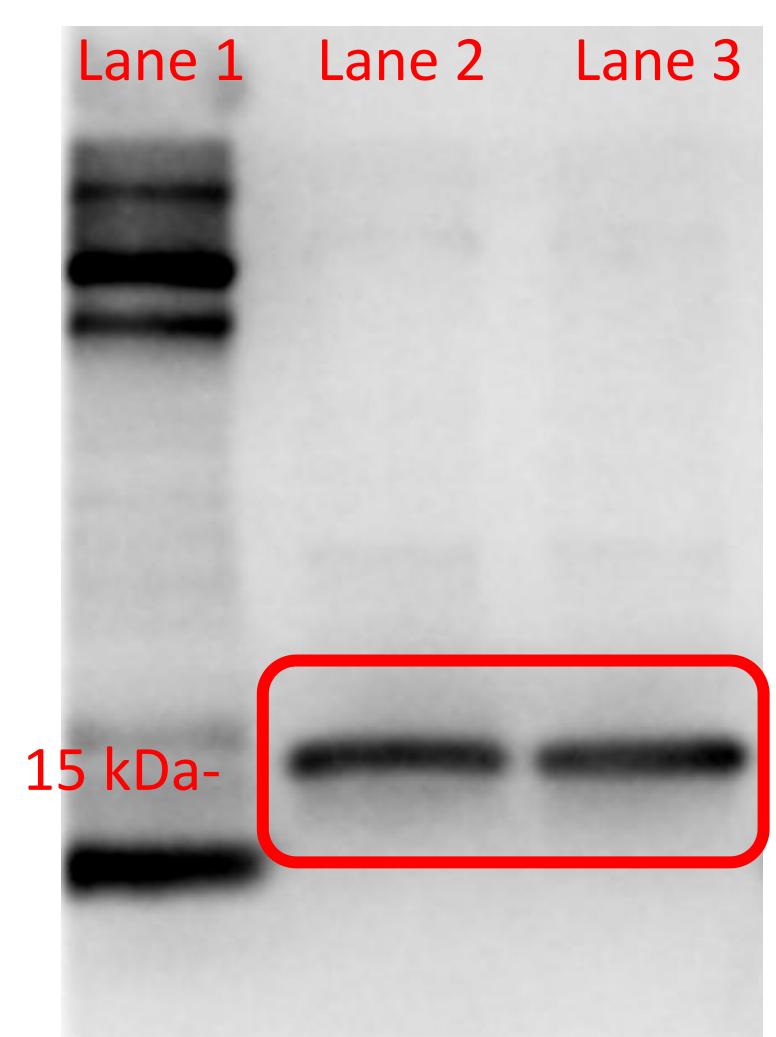

Lane 1: Marker, 2.5ul
Lane 2: Input (A549), 2.5ul
Lane 3: Input (PC9), 2.5ul
IB: Histone H3

Figure 6C

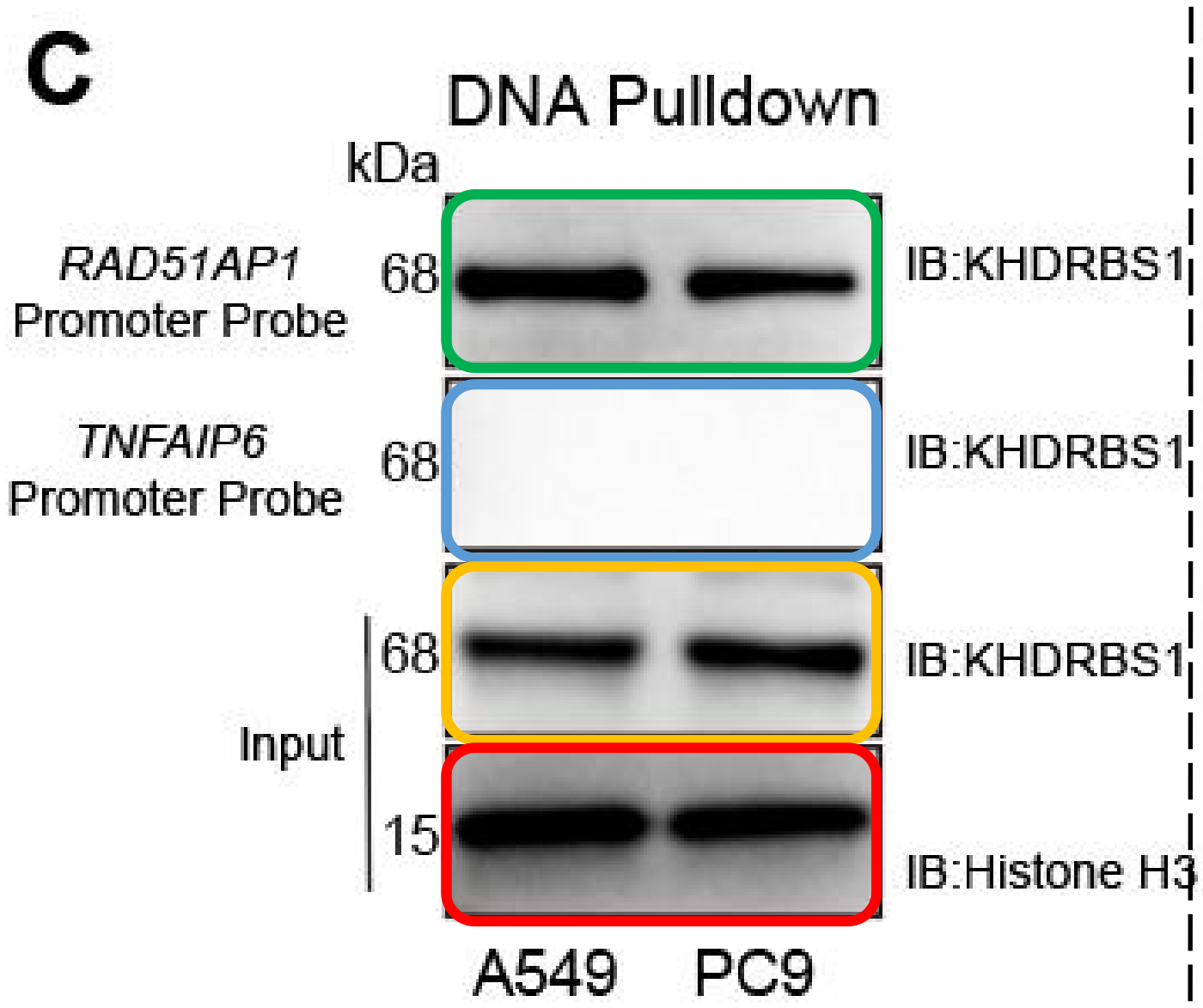

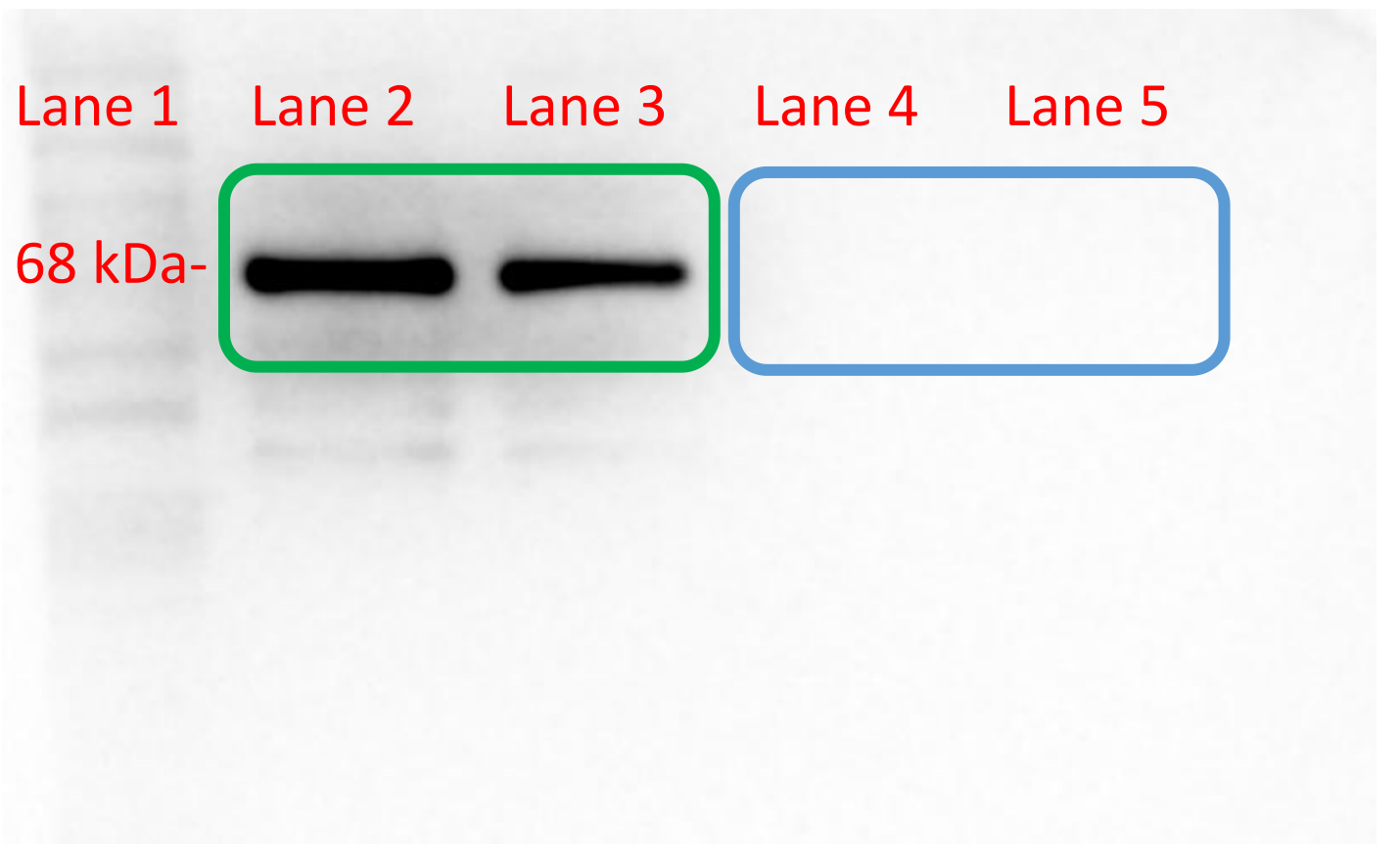

Lane 1: Marker, 2.5ul
Lane 2: *RAD51AP1* Promoter Pulldown (A549), 25ul
Lane 3: *RAD51AP1* Promoter Pulldown (PC9), 25ul
Lane 4: *TNFAIP6* Promoter Pulldown (A549), 25ul
Lane 5: *TNFAIP6* Promoter Pulldown (PC9), 25ul
IB: KHDRBS1

PS： Perform elution at a constant 37 °C for 2 h, and mix every 20 min.

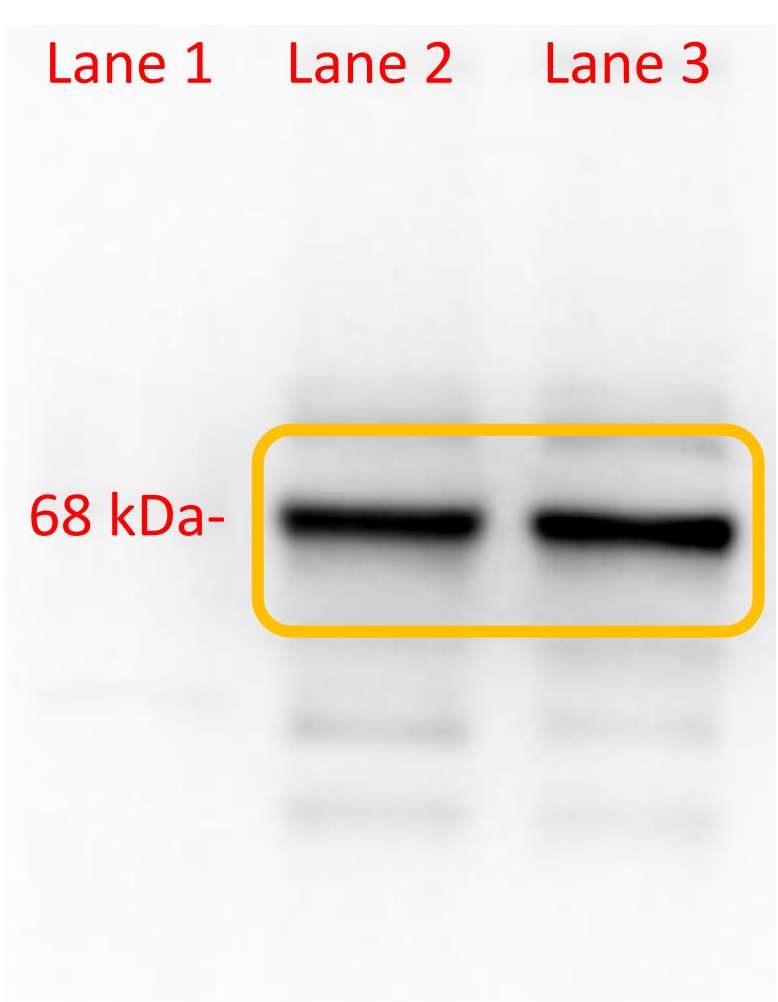

Lane 1: Marker, 2.5ul
Lane 2: Input (A549), 2.5ul
Lane 3: Input (PC9), 2.5ul
IB: KHDRBS1

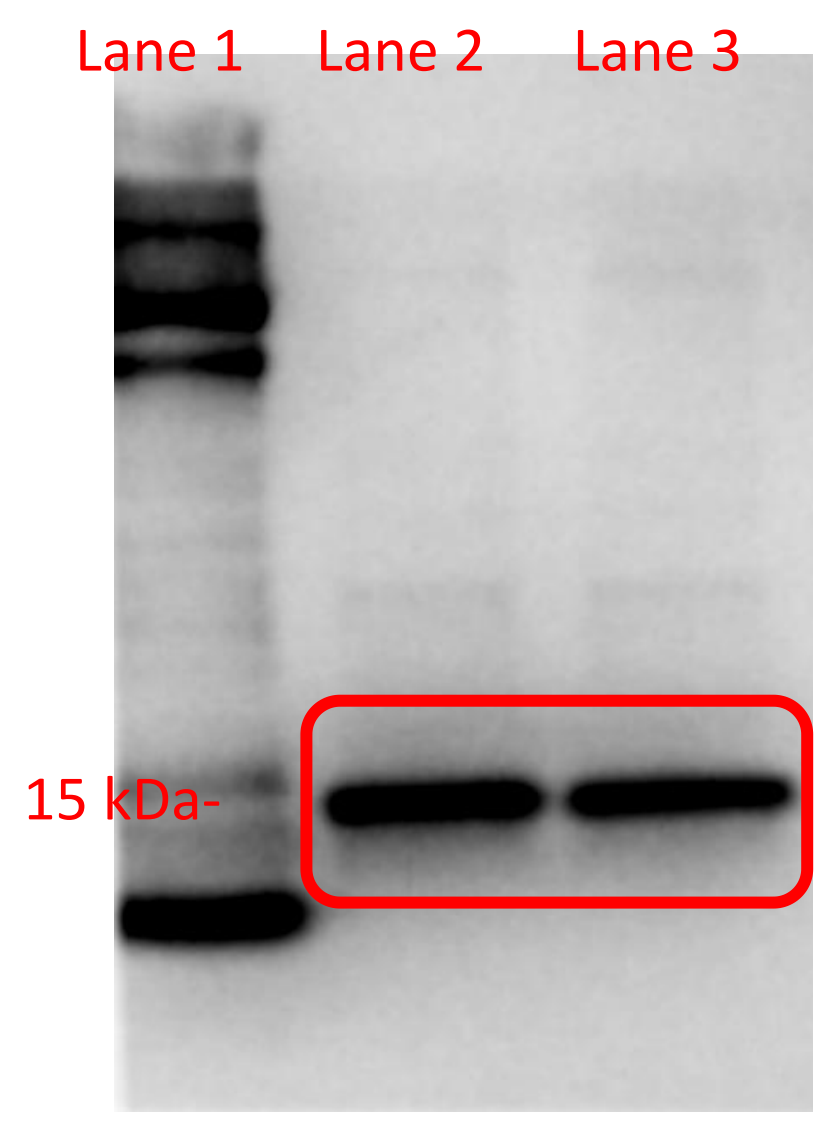

Lane 1: Marker, 2.5ul
Lane 2: Input (A549), 2.5ul
Lane 3: Input (PC9), 2.5ul
IB: Histone H3

Figure 6E

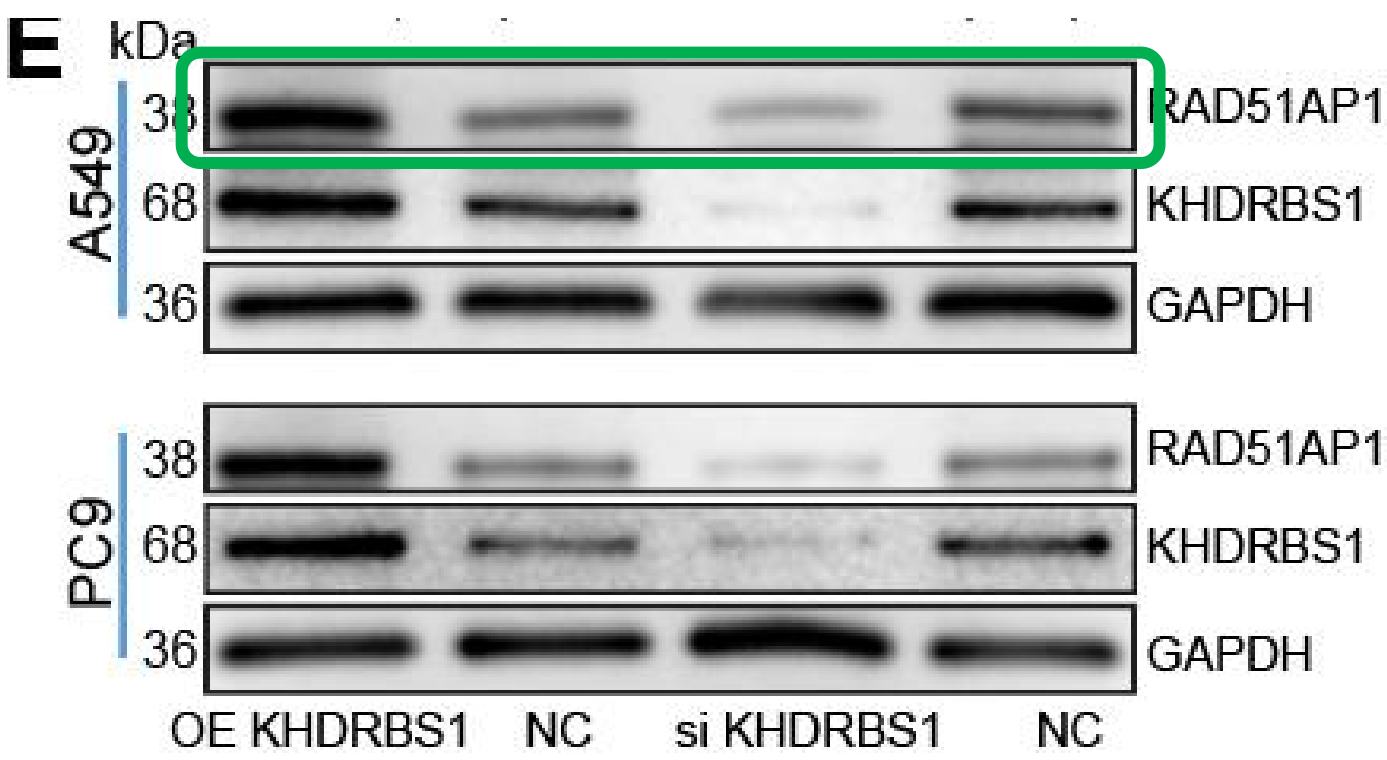
E
kDa
A549
PC9
38
68
36
RAD51AP1
KHDRBS1
GAPDH
OE KHDRBS1
NC
si KHDRBS1
NC

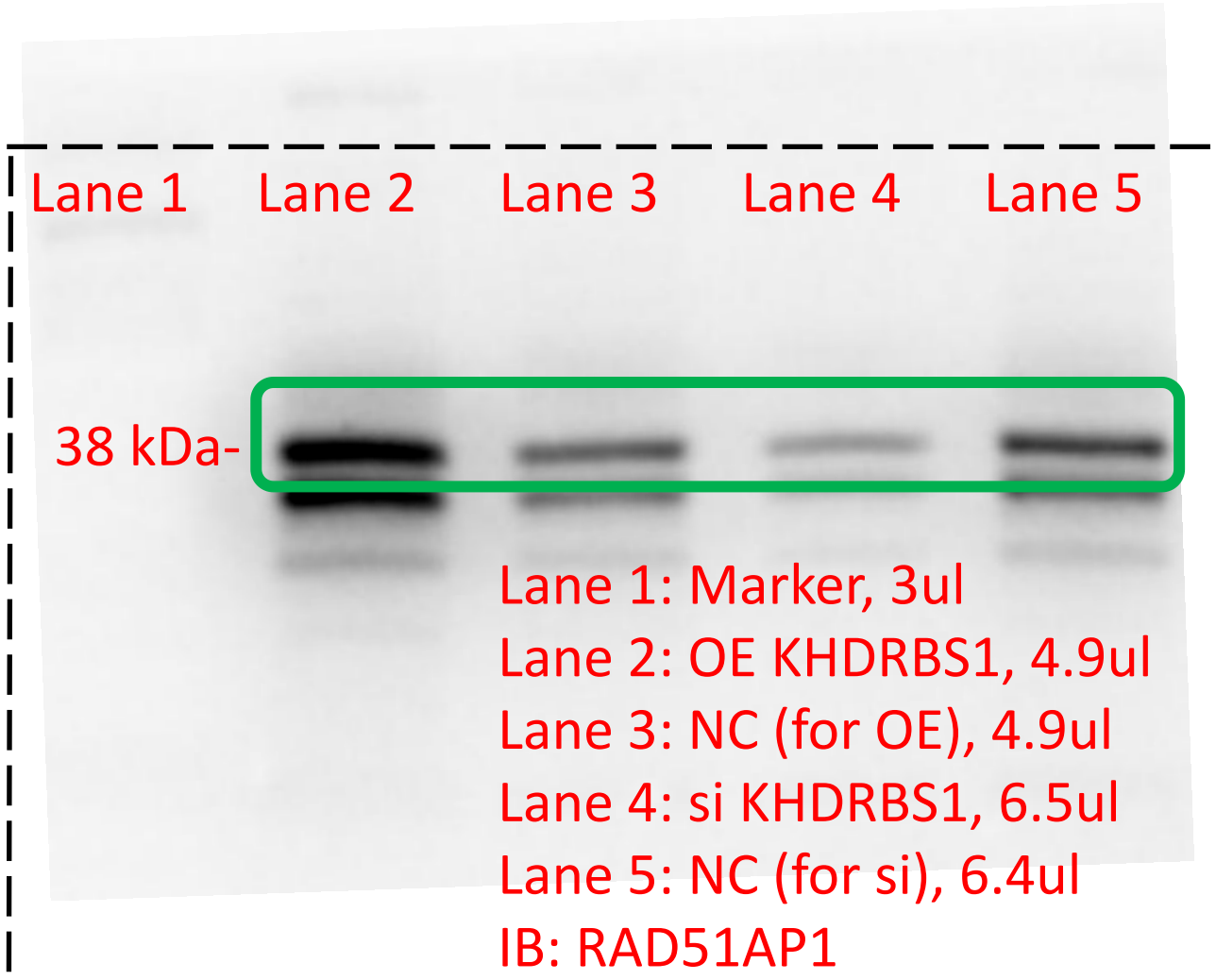
Lane 1
Lane 2
Lane 3
Lane 4
Lane 5
38 kDa-
Lane 1: Marker, 3ul
Lane 2: OE KHDRBS1, 4.9ul
Lane 3: NC (for OE), 4.9ul
Lane 4: si KHDRBS1, 6.5ul
Lane 5: NC (for si), 6.4ul
IB: RAD51AP1

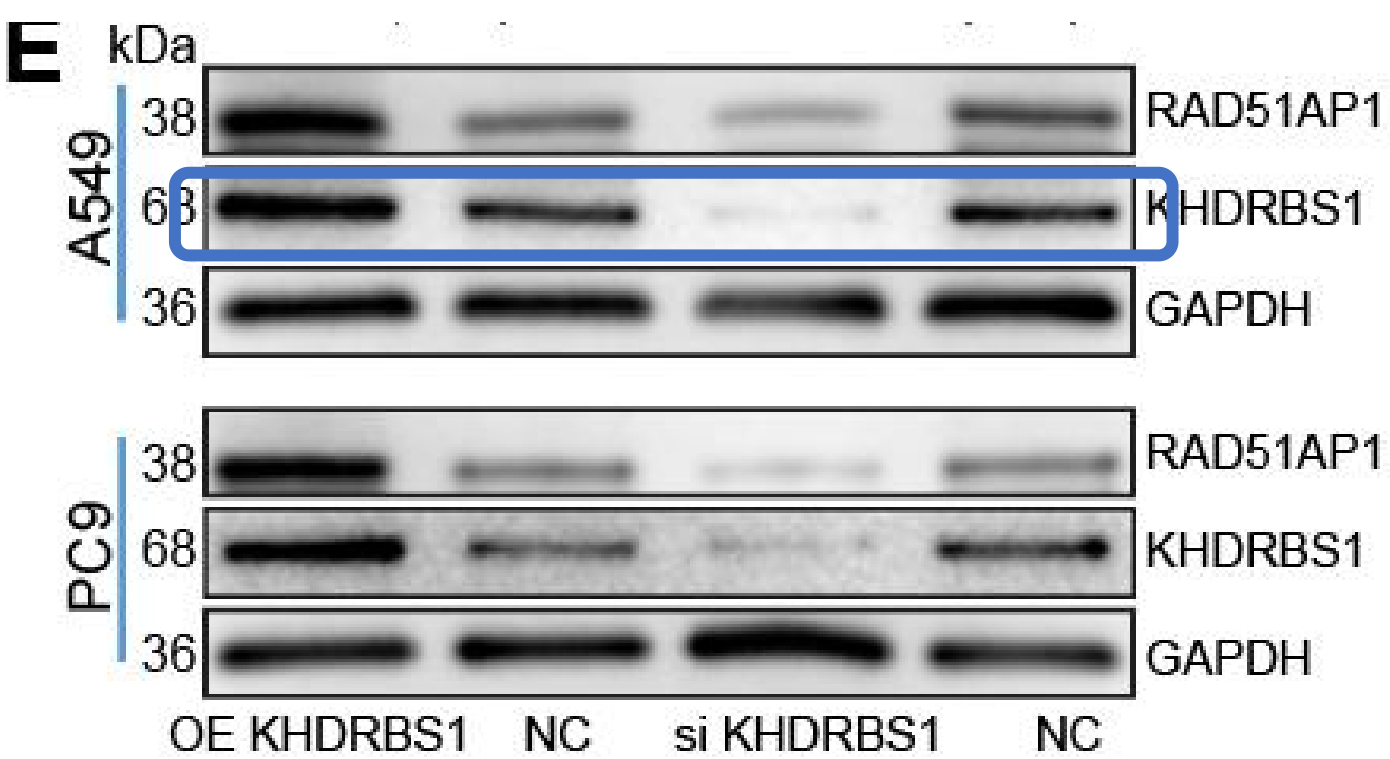
E
kDa
A549
PC9
38
68
36
RAD51AP1
KHDRBS1
GAPDH
OE KHDRBS1
NC
si KHDRBS1
NC

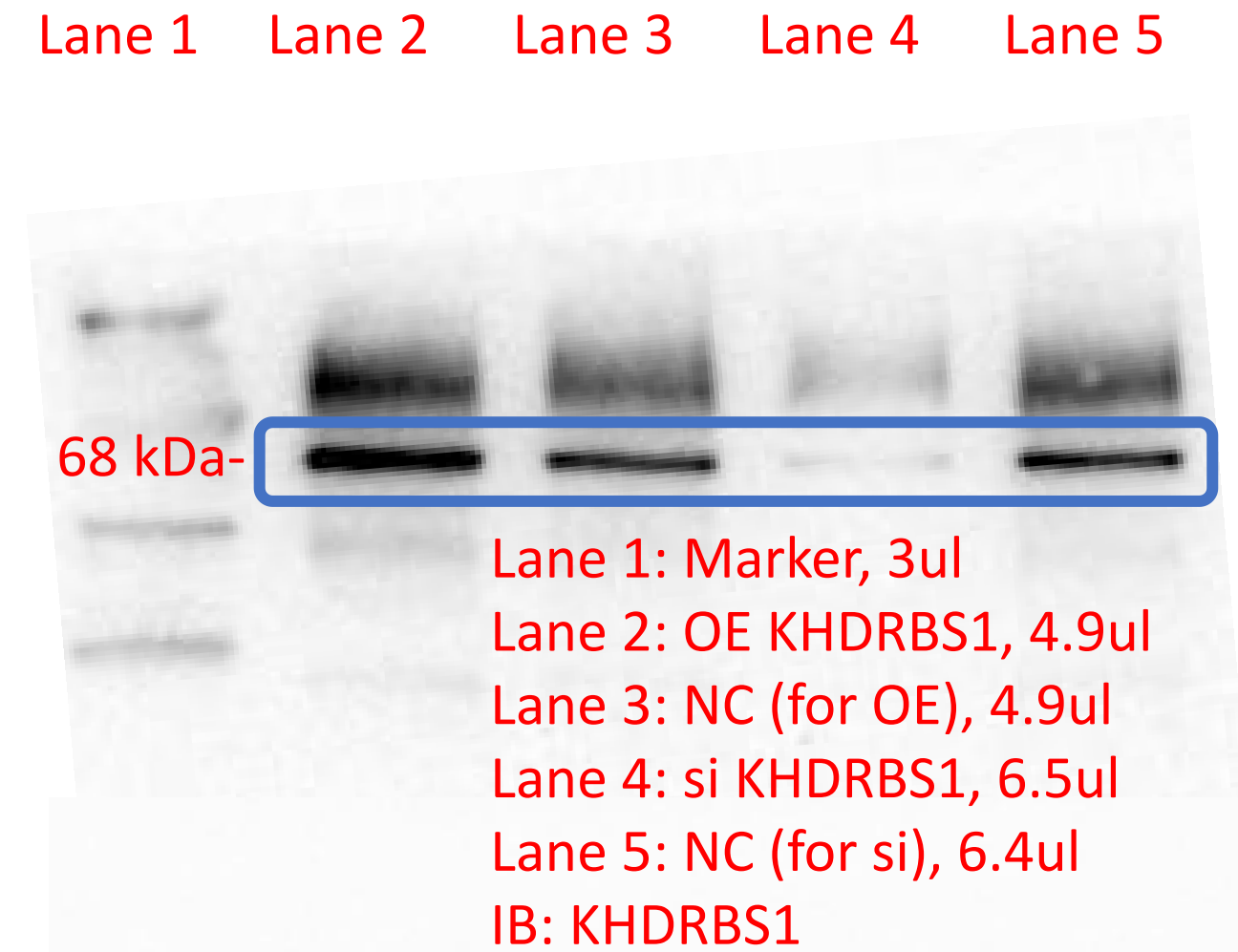
Lane 1
Lane 2
Lane 3
Lane 4
Lane 5
68 kDa-
Lane 1: Marker, 3ul
Lane 2: OE KHDRBS1, 4.9ul
Lane 3: NC (for OE), 4.9ul
Lane 4: si KHDRBS1, 6.5ul
Lane 5: NC (for si), 6.4ul
IB: KHDRBS1

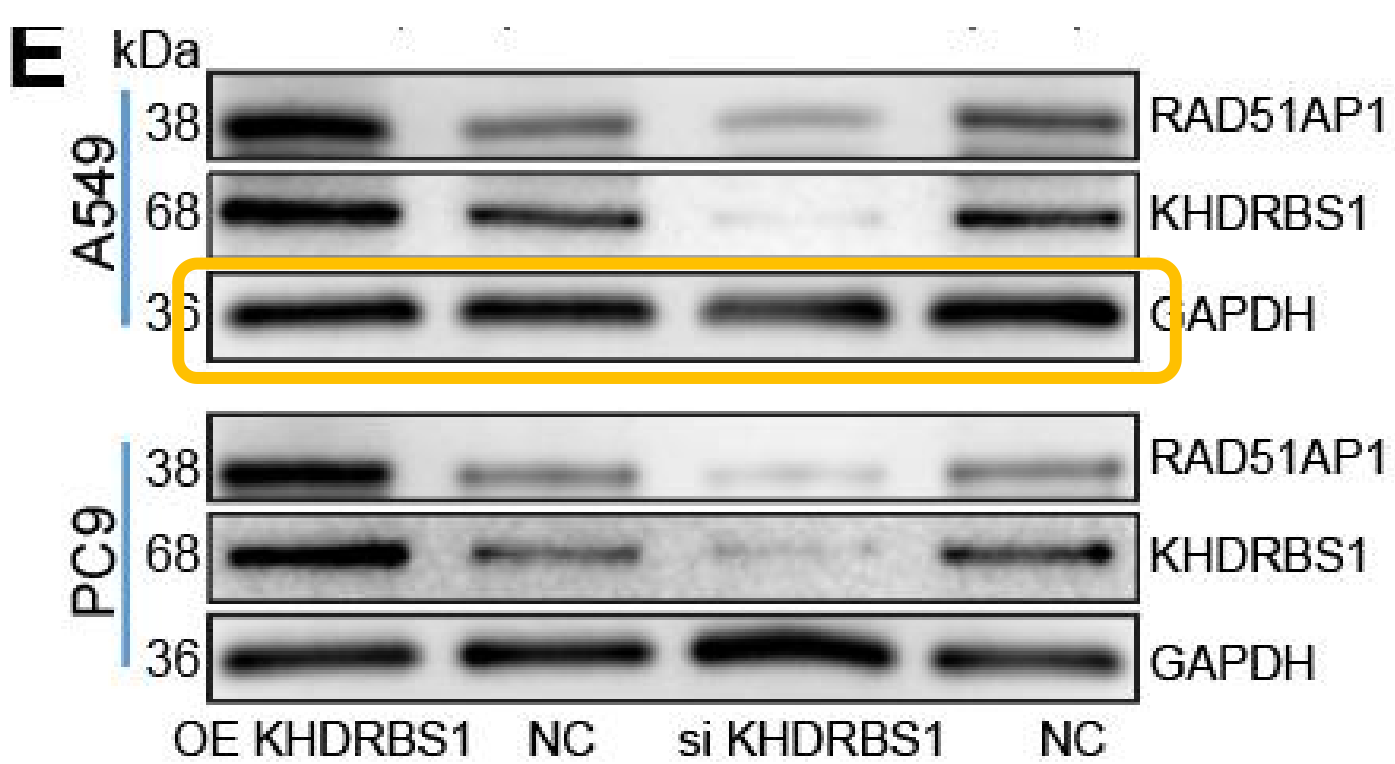
E
kDa
A549
PC9
38
68
36
RAD51AP1
KHDRBS1
GAPDH
OE KHDRBS1
NC
si KHDRBS1
NC

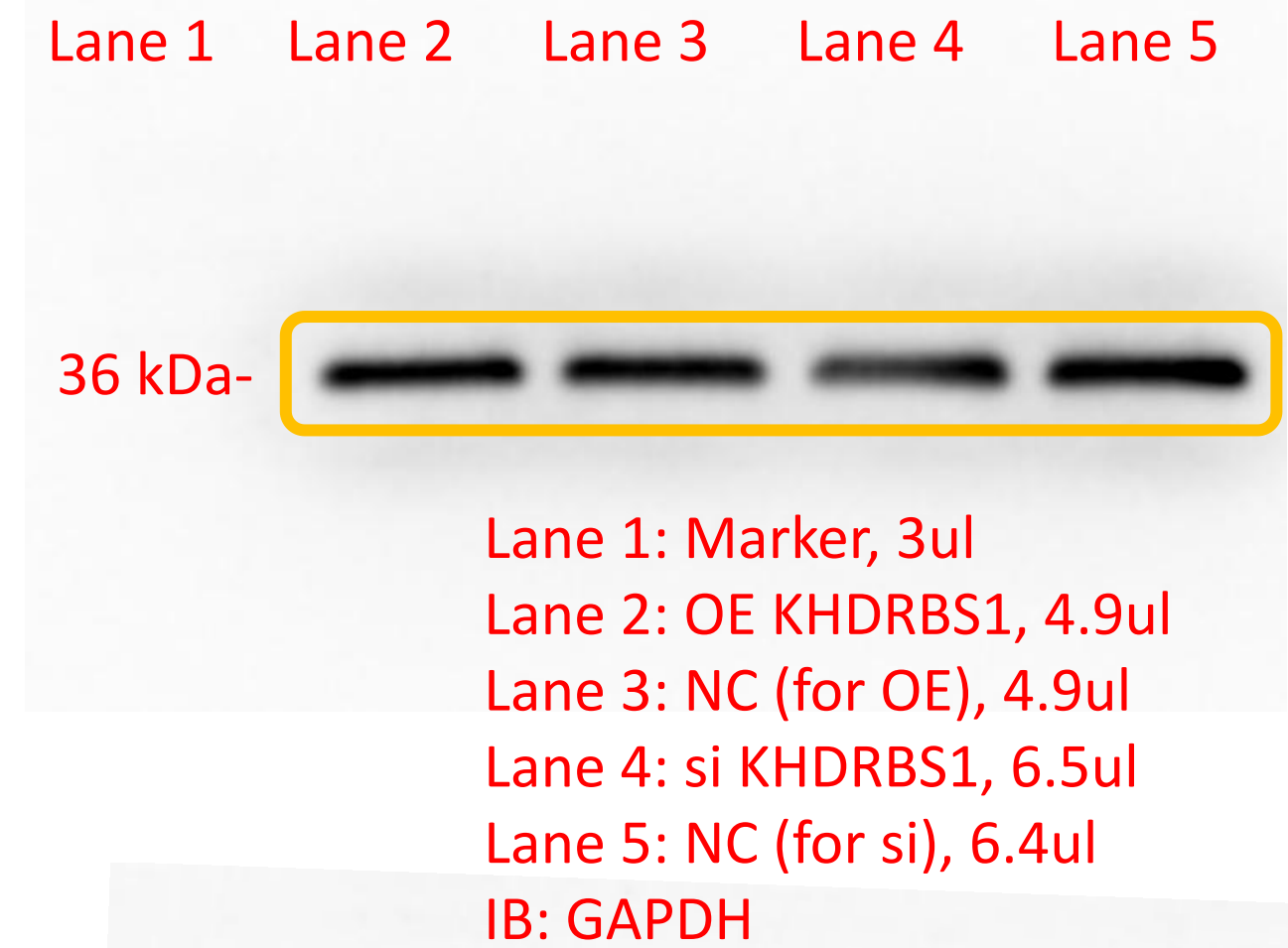
Lane 1
Lane 2
Lane 3
Lane 4
Lane 5
36 kDa-
Lane 1: Marker, 3ul
Lane 2: OE KHDRBS1, 4.9ul
Lane 3: NC (for OE), 4.9ul
Lane 4: si KHDRBS1, 6.5ul
Lane 5: NC (for si), 6.4ul
IB: GAPDH

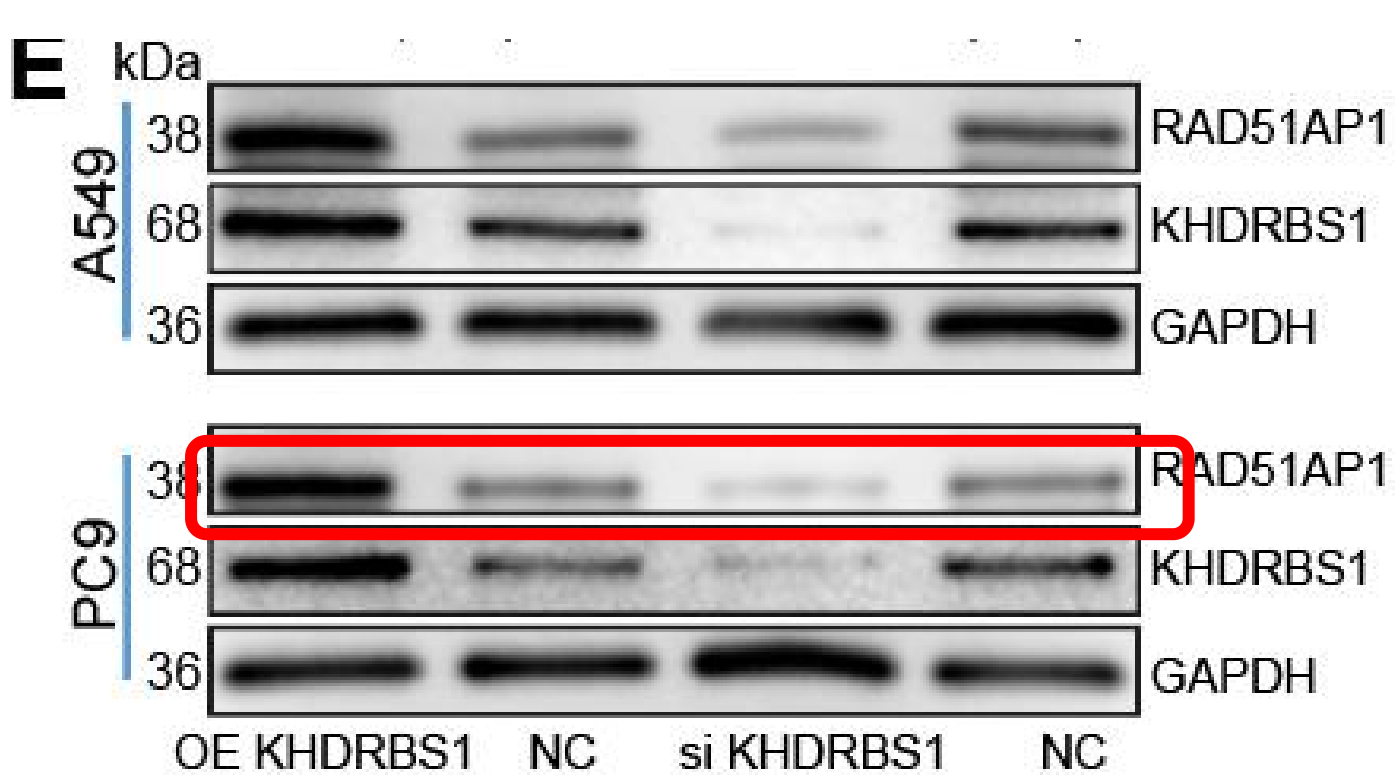
E
kDa
A549
PC9
38
68
36
RAD51AP1
KHDRBS1
GAPDH
OE KHDRBS1
NC
si KHDRBS1
NC

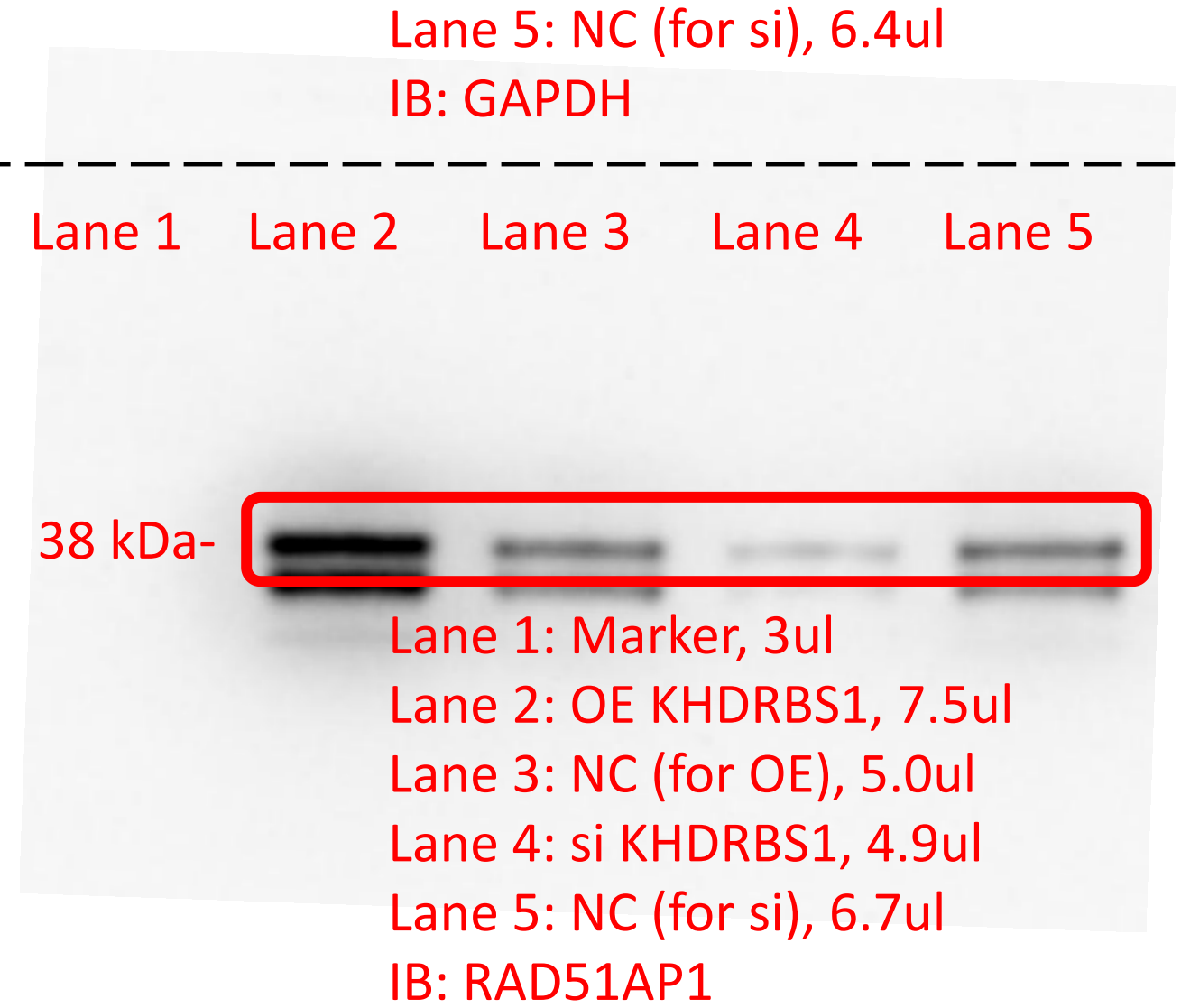
Lane 1
Lane 2
Lane 3
Lane 4
Lane 5
38 kDa-
Lane 1: Marker, 3ul
Lane 2: OE KHDRBS1, 7.5ul
Lane 3: NC (for OE), 5.0ul
Lane 4: si KHDRBS1, 4.9ul
Lane 5: NC (for si), 6.7ul
IB: RAD51AP1

Figure 6E

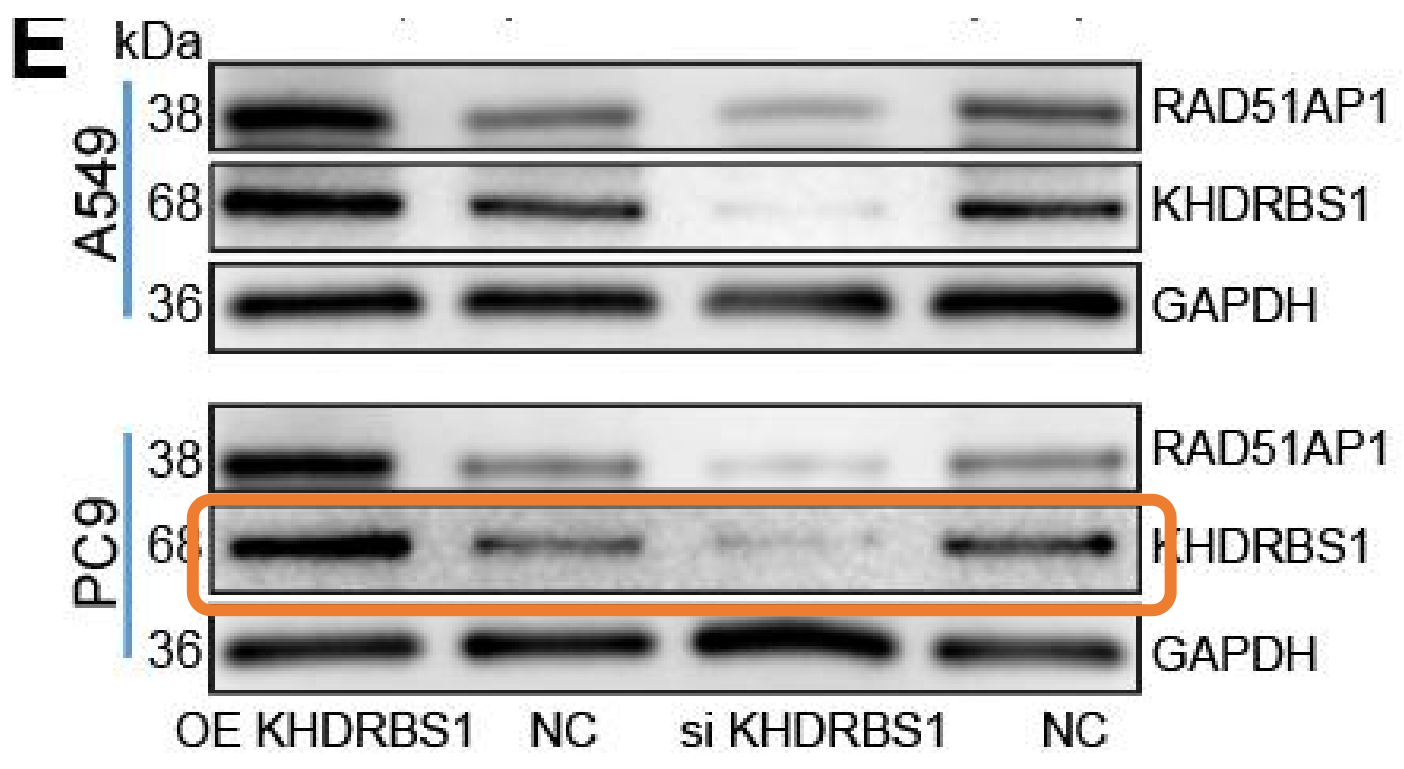
E
kDa
A549
38
68
36
RAD51AP1
KHDRBS1
GAPDH
PC9
38
68
36
RAD51AP1
KHDRBS1
GAPDH
OE KHDRBS1
NC
si KHDRBS1
NC

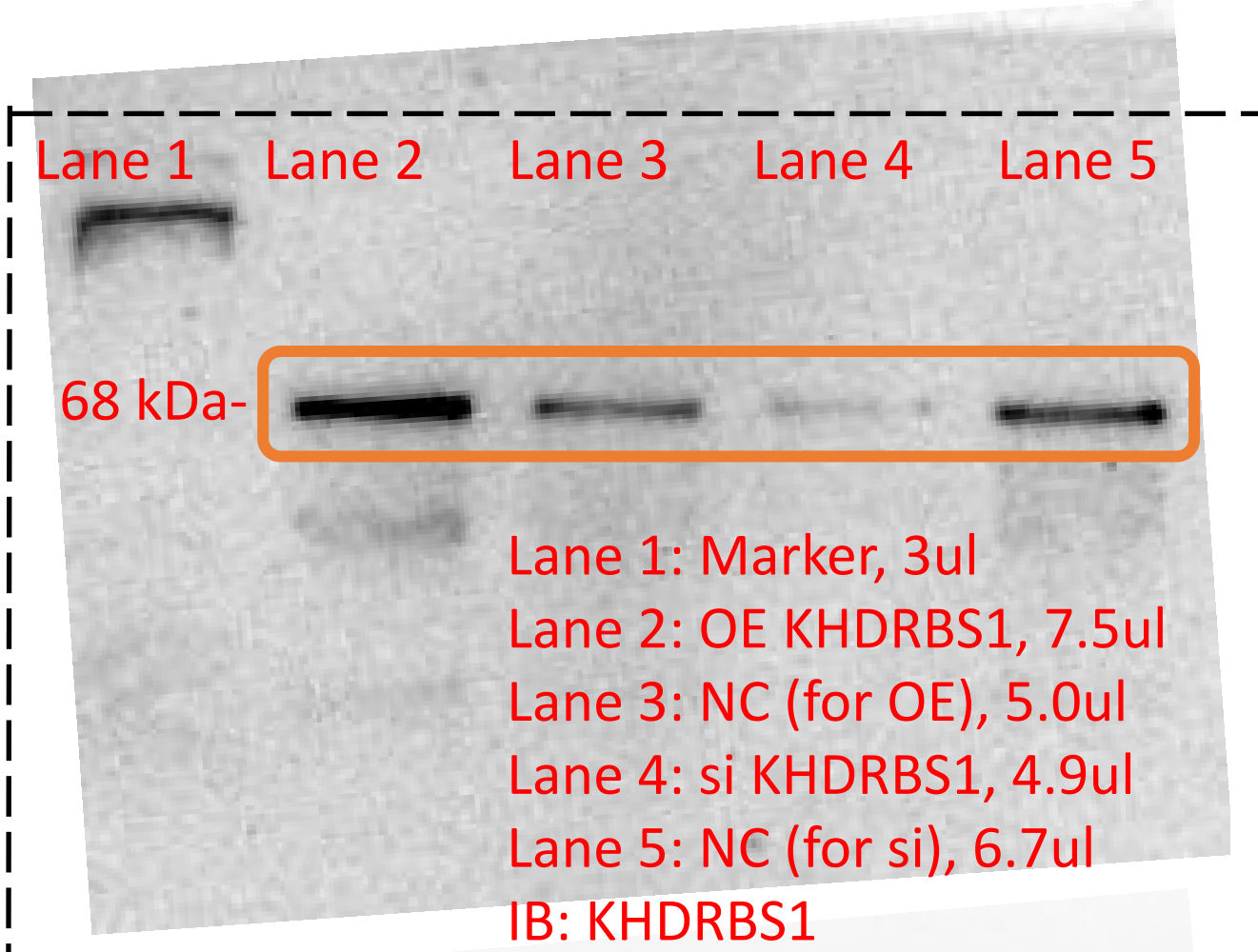
Lane 1
Lane 2
Lane 3
Lane 4
Lane 5
68 kDa-
Lane 1: Marker, 3ul
Lane 2: OE KHDRBS1, 7.5ul
Lane 3: NC (for OE), 5.0ul
Lane 4: si KHDRBS1, 4.9ul
Lane 5: NC (for si), 6.7ul
IB: KHDRBS1

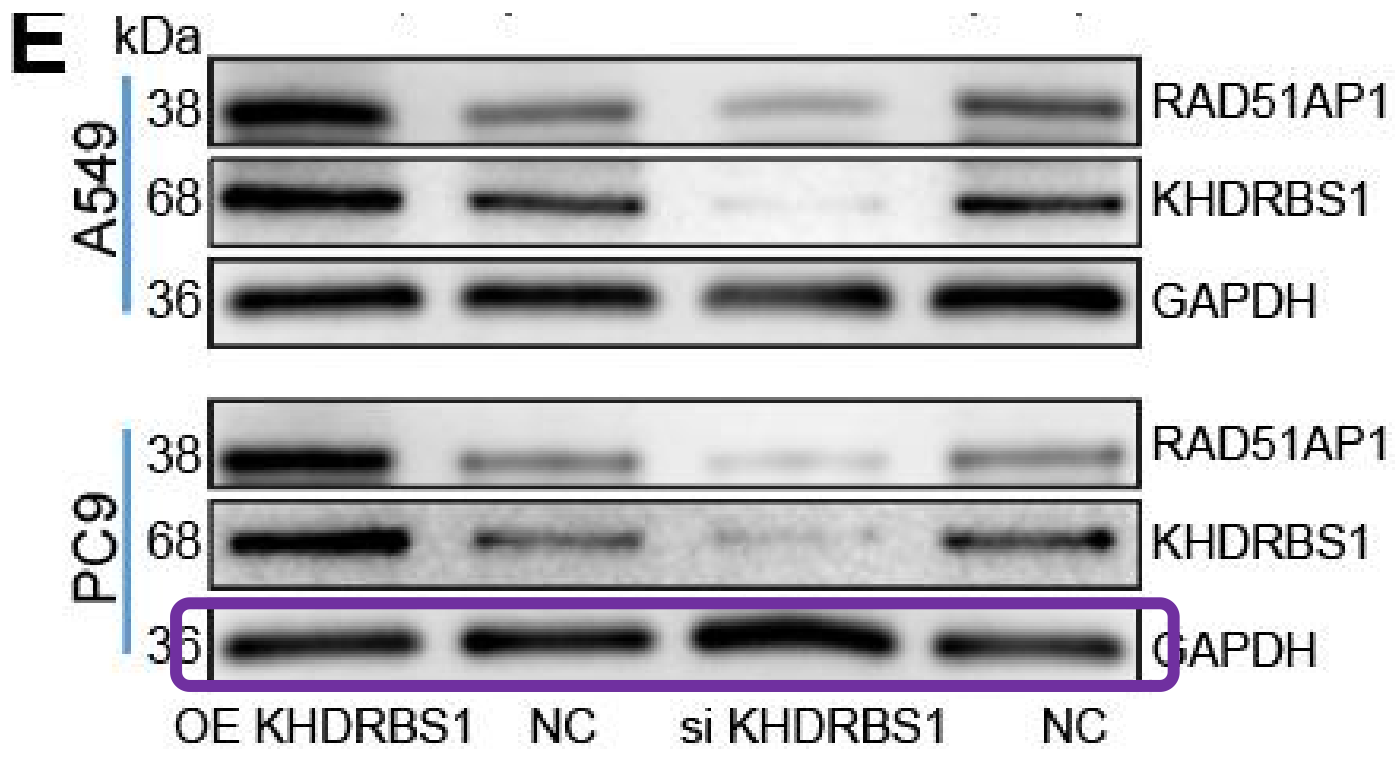
E
kDa
A549
38
68
36
RAD51AP1
KHDRBS1
GAPDH
PC9
38
68
36
RAD51AP1
KHDRBS1
GAPDH
OE KHDRBS1
NC
si KHDRBS1
NC

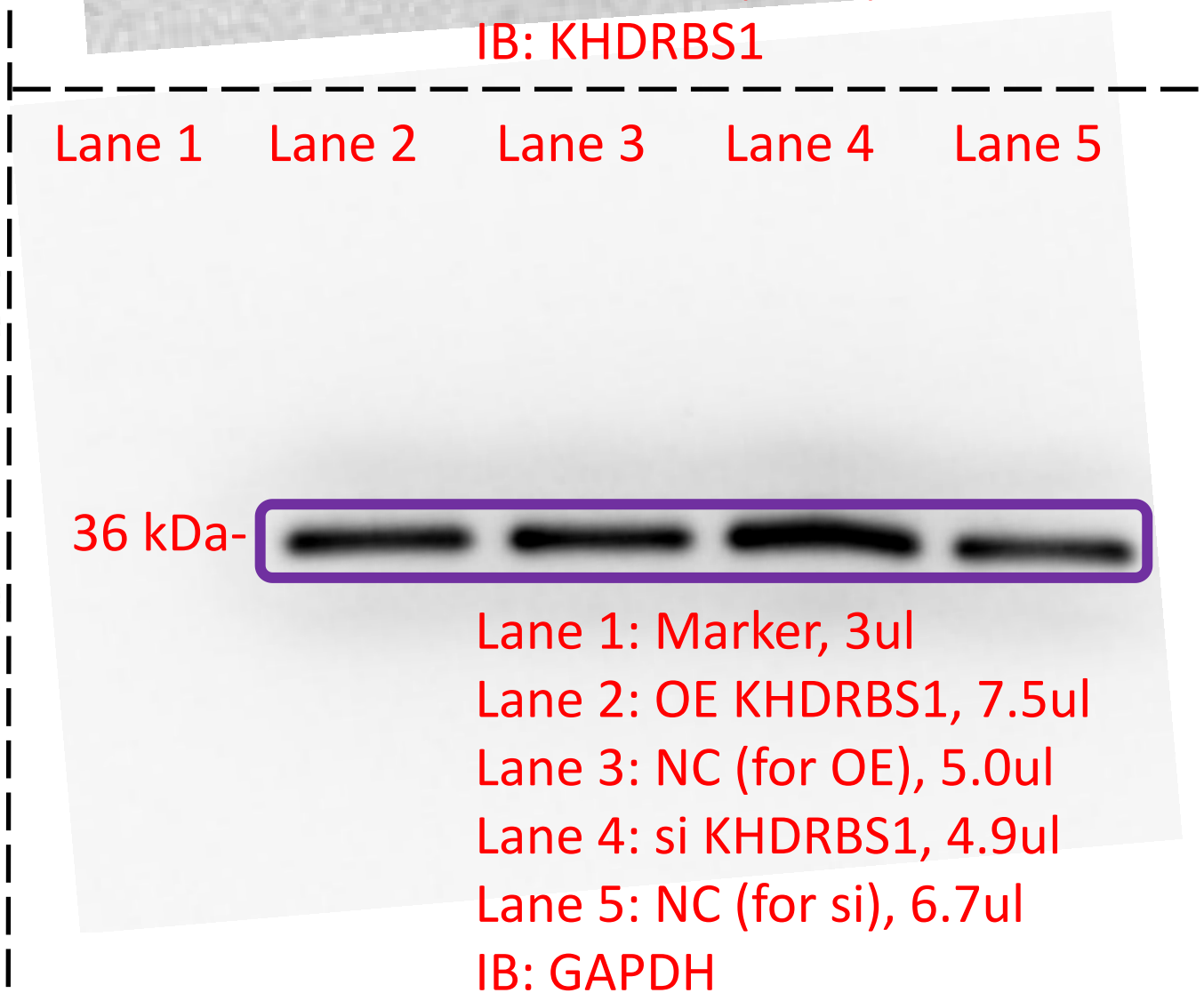
Lane 1
Lane 2
Lane 3
Lane 4
Lane 5
36 kDa-
Lane 1: Marker, 3ul
Lane 2: OE KHDRBS1, 7.5ul
Lane 3: NC (for OE), 5.0ul
Lane 4: si KHDRBS1, 4.9ul
Lane 5: NC (for si), 6.7ul
IB: GAPDH

Figure 7B

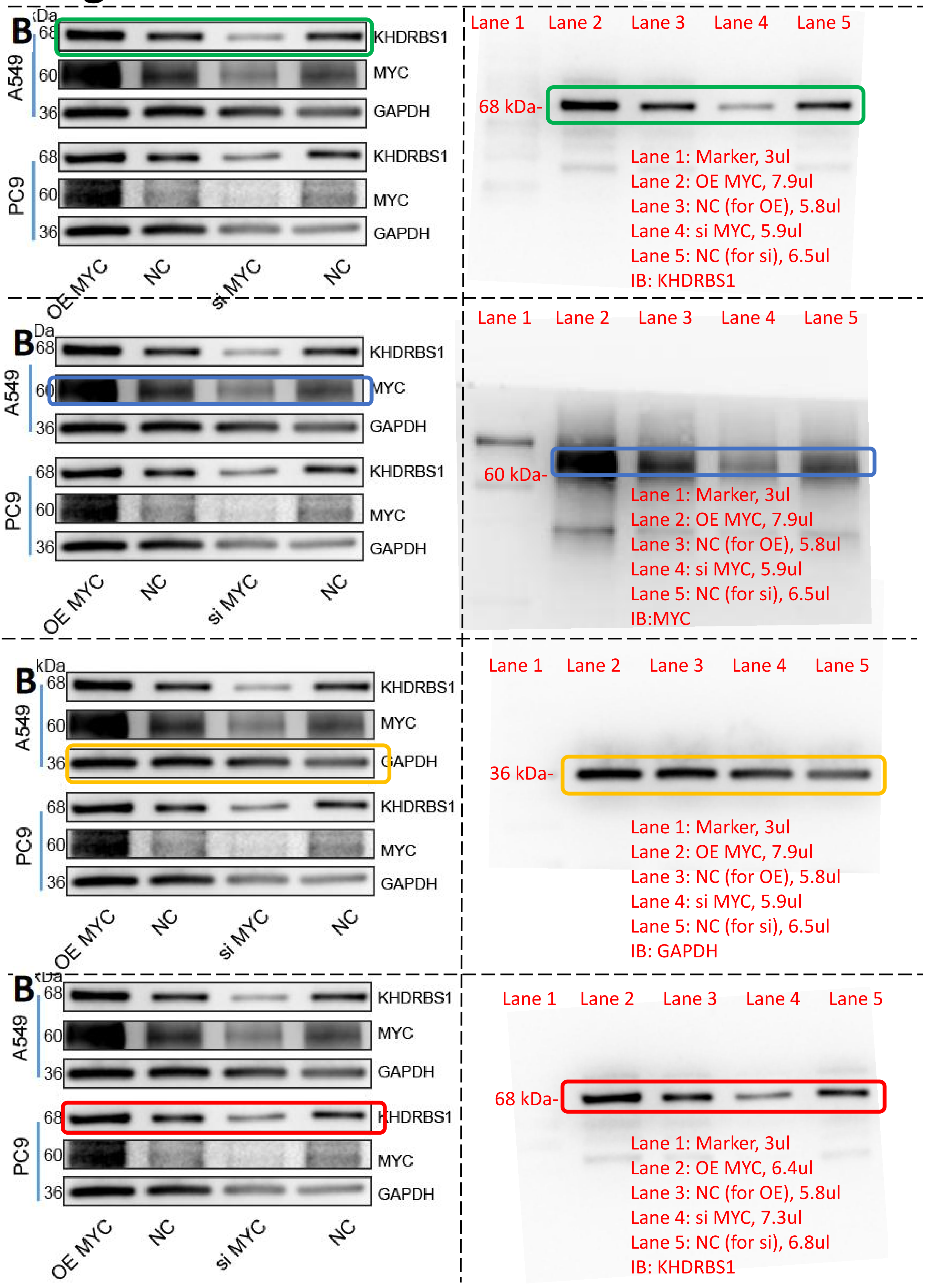
B
kDa
A549
PC9
68
60
36
KHDRBS1
MYC
GAPDH
OE MYC
NC
si MYC
NC
Lane 1
Lane 2
Lane 3
Lane 4
Lane 5
68 kDa-
Lane 1: Marker, 3ul
Lane 2: OE MYC, 7.9ul
Lane 3: NC (for OE), 5.8ul
Lane 4: si MYC, 5.9ul
Lane 5: NC (for si), 6.5ul
IB: KHDRBS1
60 kDa-
Lane 1: Marker, 3ul
Lane 2: OE MYC, 7.9ul
Lane 3: NC (for OE), 5.8ul
Lane 4: si MYC, 5.9ul
Lane 5: NC (for si), 6.5ul
IB:MYC
36 kDa-
Lane 1: Marker, 3ul
Lane 2: OE MYC, 7.9ul
Lane 3: NC (for OE), 5.8ul
Lane 4: si MYC, 5.9ul
Lane 5: NC (for si), 6.5ul
IB: GAPDH
68 kDa-
Lane 1: Marker, 3ul
Lane 2: OE MYC, 6.4ul
Lane 3: NC (for OE), 5.8ul
Lane 4: si MYC, 7.3ul
Lane 5: NC (for si), 6.8ul
IB: KHDRBS1

Figure 7B

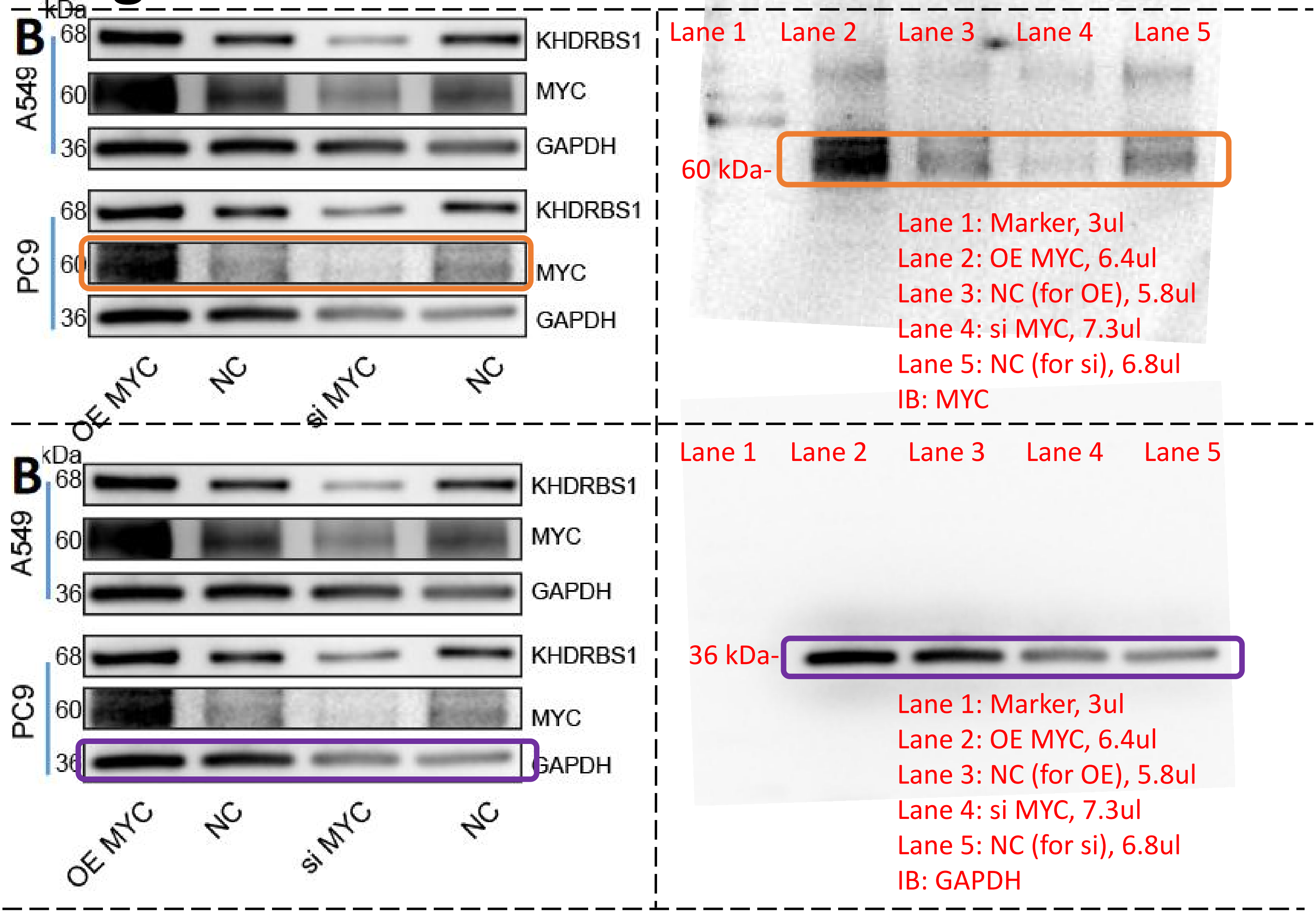

B
kDa
A549
PC9
68
60
36
KHDRBS1
MYC
GAPDH
OE MYC
NC
si MYC
NC
Lane 1
Lane 2
Lane 3
Lane 4
Lane 5
60 kDa-
Lane 1: Marker, 3ul
Lane 2: OE MYC, 6.4ul
Lane 3: NC (for OE), 5.8ul
Lane 4: si MYC, 7.3ul
Lane 5: NC (for si), 6.8ul
IB: MYC
36 kDa-
IB: GAPDH

Figure 7E

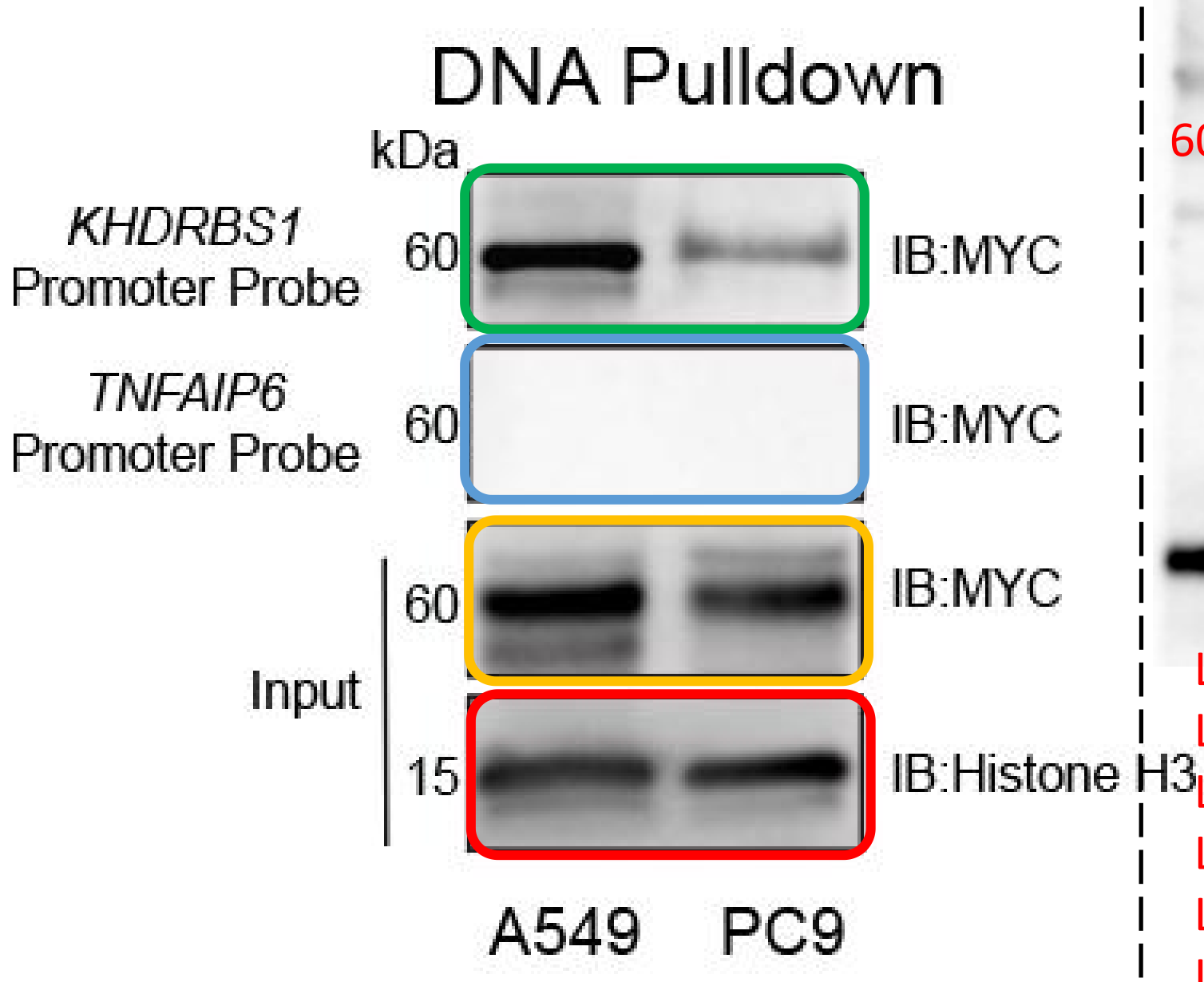

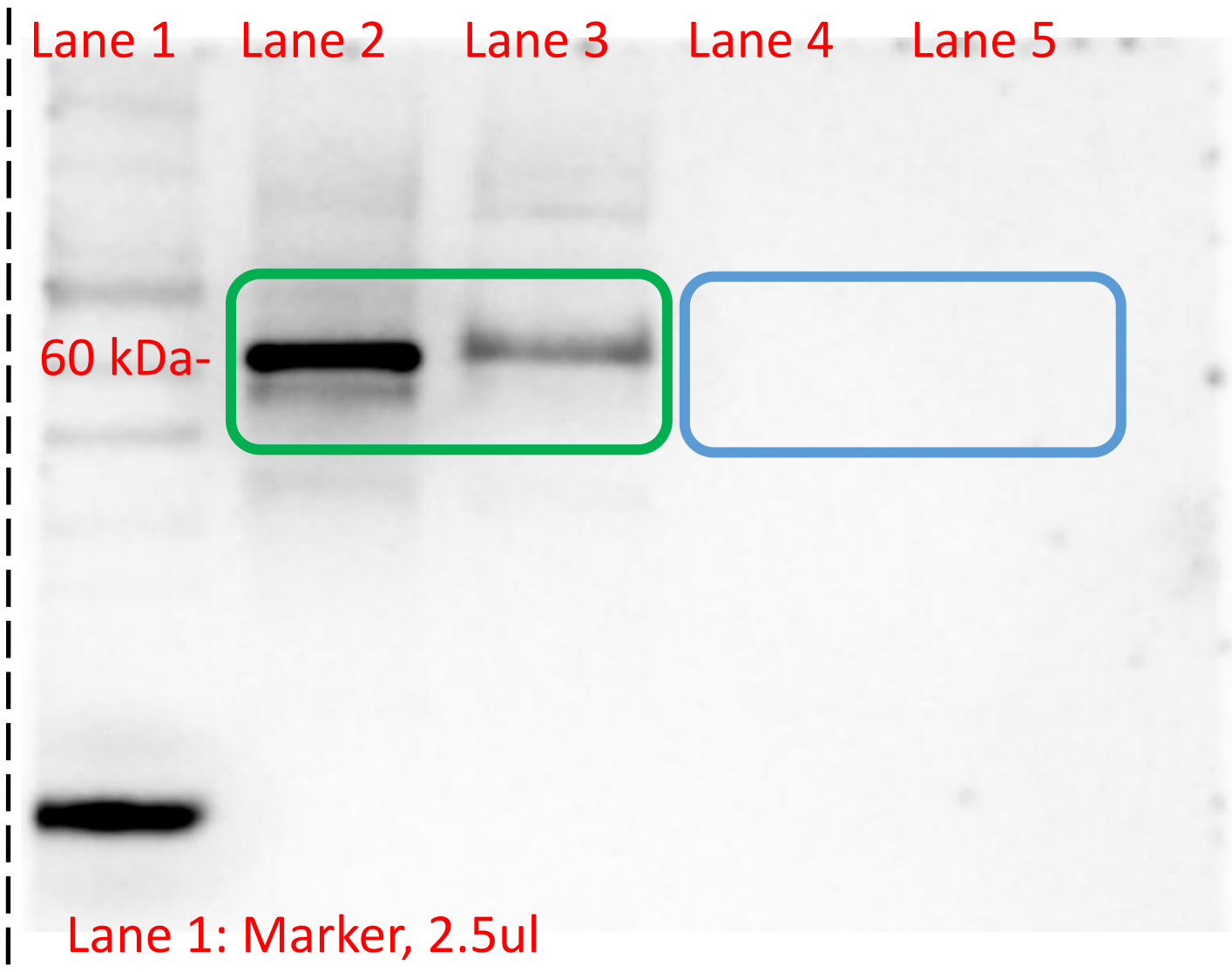

Lane 1: Marker, 2.5ul
Lane 2: *KHDRBS1* Promoter Pulldown (A549), 25ul
Lane 3: *KHDRBS1* Promoter Pulldown (PC9), 25ul
Lane 4: *TNFAIP6* Promoter Pulldown (A549), 25ul
Lane 5: *TNFAIP6* Promoter Pulldown (PC9), 25ul
IB: MYC

PS： Perform elution at a constant 37 °C for 2 h, and mix every 20 min.

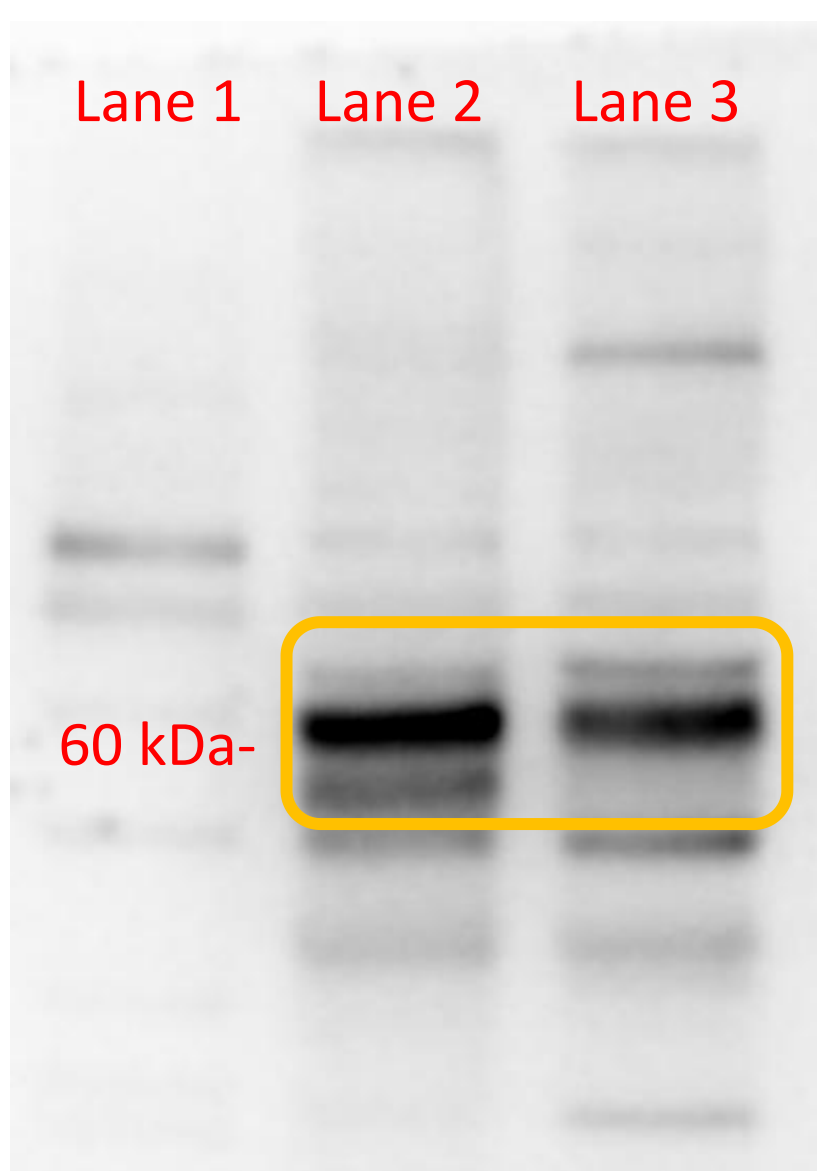

Lane 1: Marker, 2.5ul
Lane 2: Input (A549), 2.5ul
Lane 3: Input (PC9), 2.5ul
IB: MYC

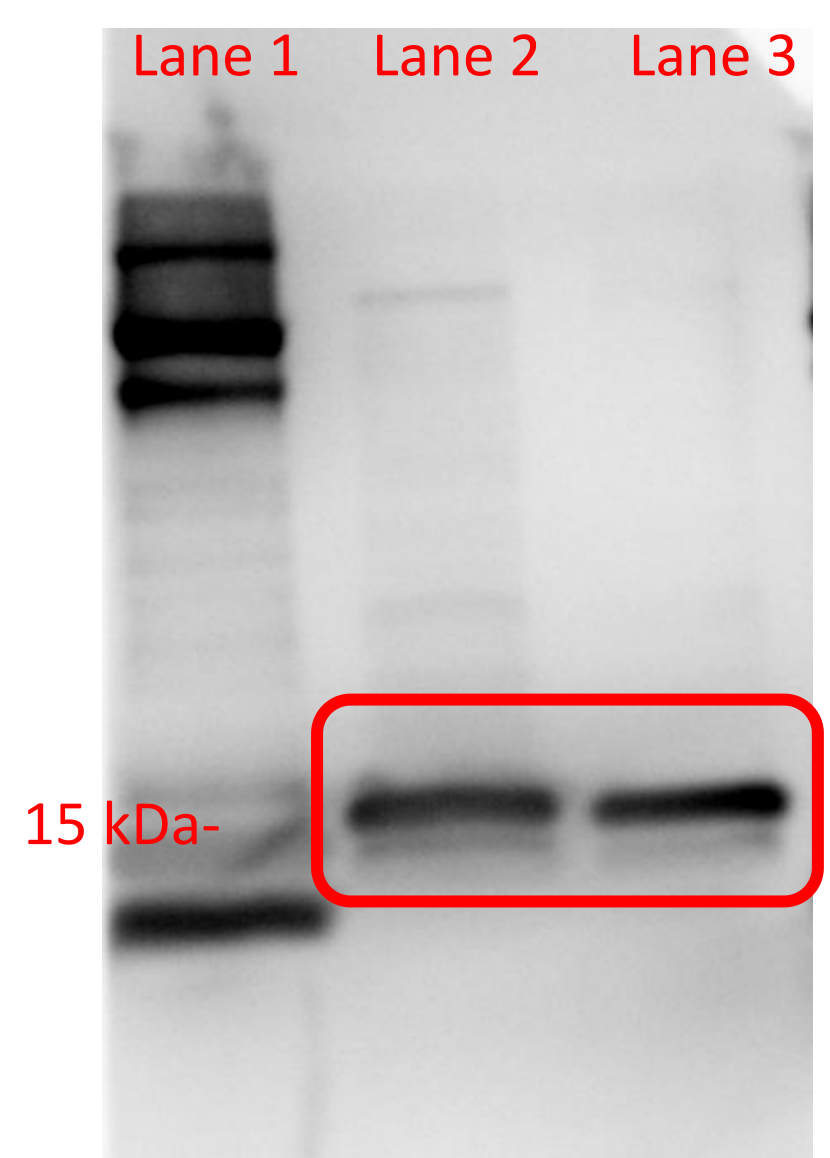

Lane 1: Marker, 2.5ul
Lane 2: Input (A549), 2.5ul
Lane 3: Input (PC9), 2.5ul
IB: Histone H3
