# Supplementary material for: RAD51AP1-MYC-Sam68 positive transcriptional feedback loop maintains lung cancer stemness
Source: iScience. 2026 Aug 31;29(9):117397. doi: 10.1016/j.isci.2026.117397 (PMC13562426; doi:10.1016/j.isci.2026.117397)
Supplement: Supplementary file 2 [file mmc5.zip › Animal Ethics Approval.pdf]

ZYY-DWFL-IRB- 002(F)-01

天津医科大学总医院
实验动物福利伦理委员会审查批件

批件号	IRB2023-DWFL-103
项目名称	肺腺癌干性新机制：RAD51AP1 捕获 ZNF382 并逆转其对 c-MYC 转录的直接抑制作用
项目来源	国家自然科学基金青年项目
研究单位	天津医科大学总医院肺部肿瘤外科
项目负责人	刘仁旺
审查日期	2023.2.24

涉及实验动物研究内容及方案摘要：

1)购买裸鼠，建立 CDX 模型，完善双株细胞验证 RAD51AP1 调控干性表型，RAD51AP1 经由 c-MYC 调控干性的回复实验及验证 ΔRAD51AP1 与 RAD51AP1 调控肺腺癌干性作用的差异等体内实验。取各移植瘤，检测各移植瘤中 RAD51AP1、c-MYC 等相关分子的表达。

2）构建 Rad51ap1$^{-/-}$小鼠，以 WT 小鼠为对照，将 4%的乌拉坦溶液诱导肺癌模型，应用 9.4T 小动物 MRI 对小鼠肺部进行检测，记录成瘤小鼠数，成瘤时间，及成瘤大小数目，绘制成瘤曲线，同时记录小鼠死亡时间，进行生存分析。取肺部肿瘤组织，完善相关分子检测。

批准文件：

研究方案

审查意见：以上《动物实验方案》经过天津医科大学总医院实验动物福利伦理委员会审查，符合动物保护、动物福利和伦理原则，符合国家实验动物福利伦理的相关规定，同意申报该研究。

天津医科大学总医院

实验动物福利伦理委员会

2023 年 2 月 24 日
