# Supplementary material for: RAD51AP1-MYC-Sam68 positive transcriptional feedback loop maintains lung cancer stemness
Source: iScience. 2026 Aug 31;29(9):117397. doi: 10.1016/j.isci.2026.117397 (PMC13562426; doi:10.1016/j.isci.2026.117397)
Supplement: Supplementary file 2 [file mmc5.zip › Human Ethics Approval.pdf]

ZYY-IRB-SOP-016(F)-002-04

# 天津医科大学总医院医学伦理委员会审查批件

| | |
|---|---|
| 批件号 | IRB2023-KY-074 |
| 项目名称 | 肺腺癌干性新机制：RAD51AP1 捕获 ZNF382 并逆转其对 c-MYC 转录的直接抑制作用 |
| 项目来源 | 国家自然科学基金青年项目 |
| 研究单位 | 天津医科大学总医院肺部肿瘤外科 |
| 项目负责人 | 刘仁旺 |
| 审查日期 | 2023.2.24 |

涉及人的生物医学研究内容及方案摘要：

① 样本资料收集：联合前期 60 例临床样本，收集天津医科大学总医院肺部肿瘤外科 2017 年-2020 年手术切除肺腺癌患者组织 140 例，共计 200 例。要求组织切片丰富、临床信息完整、病理学特征清晰及预后随访资料齐全。同时，收集 60 例手术患者新鲜组织及癌旁组织，完善相关临床资料及随访信息。

② 应用免疫组化检测各切片中 RAD51AP1 及 c-MYC 表达量，应用 DNA pulldown WB 检测新鲜组织中与 c-MYC 启动子结合的 ZNF382 蛋白量。

③ 统计分析：应用 COX 回归模型及 logistic 回归分析等统计学方法分析上述检测指标与临床各因素做单因素/多因素分析和预后关联分析，明确临床意义。

批准文件：

1、研究方案　　　2、知情同意书

审查意见：经审查，《肺腺癌干性新机制：RAD51AP1 捕获 ZNF382 并逆转其对 c-MYC 转录的直接抑制作用》符合国家卫生和计划生育委员会“涉及人的生物医学研究伦理审查办法”（2016 年）相关规定，同意申报该研究。

天津医科大学总医院
医学伦理委员会
2023 年 2 月 24 日
